# RNA-seq Reveals Novel Transcriptome of Genes and Their Isoforms in Human Pulmonary Microvascular Endothelial Cells Treated with Thrombin

**DOI:** 10.1371/journal.pone.0031229

**Published:** 2012-02-16

**Authors:** Li Qin Zhang, Dilyara Cheranova, Margaret Gibson, Shinghua Ding, Daniel P. Heruth, Deyu Fang, Shui Qing Ye

**Affiliations:** 1 Department of Pediatrics, Children's Mercy Hospitals and Clinics, University of Missouri School of Medicine, Kansas City, Missouri, United States of America; 2 Department of Biomedical and Health Informatics, Children's Mercy Hospitals and Clinics, University of Missouri School of Medicine, Kansas City, Missouri, United States of America; 3 Dalton Cardiovascular Research Center and Department of Biological Engineering, University of Missouri, Columbia, Missouri, United States of America; 4 Department of Pathology, Northwestern University Feinberg School of Medicine, Chicago, Illinois, United States of America; Wayne State University, United States of America

## Abstract

The dysregulation of vascular endothelial cells by thrombin has been implicated in the development of a number of pathologic disorders such as inflammatory conditions, cancer, diabetes, coronary heart disease. However, transcriptional regulation of vascular endothelial cells by thrombin is not completely understood. In the present study, Illumina RNA-seq was used to profile the transcriptome in human pulmonary microvascular endothelial cells (HMVEC-L) treated with thrombin for 6 h to gain insight into thrombin's direct effects on the endothelial function. Out of 100 million total reads from a paired end sequencing assay, 91–94% of the reads were aligned to over 16,000 genes in the reference human genome. Thrombin upregulated 150 known genes and 480 known isoforms, and downregulated 2,190 known genes and 3,574 known isoforms by at least 2 fold. Of note, thrombin upregulated 1,775 previously unknown isoforms and downregulated 12,202 previously unknown isoforms by at least 2 fold. Many genes displayed isoform specific differential expression levels and different usage of transcriptional start sites after the thrombin treatment. The cross comparisons between our RNA-seq data and those of DNA microarray analysis of either 6 h thrombin treated HUVEC or 5 h TNFα treated HMVEC have provided a significant overlapping list of differentially expressed genes, supporting the robust utility of our dataset. Further in-depth follow-up analysis of the transcriptional regulation reported in this study may shed light on molecular pathogenic mechanisms underlying thrombin mediated endothelial dysfunction in various diseases and provide new leads of potential therapeutic targets.

## Introduction

Endothelial cells lining the inner surface of microvessels form a semipermeable barrier that actively participates in blood–tissue exchange of plasma fluid, proteins and cells. The precise regulation of endothelial barrier and function is essential for maintaining circulatory homeostasis and the physiological function of different organs. The dysregulation of microvascular barrier and function represent crucial events in the development of a variety of disease processes, such as adult respiratory distress syndrome, ischemia–reperfusion injury, diabetic vascular complications, and tumor metastasis [1].

It is now understood that while increased circulating levels of soluble mediators such as thrombin, pro-inflammatory cytokines such as TNFα, and immune cells such as neutrophils are a hallmark of host defense against injury, most of these mediators target the vascular endothelium to produce vasoactive and cytotoxic effects [1]. Thrombin is a serine protease. Besides its central role in the coagulation cascade acting as both a coagulant factor and an anticoagulant factor, thrombin can trigger important cellular effects via a protease-activated receptor pathway or receptor independent pathways [2]. Thrombin can stimulate endothelial cells and regulate the expression, release and activation of a number of biological mediators. Thrombin is known for its endothelial hyperpermeability effect. Thrombin can protect endothelial cells from apoptosis [3].. Thrombin plays an important role in angiogenesis and is thus involved in tumor progression and metastasis [4]. The list of physiological and pathological roles of thrombin is still expanding. The understanding of these events at a molecular level will provide new targets of therapeutic interventions in disease states.

With the advent of next generation DNA sequencing technologies [5], such as RNA-seq, a more comprehensive and accurate transcriptome analysis has become feasible and affordable. RNA-seq first sequences complementary DNA (cDNA) in short fragments and subsequent mapping of such short sequence fragments (reads) onto the reference genome. RNA-Seq enables identification of transcription initiation sites (TSSs) and new splicing variants, and it permits a precise quantitative determination of exon and splicing isoform expression [6]. In the present study, we performed a comprehensive transcriptome analysis in human pulmonary microvascular endothelial cells (HMVEC-L) treated with or without thrombin using the Illumina RNA-Seq technique. We found significant differences in gene isoform expression levels, alternated use of promoters and transcription start sites between control and thrombin treated HMVEC-L. These data provided broader and deeper insights into thrombin's transcriptional regulation on the vascular endothelial functions and it also provides rich reagents for further experimentations.

## Results

### Quality analysis of RNA-Seq data

Real-time analysis of the sequencing run, including several measurements, was performed by the Illumina HiSeq Control Software. Clusters of identical sequences were generated on the Illumina cBot and the number of those clusters was reported, along with the percentage of those clusters passing an internal quality filter. Across the 5 samples, between 599,000 and 621,000 raw clusters were detected, with a median of 603,000 clusters per lane. Between 89.5% and 92.1% of those clusters passed the filter, with a median of 91.3% of the clusters passing the filter. Each lane was aligned in real-time with the PhiX genome and between 0.6% and 0.7% of the clusters aligned. Our control lane of PhiX produced 357,000 clusters with 96.9% passing the filter and 99.1% aligning to the PhiX genome. All these values were within the recommended limits established by Illumina.

**Figure 1:**
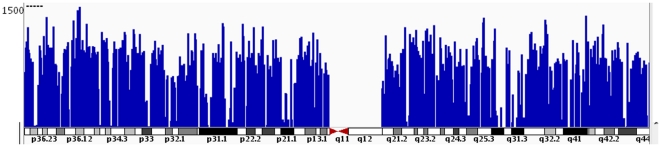
A transcription profile of HMVEC cells (Control A) for chromosome 1. The RNASeq read density plotted along chromosome 1 is shown. Average alignment was computed by igvtools. The coverage values were measured along intervals of the genome. These intervals vary in size depending on how variable the read density is for a particular genomic location. Each bar represents the log_2_ frequency of reads plotted against chromosome coordinates.

Post-run quality analysis of RNA-Seq Data was carried out as described by Twine et al. [7]. The total number of reads produced from each sample was between 97,478,180 and 105,351,222, with a median across all samples of 102,568,132 (Table 1). The difference in the number of reads between the control samples and the thrombin samples was not statistically significant (Student's t-test, p = 0.88). To assess the quality of the reads, data was pulled from the TopHat log files as well as the output files. Between 0.31% and 0.34% of the reads were removed due to low quality before mapping to the reference genome began. Between 88.4% and 94.2% of the total reads mapped to the human genome. To ensure the uniform coverage across the genome, the data was visualized using a local copy of the Integrative Genomics Viewer. We mapped the control A reads against chromosome 1 (Figure 1). The average alignment was computed across the genome and those alignment scores were log-transformed (base 2) to better visualize the full range of the data. As expected, no reads mapped to the centromere or areas of the chromosome without genes.

**Table 1:** RNA-Seq sequence reads mapping to UCSC Human genome build 19 by TopHat v1.3.0/Bowtie v0.12.7.

	Control			Thrombin			
	A	B	Average	A	A2	B	Average
Total reads	102,399,460	102,568,132	102,483,796	105,351,222	103,170,324	97,478,180	101,999,909
Reads removed	0.32%	0.31%	0.31%	0.33%	0.32%	0.34%	0.33%
Read aligned to reference genome	94.2%	93.4%	93.5%	93.5%	92.4%	88.4%	91.5%

Total reads and the percentage of those reads removed due to low quality and aligned to hg19 by TopHat. TopHat allows two mismatches when aligning to a reference genome.

### Differentially expressed genes

After mapping the sequencing reads to the reference genome with TopHat, transcripts were assembled and their relative expression levels were calculated with Cufflinks in FPKM. Then, the sub-program, Cuffdiff was used to calculate the differential expression on both a gene level and a transcript level, as well as the calculation of alternative promoter usage and alternative splicing. Cufflinks calculates the differential gene expression with the ratio of the thrombin treated group to the control group for every gene and transcript along with the statistical significance of the values.

Overall, there are 16,636 expressed genes in the control cells and 16,357 expressed genes in thrombin treated genes, which were aligned to the reference genome (Table 2). There are 783 genes, whose expressions were only detected in the control cells, while 504 genes whose expressions were only detected in thrombin treated cells. The differentially expressed genes in thrombin treated cells over the controls with statistically significant fold changes ranged from −16.32 to 94.23. There are 152 upregulated- and 2190 downregulated- genes in thrombin treated cells with at least 2 fold change over the control cells (Table 2). As for known isoforms, there are 26,807 in the control cells and 26,300 in thrombin treated genes. Among them, there are 1,492 known isoforms, whose expressions were detected only in the control cells, while 985 known isoforms whose expressions were detected only in thrombin treated cells. The differentially expressed known isoforms in thrombin treated cells over the controls with statistically significant fold changes ranged from −126.25 to 962.57. There are 480 upregulated- and 3,574 downregulated- known isoforms in thrombin treated cells by at least 2 fold change over the control cells (Table 2). For novel isoforms whose annotations are not known in the current reference gene or transcript database, there are 25,880 in the control cells and 25,886 in thrombin treated genes. Among them, there are 418 novel isoforms, whose expressions were only detected in the control cells, while 424 novel isoforms whose expressions were only detected in thrombin treated cells. The differentially expressed novel isoforms in thrombin treated cells over the controls with statistically significant fold changes ranged from −927.74 to 431.33. There are 1,775 upregulated- and 12,202 downregulated- novel isoforms in thrombin treated cells by at least 2 fold change over the control cells (Table 2).

**Table 2:** Gene/Isoform expression summary.

Genes		
	Control	Thrombin
Total Genes Expressed	16,636	16,357
Control Only	783	
Thrombin Only		504
Up-regulated (2-fold or greater difference)		152
Down-regulated (2-fold or greater difference)		2,190

Known Isoforms		
	Control	Thrombin
Total Known Isoforms Expressed	26,807	26,300
Control Only	1,492	
Thrombin Only		985
Up-regulated (2-fold or greater difference)		480
Down-regulated (2-fold or greater difference)		3,574

Novel Isoforms		
	Control	Thrombin
Total Novel Isoforms Expressed	25,880	25,886
Control Only	418	
Thrombin Only		424
Up-regulated (2-fold or greater difference)		1,775
Down-regulated (2-fold or greater difference)		12,202

Genes, known isoforms and novel isoforms expressed in control and thrombin-treated HMVEC cells. Expression determined by CuffDiff, after Benjamini-Hochberg correction. The fold change is the ratio of thrombin FPKM to control FPKM.

**Figure 2:**
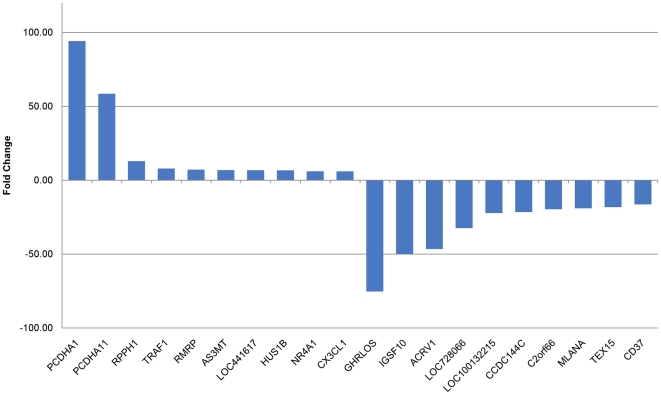
Top 10 up-/down-regulated genes in thrombin treated HMVEC. The differentially expressed genes in thrombin treated HMVEC vs those in control cells were determined by CuffDiff, after Benjamini-Hochberg correction. The fold change is the ratio of FPKM of those genes in thrombin treated HMVEC to FPKM of those genes in control cells. The differentially expressed genes were ranked on their fold change and the 10 with the highest or lowest fold changes are shown here. The full name of each gene symbol is provided in text and Table S1.

Top 10 up- and down- regulated genes in thrombin treated HMVEC cells are presented in Figure 2. The expanded list of top 50 up- and down- regulated genes in thrombin treated HMVEC cells are presented in the supplement (Table S1). Protocadherin α1 (PCDHA1) and α11 (PCDHA11) stand out as the top two most upregulated genes in thrombin treated HMVEC cells, respectively. PCDHA1 was upregulated by 94 fold, while PCDHA11 was upregulated by 59 fold. Among the top upregulated genes, TNF receptor-associated factor 1 (TRAF1) and chemokine C-X3-C motif ligand 1 (C-X3-CL1), both of which are associated with the inflammatory response, were upregulated by 8 and 6 fold, respectively. Arsenic (+3 oxidation state) methyltransferase (AS3MT), which is involved in oxidative stress, was upregulated by 7 fold. Ghrelin opposite strand RNA (GHRLOS) and immunoglobulin superfamily member 10 (IGSF 10) are the top two most downregulated genes in thrombin treated HMVEC cells. They were downregulated by 75 and 50 fold, respectively. The majority of genes presented in Figure 2 have not yet been identified in the literature to be differentially regulated by thrombin. One fourth of those top regulated genes in Figure 2 (LOC441617, LOC728066, LOC100132215 and C2orf66d) are currently not annotated. In the top 50 up- and down- regulated genes in thrombin treated HMVEC cells in Table S1, about 10% of them are not annotated.

**Figure 3:**
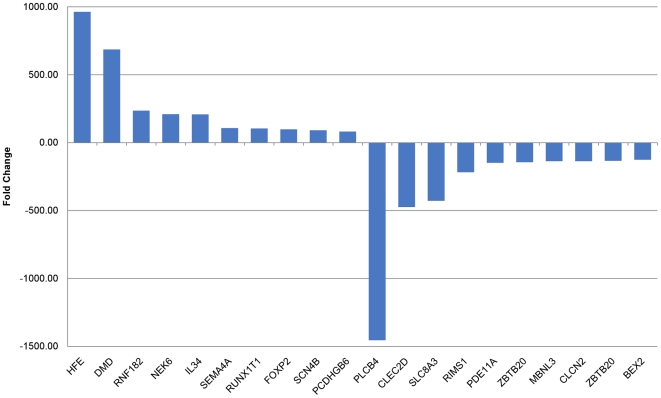
Top 10 up-/down-regulated gene isoforms in thrombin treated HMVEC. The differentially expressed gene isoforms in thrombin treated HMVEC vs those in control cells were determined by CuffDiff, after Benjamini-Hochberg correction. The fold change is the ratio of FPKM of those gene isoforms in thrombin treated HMVEC to FPKM of those in control cells. The differentially expressed gene isoforms were ranked on their fold change and the 10 with the highest or lowest fold changes are shown here. The full name of each gene isoform symbol is provided in text and Table S2.

Top 10 up- and down- regulated known isoforms in thrombin treated HMVEC cells are presented in Figure 3. The expanded list of top 50 up- and down- regulated known isoforms in thrombin treated HMVEC cells are presented in the supplement (Table S2). The fold change among top 50 up- regulated known isoforms in thrombin treated HMVEC cells varies from 12 to 963. The fold change among top 50 down- regulated known isoforms in thrombin treated HMVEC cells varies from −24 to −1455. Consistent with the finding that PCDHA1 and PCDHA11 are the top regulated genes (Table 1), both genes are among the top regulated known isoforms in thrombin treated HMVEC cells (Figure 3 and Table S2). Another two known isoforms of the protocadeherin gene family [protocadherin gamma gene B6 (PCDHGB6) and PCDHA3] were also upregulated by 82 and 22 fold, respectively. One of the hemochromatosis (HFE) gene isoforms was the highest upregulated isoform (983 fold) by thrombin. HFE is known to be involved in iron metabolism. It may also be involved in a tissue injury and reparative process [8]. Phospholipase C- beta 4 (PLCB4) was the highest down regulated isoform (−1455 fold) by thrombin. Phospholipase C-beta hydrolyses phosphatidylinositol 4,5-bisphosphate and generates inositol 1,4,5-trisphosphate in response to activation of various G protein-coupled receptors. PLCB4 was implicated in the delayed [Ca2+]i increase in response to the stimulation by thrombin [9]. Among the top ten up- and down-regulated known gene isoforms by thrombin are three presumable transcription factors (runt-related transcription factor 1, forkhead box P2, zinc finger and BTB domain containing 20) and two ion channel gene isoforms (sodium channel and voltage-gated type IV beta, and sodium/calcium exchanger member 3). Two different isoforms of the gene *ZBTB20* are listed among the top ten down-regulated known gene isoforms by thrombin. They differ in the region of their untranslated first exons.

The top 10 up- and down- regulated novel isoforms in thrombin treated HMVEC cells are presented in Table 3. The expanded list of top 50 up- and down- regulated novel isoforms in thrombin treated HMVEC cells are presented in the supplement (Table S3). The fold change among top 50 upregulated novel isoforms in thrombin treated HMVEC cells varies from 15 to 431. The fold change among top 50 downregulated novel isoforms in thrombin treated HMVEC cells varies from −44 to −928. Integrin alpha FG-GA repeat containing 2 (ITFG2), a novel isoform of integrin alpha, was the highest upregulated novel isoform (431 fold) by thrombin. Integrins are heterodimeric membrane receptors consisting of a and b subunits, that play a pivotal role to functionally integrate the extracellular matrix with the cytoskeleton across the plasma membrane [10]. Three isoforms of Zinc finger protein 518A were among the top one, three and five highest down regulated novel isoforms by thrombin (−928, 738, and 262 fold) respectively. Two upregulated novel isoforms come from two cytoskeleton proteins: non-erythrocytic 2 spectrin beta (*SPTBN2) and* fibrillin 2 (FBN2). More than one fourth of top 10 up- and down- regulated novel isoforms in thrombin treated HMVEC cells (6/20) are from unannotated genes (Table 3). Three different novel isoforms of the gene *ZNF518A* are listed among top ten downregulated unknown gene isoforms by thrombin. All these three novel isoforms are longer than any known isoforms of the gene *ZNF518A*.

**Table 3:** Top ten up- and down- regulated novel isoforms in thrombin treated HMVEC Cells.

Gene	Description	Coordinates	Length	FPKM Control	FPKM Thrombin	Fold Change	p-value	Ensembl Gene ID
*ITFG2*	integrin alpha FG-GA repeat containing 2	chr12:2921756–2939432	2257	0.00521437	2.2491	431.33	2.87E-05	ENSG00000111203
*SEC16A*	SEC16 homolog A (S. cerevisiae)	chr9:139334541–139378220	8173	0.0491136	7.40764	150.83	0	ENSG00000148396
*PMS2P11*	postmeiotic segregation increased 2 pseudogene 11	chr7:76589124–76681310	668	0.00371097	0.359467	96.87	0	ENSG00000241350
-	Genes nearby: *ZFHX3 RNU7-71P*	chr16:72816785–73092558	5767	0.00181948	0.0994668	54.67	0	ENSG00000140836 ENSG00000251868
*SPTBN2*	spectrin, beta, non-erythrocytic 2	chr11:66449988–66496487	8426	0.0542375	2.86062	52.74	0	ENSG00000173898
*MCAM*	melanoma cell adhesion molecule	chr11:119170201–119187947	1795	0.514138	26.8537	52.23	0	ENSG00000076706
*FBN2*	fibrillin 2	chr5:127593698–127873129	4871	0.00400303	0.187473	46.83	0.0117604	ENSG00000138829
*PHF21A*	PHD finger protein 21A	chr11:45950885–46143027	7299	0.0236083	1.05125	44.53	0	ENSG00000135365
-	Genes nearby: none	chr19:56115009–56128567	3736	0.00705679	0.306568	43.44	0.0001756	
-	Genes nearby: none	chr19:56115009–56128567	947	0.00746101	0.321088	43.04	0	
*ZNF518A*	zinc finger protein 518A	chr10:97889630–97924859	9138	0.313598	0.000338024	−927.74	0.018282	ENSG00000177853
*MYST4*	MYST histone acetyltransferase (monocytic leukemia) 4	chr10:76585061–76792325	7557	0.124027	0.000147896	−838.61	0	ENSG00000156650
*ZNF518A*	zinc finger protein 518A	chr10:97889630–97924859	9351	0.319225	0.000432489	−738.11	0.0194973	ENSG00000177853
-	Genes nearby: *SENP7 ZNF90P1*	chr3:101043117–101232085	452	1.25099	0.003021	−414.1	0	ENSG00000138468 ENSG00000241738
*ZNF518A*	zinc finger protein 518A	chr10:97889630–97924859	9452	0.216304	0.000826257	−261.79	0.002249	ENSG00000177853
-	Genes nearby: none	chr10:27855765–27870612	1031	0.27927	0.00107217	−260.47	0	
*RBMX*	RNA binding motif protein, X-linked	chrX:135951349–135962911	4282	3.57287	0.0152633	−234.08	0	ENSG00000147274
*E2F6*	E2F transcription factor 6	chr2:11584508–11606270	1439	0.374663	0.00191853	−195.29	0.0034366	ENSG00000169016
*PTBP2*	polypyrimidine tract binding protein 2	chr1:97187347–97280539	3131	0.505582	0.00259479	−194.84	0.0153448	ENSG00000117569
-	Genes nearby: *NTPCR PCNXL2*	chr1:233086321–233431177	1397	0.315314	0.00170047	−185.43	0	ENSG00000135778 ENSG00000135749

Novel Isoforms significantly differentially expressed as determined by CuffDiff, after Benjamini-Hochberg correction. The fold change is the ratio of thrombin FPKM to control FPKM. The novel isoforms were ranked on their fold change and the 10 with the highest or lowest fold changes are listed here.

### Network and pathway analysis of differentially expressed genes

The differentially expressed gene lists of a 2-fold or greater change after the thrombin treatment were submitted to Ingenuity Pathway Analysis (IPA) v9.0-3211 (Ingenuity Systems, Inc., Redwood City, CA). Networks of Network Eligible Molecules, as described in the Method section, were then algorithmically generated based on their connectivity. Networks affected by upregulated genes/isoforms after thrombin treatment of HMVEC cells are listed in Table 6. Networks affected by downregulated genes/isoforms after thrombin treatment of HMVEC cells are listed in Table 7. Notably, inflammatory response is among the top networks of both upregulated genes and isoforms by thrombin (Table 4). Some of genes involved in anti-inflammatory response were downregulated by thrombin (Table 5). This could provide a molecular insight into the known notion that thrombin plays a significant role in inflammatory diseases [11]. Other top affected networks are Cell-To-Cell Signaling and Interaction, Tissue Morphology, Cellular Movement (Table 4 and 5). These observations are consistent with known functions of thrombin that can cause dissociation of cell-cell junctions between endothelial cells as well as cytoskeleton contraction, leading to a widened intercellular space that facilitates transendothelial flux [1].

**Table 4:** Networks affected by up-regulated genes/isoforms after thrombin treatment of HMVEC cells.

Up regulated genes		
Networks	Score	Genes
Inflammatory Response, Cell-To-Cell Signaling and Interaction, Hematological System Development and Function	41	34
Inflammatory Response, Inflammatory Disease, Skeletal and Muscular Disorders	38	32
Cellular Growth and Proliferation, Tissue Morphology, Cellular Movement	24	24

Up regulated isoforms		
Networks	Score	Genes
Hematological System Development and Function, Tissue Morphology, Cellular Growth and Proliferation	102	70
Cell Death, Cell Cycle, Cellular Growth and Proliferation	45	45
Cellular Growth and Proliferation, Cellular Movement, Cell Death	23	30
Inflammatory Disease, Connective Tissue Disorders, Skeletal and Muscular Disorders	20	28

Networks significantly down-regulated by thrombin treatment as determined by Ingenuity Pathway Analysis. The score is based on the p-value calculation. Networks with a score of 15 or greater were defined as significant.

**Table 5:** Top networks affected by down-regulated genes/isoforms after thrombin treatment of HMVEC cells.

Down regulated genes		
Networks	Score	Genes
DNA Replication, Recombination, and Repair, Molecular Transport, Cellular Growth and Proliferation	27	33
Cell Cycle, Cancer, Cellular Growth and Proliferation	22	33
Cancer, Cellular Development, Organismal Injury and Abnormalities	16	33
Cellular Growth and Proliferation, Cancer, Cell Cycle	16	33
Cell Death, Cellular Movement, Cellular Growth and Proliferation	16	33
Inflammatory Response, Antimicrobial Response, Cell-To-Cell Signaling and Interaction	15	32

Down regulated isoforms		
Networks	Score	Genes
Cancer, Cell Cycle, Cell Death	60	70
Connective Tissue Disorders, Genetic Disorder, Ophthalmic Disease	25	33
Cell Cycle, Cellular Growth and Proliferation, Cardiovascular System Development and Function	21	33
Cell Death, Cell Cycle, Post-Translational Modification	18	42
Cell Death, Cellular Growth and Proliferation, Cancer	16	40
Cell Death, Organismal Survival, Cellular Development	15	39

Networks significantly down-regulated by thrombin treatment as determined by Ingenuity Pathway Analysis. The score is based on the p-value calculation. Networks with a score of 15 or greater were defined as significant.

**Table 6:** Top canonical pathways affected by up-regulated genes/isoforms after thrombin treatment of HMVEC cells.

Up regulated genes		
Canonical Pathway	p value	Genes
Role of IL-17A in Psoriasis	0.000	23%
Differential Regulation of Cytokine Production in Macrophages and T Helper Cells by IL-17A and IL-17F	0.000	22%
TREM1 Signaling	0.000	19%
Role of Cytokines in Mediating Communication between Immune Cells	0.000	13%
Role of IL-17F in Allergic Inflammatory Airway Diseases	0.000	13%

Up regulated isoforms		
Canonical Pathway	p value	Genes
Differential Regulation of Cytokine Production in Macrophages and T Helper Cells by IL-17A and IL-17F	0.000	28%
TREM1 Signaling	0.000	23%
TNFR2 Signaling	0.000	21%
Lymphotoxin β Receptor Signaling	0.000	15%
Acute Myeloid Leukemia Signaling	0.000	13%
CD40 Signaling	0.000	13%

Top canonical pathways significantly up-regulated by thrombin treatment as determined by Ingenuity Pathway Analysis. Pathways with a p-value less than 0.05 defined as significant.

**Table 7:** Top canonical pathways affected by down-regulated genes/isoforms after thrombin treatment of HMVEC cells.

Down regulated genes		
Canonical Pathway	p value	Genes
DNA Double-Strand Break Repair by Homologous Recombination	0.000	64%
DNA Double-Strand Break Repair by Non-Homologous End Joining	0.000	57%
Role of BRCA1 in DNA Damage Response	0.000	36%
Aldosterone Signaling in Epithelial Cells	0.001	19%
Role of CHK Proteins in Cell Cycle Checkpoint Control	0.004	29%

Down regulated isoforms		
Canonical Pathway	p value	Genes
DNA Double-Strand Break Repair by Homologous Recombination	0.000	71%
DNA Double-Strand Break Repair by Non-Homologous End Joining	0.000	64%
Role of BRCA1 in DNA Damage Response	0.000	45%
Role of CHK Proteins in Cell Cycle Checkpoint Control	0.000	42%
RAN Signaling	0.011	39%

Top canonical pathways significantly down-regulated by thrombin treatment as determined by Ingenuity Pathway Analysis. Pathways with a p-value less than 0.05 defined as significant.

Canonical pathways analysis identified the pathways from the Ingenuity Pathways Analysis library of canonical pathways that were most significant to the data set. Genes with a 2-fold or greater change in expression after thrombin treatment and that were associated with a canonical pathway in Ingenuity's Knowledge Base were considered for the analysis. Top canonical pathways affected by upregulated genes/isoforms after thrombin treatment of HMVEC cells are listed in Table 6. Top canonical pathways affected by downregulated genes/isoforms after thrombin treatment of HMVEC cells are listed in Table 7. In concordance with the above observation that top affected networks by thrombin is inflammatory response, nearly all top canonical pathways affected by upregulated genes/isoforms after thrombin treatment are related to inflammatory response. IL17A is a proinflammatory cytokine produced by activated T cells. This cytokine regulates the activities of NF-kappaB and mitogen-activated protein kinases. This cytokine can stimulate the expression of IL6 and cyclooxygenase-2 (PTGS2/COX-2), as well as enhance the production of nitric oxide (NO). High levels of this cytokine are associated with several chronic inflammatory diseases including rheumatoid arthritis, psoriasis and multiple sclerosis [12]. TREM-1 is a positive regulator of inflammatory responses [13]. CD40 and CD40L are considered as neo-inflammatory molecules in vascular diseases [14]. In addition, Enhanced triggering receptor expressed on myeloid cells 1 (TREM1) Signaling may provide a new signal transduction pathway on the effect of Thrombin stimulated calcium influx into the cells since a novel TREM1 member, TREM-like transcript-1, can enhance calcium signaling via SHP-2 [15]. DNA Double-Strand Break Repair and BRCA1 in DNA Damage Response are top canonical pathways affected by down-regulated genes/isoforms after thrombin treatment of HMVEC cells (Table 7). Dysfunction of DNA repair pathways is among the major causes of tumorogenesis [16]. Mutated BRCA1 or 2, essential components of a repair pathway for repairing DNA double-strand breaks, underlie the pathogenesis of breast or ovarian cancers [16]. Thrombin is recognized as a potent mitogen in cancer and tumor metastasis [17]. The observation provided in Table 7 may shed a new light into the molecular mechanisms underlying thrombin in the tumor pathogenesis.

**Figure 4:**
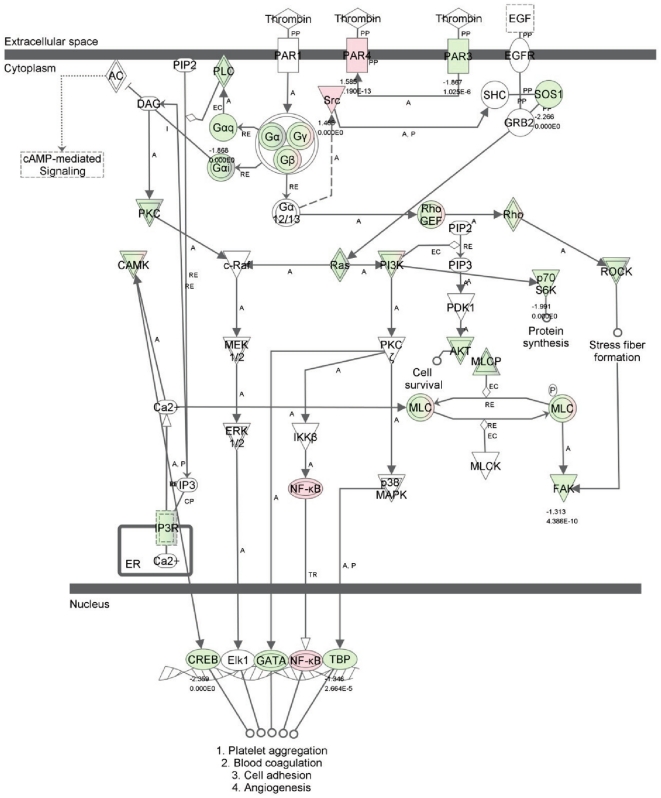
Display in the Thrombin Signaling pathway of those differentially expressed genes from thrombin treated HMVEC. Thrombin Signaling pathway was constructed by Ingenuity. Those with 1.3 fold up- or down-regulated genes in our data were uploaded and displayed into this pathway. Red color indicates up-regulation of gene expression and green down-regulated genes while white indicate no change of gene expression. The detailed fold changes and full name of each gene or isoform were presented in Table S4.

We also examined differentially expressed genes in traditional thrombin signaling pathway (Figure 4 and Table S4). Notably, 6 h thrombin treatment significantly upregulated thrombin receptor Par4 and down-regulated thrombin receptor Par 3 while there was no change in the expression of thrombin receptor Par 1. Expression of NFκB1, NF κB2 and Src was also significantly upregulated. Some of Rho family genes (Rho B, C, F and G) were upregulated while others (Rho J, Q, T1, U and V) were down regulated (Table S4). Myosin light chain gene 9 (MYL9) was upregulated while MYL12B was downregulated. Expression of other genes was either down-regulated or not affected.

### Detailed analysis of the differentially regulated protocadherin gene by Thrombin

**Figure 5:**
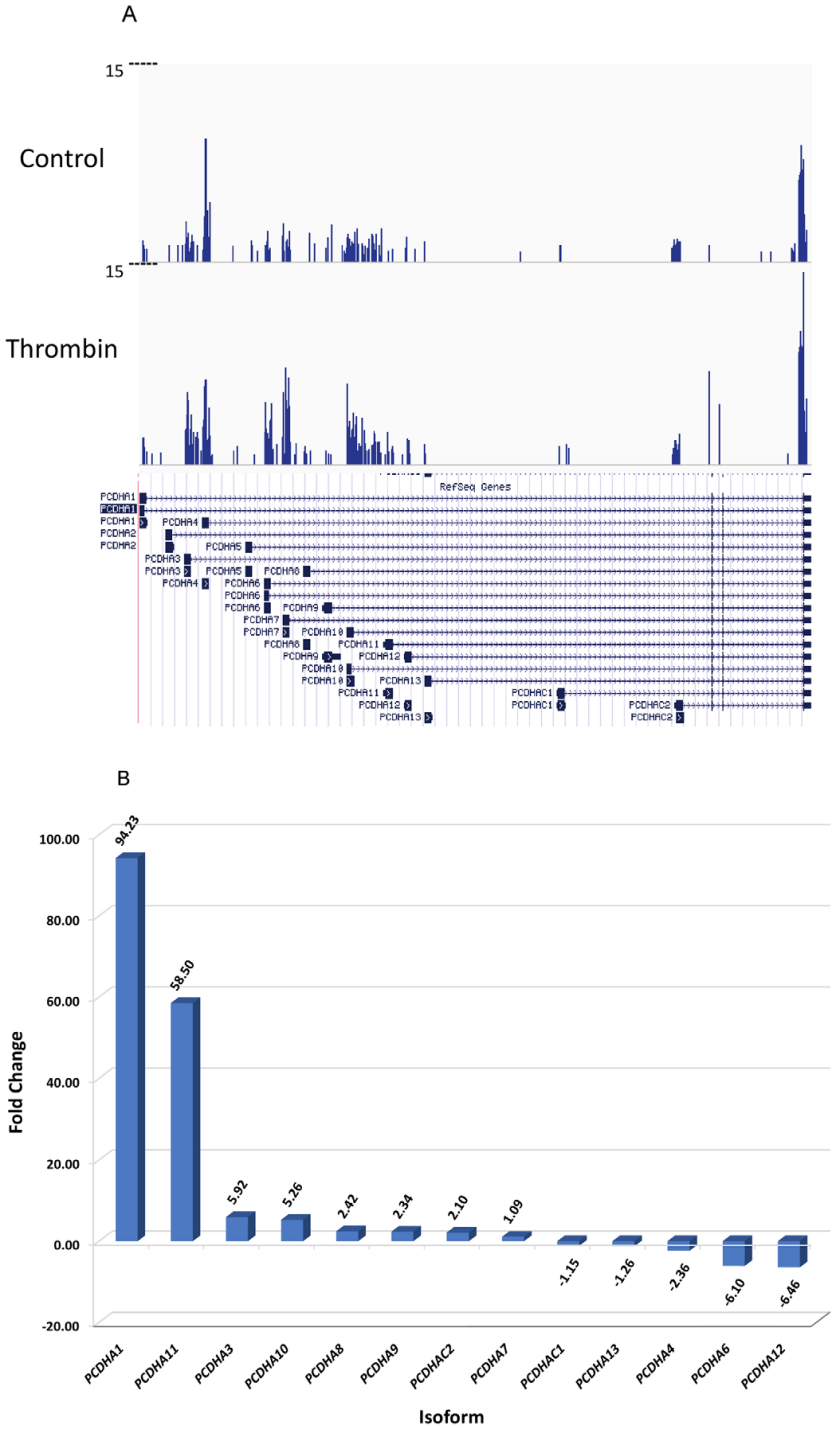
Protocadherin Alpha expression levels. **A.** **RNASeq read density aligned to the reference for** ***PCDHA*** **.** The RNASeq read density plotted along *PCDHA* is shown. Average alignment was computed by igvtools. Each bar represents the frequency of reads along the gene for the combined samples. **B.** **Histogram of Protocadherin Alpha fold changes.** A histogram showing the fold changes between thrombin-treated HMVEC cells and control cells, as calculated by CuffDiff. The fold change is the ratio of FPKM of *PCDHA* isoforms in thrombin treated HMVEC to FPKM of those in control cells.

Protocadherin gene is of particular interest due to its multiple isoforms, which varied from upregulation to downregulation after thrombin treatment of HMVEC cells. The mapping of reads for all protocadherin alpha isoforms except PCDHA2 and PCDHA5 , which are expressed in control cells but not thrombin-treated cells, to the reference genome shows differences in averaged expression levels for protocadherin alpha isoform exons between normal controls and thrombin-treated group (Fig. 5A). A histogram shows the fold changes between thrombin-treated HMVEC cells and control cells, as calculated by CuffDiff (Fig. 5B). Different protocadherin alpha isoforms from PCDHA1 to PCDHA13 except PCDHA2 and PCDHA5 responded to the thrombin treatment differently from the downregulation by −6.46 folds (PCDHA12) to the upregulation by 94.23 fold (PCDHA1). This result suggests that one PCDH isoform could function differently from another isoform. Gene isoforms are often produced by alternative splicing. The alternative spliced forms of PCDHA1, 11, 3, 10, 12, 6 were among the top ten up- and down- regulated genes showing alternative splicing in thrombin treated HMVEC cells (Table S5). Four protocadherin alpha family members, protocadherin alpha 10, 3, 11 and 1, were among the top 50 up- and down- regulated genes with alternative promoter usage in thrombin treated HMVEC cells (Table S6). Focusing on Protocadherin gene expression in the thrombin-treated and control groups illustrates that, used together, RNA-Seq and Cufflinks can identify not only transcriptional regulation of a gene but also post-transcriptional regulation of primary transcripts via alternative splicing.

### Validation of three differentially regulated genes by qRT-PCR

**Figure 6:**
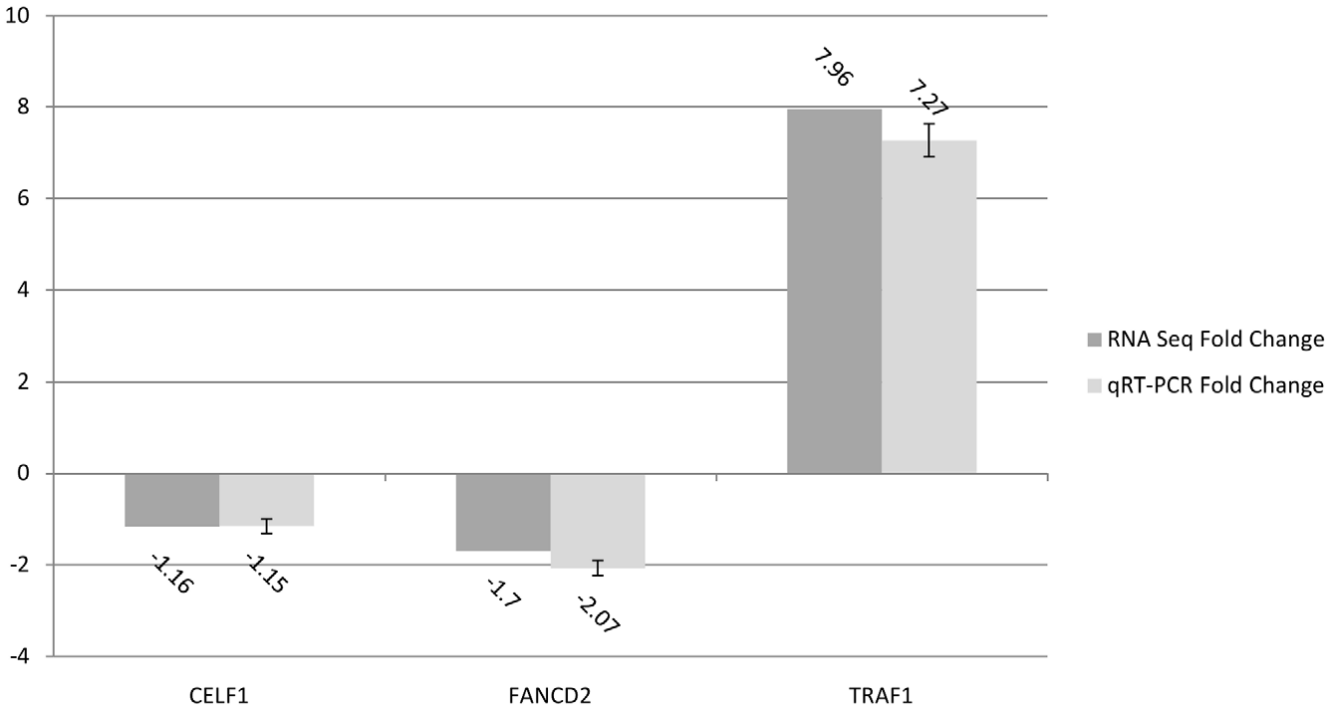
qRT-PCR validation of three differentially expressed genes from thrombin-treated HMVEC RNA-Seq data. qRT-PCR was carried out as described in the Method. Fold changes determined from the relative Ct values of TaqMan Gene Expression assay for CUGBP, Elav-like family member 1 (CELF1), Fanconi anemia, complementation group D2 (FANCD2) and TNF receptor-associated factor 1 (TRAF1) were compared to those detected by RNA-seq. Replicates (n = 4) of each sample were run and the Ct values averaged. All Ct values were normalized to beta-actin. The error bars represent the range of the fold change as determined by the Data Assist software.

To validate the RNA-seq results in this study using an alternative approach, we performed a qRT-PCR experiment to assay three different genes (Figure 6). In RNA-seq data, TNF receptor-associated factor 1 (TRAF1) was upregulated by 7.96 fold; CUGBP, Elav-like family member 1 (CELF1) was down-regulated by 1.16 fold; and Fanconi anemia, complementation group D2 (FANCD2) was down-regulated by 1.70 fold. In qRT-PCR data, these corresponding numbers are +7.25 fold, −1.15 fold and −2.07 fold, respectively. The results of these three genes assayed by RNA-seq and qRT-PCR are in good agreement, which corroborates our RNA-seq results.

## Discussion

Our study provides the first comprehensive insight into the transcriptome of human pulmonary microvascular endothelial cells treated with thrombin using RNA-seq, a powerful next-generation DNA sequencing platform, since a literature search for the PubMed database with the combination of key words of thrombin and RNA-seq retrieves zero records as of Sept. 16, 2011.

For RNA-seq, we used the Illumina HiScanSQ instrument to perform a 2×101 paired end run for all of our samples. The advantage of a paired end run is that both reads contain long range positional information, allowing for highly precise alignment of reads. We calculated the number of differentially expressed genes between thrombin treated cells and controls based on mean values of two duplicated samples in each group. We obtained a mean value of 102,568,132 reads per sample, which is more than enough to deliver sufficient sequence coverage for transcriptome profiling according to a previous report by Sultan et al. [18]. Our mean rate of 91.3% total reads that map to the reference genome met quality standards of the RNA-seq technique [19]. A breadth of the RNA sequencing reads covering chromosome 1 indicates quality RNA-seq coverage (Fig. 1). Hence, we are confident that our mRNA-Seq data provide a good quality and objective profile of transcriptome in human pulmonary microvascular endothelial cells treated with thrombin. In addition, a qRT-PCR experiment (Figure 6) on three randomly selected genes (TRAF1, CELF1, FANCD2) confirmed our results from RNA-seq. This adds more weight to the credibility of our RNA-seq data in this study.

Although thrombin is known for its central role in the coagulation cascade acting as both a coagulant factor and an anticoagulant factor, thrombin can trigger important cellular effects via a protease-activated receptor pathway or receptor independent pathways [2]. The list of physiological and pathological roles of thrombin is expanding. In the present study, we performed a comprehensive transcriptome analysis in human pulmonary microvascular endothelial cells (HMVEC-L) treated with or without thrombin using the Illumina RNA-seq technique. The aim of this study is to provide a comprehensive gene expression profile of HMVEC-L treated with thrombin to gain a complete picture of the transcriptional regulation of vascular endothelial cells by thrombin since the dysregulation of vascular endothelial cells by different agonists including thrombin has been implicated in the development of a number of pathologic disorders, such as inflammatory conditions, cancer, diabetes and coronary heart diseases. Thrombin plays an important role in vascular inflammation [11]. However, the full repertoire of players and signal transduction pathways of this process are not completely understood. We found that TNF receptor-associated factor 1 and chemokine C-X3-C motif ligand 1 (C-X3-CL1), both of which are involved in inflammatory response, were among the top ten upregulated genes by thrombin (Figure 2). Inflammatory response is among the top networks and canonical pathways of both upregulated genes and isoforms by the thrombin treatment (Table 4 and 6). These results corroborate the role of thrombin in vascular inflammation, but more importantly, they have provided a complete list of differentially expressed genes after thrombin treatment, as well as, full components in the top affected network and canonical pathway of inflammatory response. By examining differentially expressed genes in a traditional thrombin signaling pathway (Figure 4), we found that thrombin receptor, Par 4 not Par 1, was up-regulated at the 6 h thrombin treatment. This makes sense since Par 1 is involved in an early response and Par 4 is involved in an later response of Thrombin. PARs contribute to the proinflammatory phenotype observed in endothelial dysfunction [20]. Along the same line, expression of NFκB was significantly up-regulated at the 6 h thrombin treatment. NF-κB has been documented as the key regulator of inflammation [21]. The up-regulated Par4- and NF-κB-mediated effects may in part contribute to the thrombin mediated inflammatory response as a major affected transcriptional network or pathway in this study although other genes in this traditionally annotated thrombin pathway were either down-regulated or not affected (Table S4). Several protocadherin alpha members such as PCDHA1, PCDHA3 and PCDHA11 were among top upregulated genes by thrombin (Figure 2 and Table S2). Figure 5 displays a full spectrum of differential regulations of protocadherin alpha 1 to 13 by thrombin. Protocadherins are an important subfamily of the cadherin superfamily proteins. They are calcium-dependent cell adhesion molecules. They are also involved in many biological processes, including cell recognition and cell signaling [22]. Network analysis of the top differentially regulated genes indicate that Cell-To-Cell Signaling and Interaction, Tissue Morphology, and Cellular Movement are among the top affected networks by the thrombin treatment (Table 4 and 5). These observations provide a transcriptional level support and insight into known roles of thrombin that can regulate cell-cell junctions between endothelial cells as well as cytoskeleton contraction. Thrombin is also recognized as a potent mitogen in cancer and tumor metastasis [17]. We found that DNA Double-Strand Break Repair and BRCA1 in DNA Damage Response were the top canonical pathways of downregulated genes/isoforms after thrombin treatment of HMVEC cells (Table 9). Dysfunction of the DNA repair pathway is among the major causes of tumorogenesis [16]. Our observation may provide new molecular mechanisms underlying thrombin in the tumor pathogenesis.

Previously, DNA microarray analysis has been performed to profile gene expression pattern in human vascular endothelial cells treated with thrombin. By comparing the RNA-seq data from this study to those by Uzonyi et al. [23] in which gene expression profile of 6 h thrombin treated HUVEC was assayed by Affymetrix Human Genome U133A Array, we found that 38 genes were consistently up-regulated and 395 genes were consistently down-regulated in both datasets (Table S7). Many of the changes we observed in gene expression between control and thrombin group were similar to those reported. For example, the upregulation trend of several inflammatory response genes (CX3CL1, 6.05 fold; VCAM1, 4.75 fold; ICAM1, 3.29 fold; IL6, 2.27 fold; NFκB2, 2.02 fold) in thrombin treated HMVEC-L cells revealed by RNA-seq in this study is the same as that detected using DNA microarray array (CX3CL1, 1.39 fold; VCAM1, 4.16 fold; ICAM1, 3.14 fold; IL6, 1.34 fold; NFκB2, 1.41 fold) by Uzonyi et al. [23]. Generally, RNA-seq is much more sensitive than DNA microarray to detect the magnitude of difference in differentially expressed genes after the thrombin treatment. Some highly expressed genes or isoforms such as AS3MT (6.96 fold ↑) and PCDHA11 (58.5 fold ↑) as well as lowly expressed genes and isoforms in this study were not detected in DNA microarray [23]. Although the slightly different cell type and old DNA microarray chips may be contributing factors to some of the disconcordance between our RNA-seq transcriptome data set and the result of DNA microarray, these differences most likely stem from inherent limitations in microarray systems. We also compared the data from this study to those by Viemann et al. [24] in which gene expression profile of 5 h TNFα treated HMVEC was assayed by Affymetrix Human Genome U133A Array. We found that 49 genes were consistently up-regulated and 264 genes were consistently down-regulated in both datasets (Table S8). TNFα plays an important pathogenetic role in inflammatory disorders [25]. Several upregulated genes known to be invovled in the inflammatory response such as NFκB2, VCAM1, ICAM1, IL6, IL8, TNFRSF9 were similarly detected in 5 h TNFα treated HMVEC and 6 h thrombin treated HMVEC in our study though the reported magnitude of increased expressions were different. These cross comparisons between our RNA-seq data and those of DNA microarray analysis of either 6 h thrombin treated HUVEC or 5 h TNFα treated HMVEC have provided a significant overlapping list of differentially expressed genes, supporting the robust utility of our dataset. One notable advantage of RNA-seq is that it can measure the less abundant transcripts that are inaccurate or not detected by microarray. RNA-seq also enabled the detection of unknown RNAs, alternative spliced transcripts and alternative promoter usages which are not feasible by microarray [6], [26]. Indeed, our RNA-Seq study reveals nearly 1/3 of the differentially expressed RNA transcripts are unannotated, while about 26,000 novel isoforms are detected. Among those novel isoforms, 418 were only detected in the control cells, while 424 were only detected in thrombin treated cells. The differentially expressed novel isoforms in thrombin treated cells over the controls with statistically significant fold changes ranged from −927.74 to 431.33. There are 1,775 upregulated- and 12,202 downregulated- novel isoforms in thrombin treated cells by at least a 2 fold change over the control cells (Table 2). This study has provided rich reagents for further experimentation.

It should be pointed out that one limitation of our study is the one time point, 6 h, of the thrombin treatment. The reason we selected 6 h of thrombin treatment was based on at least the following three references: 1). Simoncini et al. showed that thrombin mediated upregulation of T*NFSF10* (*TRAIL*), a TNF-α superfamily inflammatory cytokine that displays multiple effects on endothelial cells, started to peak at 6 h time point in both human microvascular endothelial cells and HUVEC as quantified by real-time PCR [27]. 2). Moser et al. showed that a 6 h thrombin treatment significantly enhanced the adhesive interaction of neutrophils to HUVEC as IL-1, TNF and LPS did compared to those of the 5 min thrombin treatment [28]. A transendothelial neutrophil passage is one of hallmark features in the inflammatory process. 3) Liu et al. employed a 6 h thrombin treatment to investigate thrombin mediated myocyte-specific enhancer factor-2 (MEF2)-dependent gene transcription of endothelial cells in angiogenesis [29]. Our data from this study have provided a valuable global picture on the thrombin mediated up-regulation and down-regulation of multiple genes in the endothelium at the 6 h thrombin treatment as discussed above. Nevertheless, previous studies have showed that of the genes that were induced by thrombin, some peaked at 1 hour, some at 4 hours, and some at 18 hours [30]. Our study with 6 h thrombin treatment failed to detect early and late thrombin response genes. It is certainly warranted that a series of time course experiments of thrombin treatment should be carried out to systematically survey the transcriptional regulation of thrombin on endothelial cells to provide more complete and dynamic datasets using the new and powerful RNA-seq technology. A better understanding of the temporal dynamics of these thrombin regulated transcriptional networks may help to decipher the link between thrombin signaling in the endothelium and multiple phenotypic changes.

In summary, our first complete transcriptome analysis of human pulmonary microvascular endothelial cells treated with thrombin using the RNA-seq has provided important insights into the transcriptional regulation of gene expression in HMVEC-L cells by thrombin. Further in-depth follow-up analysis of the transcriptional regulation reported in this study may shed light on molecular mechanisms underlying thrombin mediated endothelial dysfunction in the pathogenesis of inflammatory conditions, cancer, diabetes, coronary heart disease and provide new leads of therapeutic targets to those diseases.

## Materials and Methods

### Cell culture and RNA isolation

Human Lung Microvascular Endothelial Cells (HMVEC, Lonza catalog # CC-2815) were cultured as described previously [31]. Briefly, cells were grown to 90–100% confluence on the 7^th^ passage in T-75 flasks in EGM-2 medium with 5% FBS, growth factors and antibiotics (Lonza, Bullet Kit catalog # CC-3202). Media was changed to a starvation media (0% FBS) 30 minutes prior to treatment. The cells were treated with 0.05 U/ml thrombin (Sigma-Aldrich, catalog # T4393, Lot 087K7575) or left untreated for 6 hours at 37°C. Total RNA was isolated from the treated and control cells using the MirVana kit (Ambion, catalog # AM1560) according to manufacturer's instructions.

### Library preparation and sequencing

Concentration of the RNA samples was determined using the Quant-iT RiboGreen Kit (Invitrogen catalog # R11490) according to manufacturer's instructions. Each sample was assayed 3 times and the results averaged. The quality of the RNA was checked using the RNA Std Sense Chip on the Bio-Rad Experion system (catalog # 700-7103). The RNA was prepared for sequencing using the TruSeq RNA Sample Prep Kit, Set A (Illumina catalog # FC-122-1001) according to manufacturer's instructions. Each sample was prepared in duplicate and 2 µg total RNA was used. The resulting cDNA libraries were quantified using the Epoch system and checked for quality and size using the Experion DNA 1K chip (catalog # 700-7107). A portion of each library was diluted to 10 nM and stored at −20°C. 2 µL of the 10 nM libraries were diluted and denatured, according to instructions from Illumina. The resulting cDNA was diluted to 5 pM and 1% PhiX control (Illumina catalog # FC-110-3001) was spiked in each sample. A 6 pM dilution of PhiX Control was also prepared. The samples were loaded on to the cBot (Illumina) for clustering on a flow cell according to manufacturer's instructions.

The flow cell was then sequenced using a HiScanSQ (Illumina) according to manufacturer's instructions. A paired-end (2×101) run was performed using the SBS Kit (Illumina catalog # FC-401-1001). Real-time analysis and basecalling was performed using the HiSeq Control Software Version 1.3.8 (Illumina).

### Primary processing of Illumina RNA-Seq reads

The number of clusters detected,percentage of those clusters passing the software's internal filter and the alignment to the PhiX genome were all within acceptable limits. The resulting .bcl files were converted first to .qseq files using Illumina's Off-Line Basecaller (OLB) and the .qseq files were subsequently converted to FASTQ files. The sequence data have been submitted to the NCBI Short Read Archive with accession number SRA046349.2.

### Mapping of RNA-Seq reads and transcript assembly and abundance estimation using Tuxedo Suite

The sequences were aligned to the UCSC *Homo sapiens* reference genome hg19 using TopHat v1.3.0 [32], which is integrated with Bowtie v0.12.7 [33]. TopHat removes a small number of reads based on quality scoring accompanying each read and then maps the reads to a provided reference genome. The pre-built UCSC *H. sapiens* hg19 bowtie index was downloaded from the Illumina FTP site (ftp://ftp.illumina.com/Homo_sapiens/UCSC/hg19/Homo_sapiens_UCSC_hg19.tar.gz). TopHat's default settings were used: 40 alignments per read were allowed, with up to 2 mismatches per alignment. Each contiguously-mapping read is considered a “coverage island,” which are often spliced together with neighboring “islands.” These “islands” can be considered probable exons. TopHat uses this initial mapping to build a database of potential splice junctions and verifies them by comparing the previously unmapped reads against the junction database.

The resulting aligned reads were analyzed further by Cufflinks v1.0.3 [34] in several ways. Cufflinks assembled the aligned reads into transcripts, either with or without a reference genome and reported the expression of those transcripts in *Fragments Per Kilobase of exon per Million fragments mapped* (FPKM). FPKM is an expression of the relative abundance of transcripts. To determine differential expression of known isoforms between the treatment and control groups, Cuffdiff analyses were performed using the reference genome comparing the thrombin treatment samples to the control samples. The biologic replicates were pooled for those analyses. To detect novel isoforms, Cufflinks was run without a reference genome. This method allows the construction of the minimum number of transcripts that described the data without a bias towards already discovered transcripts. The sample transcript files were then compared to the reference genome using Cuffcompare, effectively filtering out those already-discovered transcripts. To test the differential expression of these novel isoforms, Cuffdiff analyses were performed using the combined transcript files as the reference genome. Cuffdiff analyses were performed two ways: comparing the thrombin transcripts to the control transcripts, using the thrombin transcripts as the reference genome; and comparing the thrombin transcripts to the control transcripts, using the control transcripts as the reference genome.

### Visualization of mapped reads

Aligned reads were visualized using a local copy of the Integrative Genomics Viewer (www.broadinstitute.org/igv/). The output files generated from TopHat were converted into files viewable in IGV by BEDTools [35] and then further processed by the “count” function in igvtools (included with the IGV software) to create an average alignment track viewable as a bar chart. The log_2_ of the frequency of the reads was plotted to better visualize the extensive range of the read coverage. Individual gene views were created by first merging the TopHat output files from the control and thrombin-treatment samples into two files using SAMTools [36]. These merged files were processed in the same way as above with the “count” function in igvtools. The raw frequency of the reads was visualized in this case.

### Functional analysis of differentially expressed gene lists using Ingenuity Pathway Analysis

The differentially expressed gene lists were submitted to Ingenuity Pathway Analysis (IPA) v9.0-3211 (Ingenuity Systems, Inc., Redwood City, CA). Genes with a 2-fold or greater change in expression after thrombin treatment were uploaded to the application. Each identifier was mapped to its corresponding object in Ingenuity's Knowledge Base. These molecules, called Network Eligible molecules, were overlaid onto a global molecular network developed from information contained in Ingenuity's Knowledge Base. Networks of Network Eligible Molecules were then algorithmically generated based on their connectivity. The following settings were used: Ingenuity Knowledge Base; Endogenous Chemicals not included; Direct and Indirect relationships; molecules per pathway: 70; and networks per analysis: 25.

Canonical pathways analysis identified the pathways from the Ingenuity Pathways Analysis library of canonical pathways that were most significant to the data set. Genes with a 2-fold or greater change in expression after thrombin treatment and that were associated with a canonical pathway in Ingenuity's Knowledge Base were considered for the analysis. The significance of the association between the data set and the canonical pathway was measured in 2 ways: 1) the ratio of the number of molecules from the data set that map to the pathway divided by the total number of molecules that map to the canonical pathway, and 2) Fisher's exact test was used to calculate a p-value determining the probability that the association between the genes in the dataset and the canonical pathway is explained by chance alone.

### Computer hardware specifications

TopHat, Bowtie and Cufflinks were installed and run on a CentOS 5.5 Advanced Clustering Pinnacle 1BX5501 machine with 20 nodes, totaling 240 cores and 960 GB RAM.

### qRT-PCR Validation of RNA Seq Results

The following genes were selected for validation and TaqMan Gene Expression Assays were ordered from Applied Biosystems (cat. #4331182): CELF1 (Hs00198069_m1), FANDCD2 (Hs00276992_m1), TRAF1 (Hs01090170_m1), and ACTB, as a control (Hs99999903_m1). All genes were significantly differentially expressed in our RNA Seq data as determined by Cufflinks. cDNA was made from RNA previously isolated from control HMVEC and thrombin-treated HMVEC. Gene expression assays were run according to manufacturer's instructions (www.appliedbiosystems.com) on a Viia7 qPCR instrument. Replicates (n = 4) of each sample were run. The results were loaded into a local copy of DataAssist (www.appliedbiosystems.com/dataassist) and normalized to ACTB Ct values. The fold change and range of the fold change was calculated for each gene.

### Statistical Analysis

Besides those statistical tools embedded in Illumina HiScanSQ instrument or other softwares, additional statistical analyses were performed using Sigmaplot, a scientific data analysis and graphing software (ver 12, Systat Software, Inc., San Jose, CA). Thrombin treated groups were compared with the control groups by unpaired Student's *t* test. *p*<0.05 was considered statistically significant.

## Supporting Information

Table S1 **Top 50 up- and down-regulated genes in thrombin treated HMVEC cells.** Significantly differentially expressed genes were determined by CuffDiff, after Benjamini-Hochberg correction. The fold change is the ratio of thrombin FPKM to control FPKM. The genes were ranked on their fold change and the 50 with the highest or lowest fold changes are listed here.
(DOCX)

Table S2 **Top 50 up- and down-regulated known isoforms in thrombin treated HMVEC cells.** Significantly differentially expressed isoforms were determined by CuffDiff, after Benjamini-Hochberg correction. The fold change is the ratio of thrombin FPKM to control FPKM. The isoforms were ranked on their fold change and the 50 with the highest or lowest fold changes are listed here.
(DOCX)

Table S3 **Top 50 up- and down-regulated novel isoforms in thrombin treated HMVEC cells.** Significantly differentially expressed novel isoforms were determined by CuffDiff, after Benjamini-Hochberg correction. The fold change is the ratio of thrombin FPKM to control FPKM. The novel isoforms were ranked on their fold change and the 50 with the highest or lowest fold changes are listed here.
(DOCX)

Table S4 Differentially expressed genes and isoforms in Thrombin Signaling Pathway* * Fold change was calculated by *Fragments Per Kilobase of exon per Million fragments mapped* (FPKM) in Thrombin treated group dividing FPKM in control group.
(DOCX)

Table S5 **Top ten up- and down- regulated genes showing alternative splicing in thrombin treated HMVEC Cells.** The top 10 up- and down-regulated genes that exhibit significant alternative splicing according to CuffDiff were presented.
(DOCX)

Table S6 **Top 50 up- and down-regulated genes with alternative promoter usage in thrombin treated HMVEC cells.** The 50 top up- and down- regulated genes after thrombin treatment with any significant alternative promoter usage were determined by CuffDiff.
(DOCX)

Table S7 RNA Seq Data from 6 h thrombin-treated HMVEC vs Microarray Data from 6 h thrombin-treated HUVEC * * The data from this study is compared to the study by Uzonyi et al. (23) in which gene expression profile was assayed by Affymetrix Human Genome U133A Array.
(DOCX)

Table S8 RNA Seq Data from 6 h thrombin-treated HMVEC vs Microarray Data from 5 h TNFα-treated HMVEC*. * The data from this study is compared to the study by Viemann et al.(24) in which gene expression profile was assayed by Affymetrix Human Genome U133A Array. ** ND, not detected either in control group or TNF alpha treated group.
(DOCX)
