# Supplementary material for: RNA-seq Reveals Novel Transcriptome of Genes and Their Isoforms in Human Pulmonary Microvascular Endothelial Cells Treated with Thrombin
Source: PLoS One. 2012 Feb 16;7(2):e31229. doi: 10.1371/journal.pone.0031229 (PMC3281071; doi:10.1371/journal.pone.0031229)
Supplement: Table S1 — Top 50 up- and down-regulated genes in thrombin treated HMVEC cells. Significantly differentially expressed genes were determined by CuffDiff, after Benjamini-Hochberg correction. The fold change is the ratio of thrombin FPKM to control FPKM. The genes were ranked on their fold change and the 50 with the highest or lowest fold changes are listed here. (DOCX) [file pone.0031229.s001.docx]

| Table S1: Top 50 up- and down-regulated genes in thrombin treated HMVEC cells | | | | | |  |
| --- | --- | --- | --- | --- | --- | --- |
|  |  |  |  |  |  |  |
| Gene | Chr. | FPKM Control | FPKM Thrombin | Fold Change | p_value | significant (after FDR) |
| EGR1 | chr5 | 1.12887 | 3.75772 | 3.33 | 0 | yes |
| TGIF2-C20ORF24 | chr20 | 0.28355 | 0.94874 | 3.35 | 0.00135129 | yes |
| ICOSLG | chr21 | 8.75123 | 29.4661 | 3.37 | 0 | yes |
| JAG1 | chr20 | 31.2329 | 105.584 | 3.38 | 0 | yes |
| KCNF1 | chr2 | 0.0816244 | 0.276473 | 3.39 | 0.00825101 | yes |
| CXCR7 | chr2 | 14.5883 | 50.0941 | 3.43 | 0 | yes |
| PRR16 | chr5 | 1.30524 | 4.51293 | 3.46 | 0 | yes |
| SNORD10 | chr17 | 2.27377 | 7.91711 | 3.48 | 0.0210728 | yes |
| NOD2 | chr16 | 0.0836249 | 0.300964 | 3.60 | 5.62E-05 | yes |
| TMEM158 | chr3 | 4.82316 | 17.5471 | 3.64 | 0 | yes |
| SEMA7A | chr15 | 1.09092 | 3.97069 | 3.64 | 2.48E-05 | yes |
| CLDN14 | chr21 | 13.8935 | 50.5847 | 3.64 | 0 | yes |
| TMCC2 | chr1 | 2.38247 | 8.7945 | 3.69 | 0 | yes |
| EPPK1 | chr8 | 0.0147159 | 0.0557023 | 3.79 | 0.0211654 | yes |
| SP6 | chr17 | 0.247214 | 0.954782 | 3.86 | 1.56E-11 | yes |
| CD83 | chr6 | 0.613352 | 2.40498 | 3.92 | 6.58E-09 | yes |
| CA2 | chr8 | 0.318269 | 1.28262 | 4.03 | 3.49E-07 | yes |
| CYGB | chr17 | 0.126121 | 0.515731 | 4.09 | 0.000399953 | yes |
| IL34 | chr16 | 0.920665 | 3.79619 | 4.12 | 6.66E-08 | yes |
| C2CD4A | chr15 | 0.204962 | 0.845954 | 4.13 | 7.10E-10 | yes |
| C1QTNF1 | chr17 | 1.98325 | 8.2049 | 4.14 | 1.31E-13 | yes |
| NR4A3 | chr9 | 0.378358 | 1.60254 | 4.24 | 2.53E-06 | yes |
| KCNN2 | chr5 | 5.19455 | 22.0577 | 4.25 | 0 | yes |
| IL1B | chr2 | 2.08867 | 8.88695 | 4.25 | 0 | yes |
| KIAA1644 | chr22 | 0.164649 | 0.731178 | 4.44 | 2.22E-16 | yes |
| RND1 | chr12 | 11.3509 | 51.0221 | 4.49 | 0 | yes |
| VCAM1 | chr1 | 14.1134 | 67.0499 | 4.75 | 0 | yes |
| NCF4 | chr22 | 0.175215 | 0.847413 | 4.84 | 0.000965275 | yes |
| ADAMTS4 | chr1 | 6.61854 | 32.0479 | 4.84 | 0 | yes |
| BDKRB2 | chr14 | 0.734743 | 3.6247 | 4.93 | 0 | yes |
| TP63 | chr3 | 0.0251943 | 0.12449 | 4.94 | 0.00640843 | yes |
| MRGPRX3 | chr11 | 0.108197 | 0.5412 | 5.00 | 0.000535119 | yes |
| SELE | chr1 | 104.223 | 534.876 | 5.13 | 2.22E-16 | yes |
| PCDHA10 | chr5 | 0.0334417 | 0.175998 | 5.26 | 0.00343252 | yes |
| LIF | chr22 | 0.573387 | 3.16276 | 5.52 | 0 | yes |
| CSF2 | chr5 | 5.47285 | 30.3124 | 5.54 | 0 | yes |
| METTL7B | chr12 | 0.00148495 | 0.00845594 | 5.69 | 0.000356285 | yes |
| TLR2 | chr4 | 0.491175 | 2.80697 | 5.71 | 0 | yes |
| PCDHA3 | chr5 | 0.0110876 | 0.0656043 | 5.92 | 0.018882 | yes |
| CCL20 | chr2 | 2.33358 | 13.8851 | 5.95 | 0 | yes |
| CX3CL1 | chr16 | 8.57229 | 51.8334 | 6.05 | 0 | yes |
| NR4A1 | chr12 | 0.518019 | 3.16505 | 6.11 | 0 | yes |
| HUS1B | chr6 | 0.0277758 | 0.186468 | 6.71 | 4.18E-06 | yes |
| LOC441617 | chr11 | 0.08781 | 0.597294 | 6.80 | 3.77E-07 | yes |
| AS3MT | chr10 | 0.0976933 | 0.673839 | 6.90 | 4.49E-07 | yes |
| RMRP | chr9 | 4.19652 | 30.02 | 7.15 | 1.52E-12 | yes |
| TRAF1 | chr9 | 9.59799 | 76.4107 | 7.96 | 0 | yes |
| RPPH1 | chr14 | 1.45004 | 18.7436 | 12.93 | 5.38E-13 | yes |
| PCDHA11 | chr5 | 0.000876634 | 0.0512832 | 58.50 | 0 | yes |
| PCDHA1 | chr5 | 0.00187956 | 0.177114 | 94.23 | 0 | yes |
| GHRLOS | chr3 | 0.803081 | 0.0106653 | -75.30 | 0 | yes |
| IGSF10 | chr3 | 0.247018 | 0.00494311 | -49.97 | 1.12E-05 | yes |
| ACRV1 | chr11 | 2.39544 | 0.0514809 | -46.53 | 2.48E-07 | yes |
| LOC728066 | chr7 | 0.46925 | 0.0144953 | -32.37 | 0 | yes |
| LOC100132215 | chr2 | 0.241372 | 0.0108699 | -22.21 | 0 | yes |
| CCDC144C | chr17 | 0.153752 | 0.00714246 | -21.53 | 0.00207889 | yes |
| C2orf66 | chr2 | 0.439745 | 0.0224186 | -19.62 | 0.00374361 | yes |
| MLANA | chr9 | 0.384067 | 0.0202823 | -18.94 | 0.00420134 | yes |
| TEX15 | chr8 | 0.0492919 | 0.00271593 | -18.15 | 0.00484801 | yes |
| CD37 | chr19 | 1.37147 | 0.0840455 | -16.32 | 2.03E-06 | yes |
| DNAH8 | chr6 | 0.273287 | 0.0172169 | -15.87 | 8.88E-16 | yes |
| GALNT3 | chr2 | 0.256089 | 0.0167035 | -15.33 | 0.000268044 | yes |
| KLRAP1 | chr12 | 0.899625 | 0.0591697 | -15.20 | 3.38E-08 | yes |
| CCDC39 | chr3 | 0.583786 | 0.0395993 | -14.74 | 1.97E-09 | yes |
| LOC338588 | chr10 | 0.242386 | 0.0166452 | -14.56 | 1.48E-12 | yes |
| C10orf62 | chr10 | 0.0293376 | 0.00201723 | -14.54 | 3.48E-06 | yes |
| LOC221442 | chr6 | 0.337567 | 0.0232956 | -14.49 | 5.93E-07 | yes |
| WDR52 | chr3 | 0.720711 | 0.0523623 | -13.76 | 5.01E-13 | yes |
| CCDC144A | chr17 | 0.200955 | 0.0147992 | -13.58 | 1.12E-05 | yes |
| SLC24A5 | chr15 | 0.227448 | 0.0168103 | -13.53 | 0 | yes |
| TSIX | chrX | 0.0164606 | 0.0012303 | -13.38 | 0.00132455 | yes |
| FMR1-AS1 | chrX | 0.0178352 | 0.00137542 | -12.97 | 0.0015516 | yes |
| LRRC39 | chr1 | 0.40751 | 0.031918 | -12.77 | 0.0034176 | yes |
| GAS2 | chr11 | 0.664231 | 0.0547135 | -12.14 | 4.31E-05 | yes |
| SESN3 | chr11 | 2.9319 | 0.247464 | -11.85 | 0 | yes |
| KCNJ13 | chr2 | 0.0490869 | 0.00414677 | -11.84 | 2.90E-09 | yes |
| CCDC152 | chr5 | 0.0249545 | 0.00213549 | -11.69 | 0.000207315 | yes |
| GGT8P | chr2 | 0.136232 | 0.0117657 | -11.58 | 0.0190699 | yes |
| ZNF117 | chr7 | 24.3404 | 2.11915 | -11.49 | 0 | yes |
| HSD17B3 | chr9 | 0.314786 | 0.0281554 | -11.18 | 0.0210652 | yes |
| CCDC144B | chr17 | 1.2229 | 0.109383 | -11.18 | 0 | yes |
| IMPG1 | chr6 | 0.149048 | 0.0135085 | -11.03 | 0.00453303 | yes |
| LOC220594 | chr17 | 2.18882 | 0.203826 | -10.74 | 0 | yes |
| LOC162632 | chr17 | 0.322667 | 0.0300587 | -10.73 | 4.69E-06 | yes |
| CCDC146 | chr7 | 0.359483 | 0.0339768 | -10.58 | 8.38E-06 | yes |
| LOC100130872,SPON2 | chr4 | 0.0138 | 0.0013147 | -10.50 | 0.00286382 | yes |
| AMY2B | chr1 | 0.34234 | 0.0327752 | -10.45 | 0.000520899 | yes |
| CG030 | chr13 | 0.293246 | 0.0288359 | -10.17 | 8.66E-07 | yes |
| FAM106A | chr17 | 0.482293 | 0.0477313 | -10.10 | 2.43E-05 | yes |
| PLCB4 | chr20 | 0.682668 | 0.0679837 | -10.04 | 6.28E-14 | yes |
| GOLGA8IP | chr15 | 0.233891 | 0.0237295 | -9.86 | 0.00773685 | yes |
| VIP | chr6 | 1.90712 | 0.193692 | -9.85 | 1.48E-09 | yes |
| SLC25A27 | chr6 | 0.19137 | 0.019598 | -9.76 | 0.00220255 | yes |
| C8orf77 | chr8 | 0.0464968 | 0.00480485 | -9.68 | 5.48E-07 | yes |
| ABCA9 | chr17 | 1.93901 | 0.201577 | -9.62 | 0 | yes |
| LOC643596 | chr9 | 0.292916 | 0.0307195 | -9.54 | 0.00879121 | yes |
| LRGUK | chr7 | 0.136928 | 0.014361 | -9.53 | 0.0135048 | yes |
| CENPE | chr4 | 6.0138 | 0.631452 | -9.52 | 0 | yes |
| NOXA1 | chr9 | 0.182541 | 0.0191712 | -9.52 | 2.88E-06 | yes |
| RBM44 | chr2 | 0.105333 | 0.0111113 | -9.48 | 0.00587553 | yes |
|  |  |  |  |  |  |  |
|  | | | | | | |
|  |  |  |  |  |  |  |
|  |  |  |  |  |  |  |
