# Supplementary material for: RNA-seq Reveals Novel Transcriptome of Genes and Their Isoforms in Human Pulmonary Microvascular Endothelial Cells Treated with Thrombin
Source: PLoS One. 2012 Feb 16;7(2):e31229. doi: 10.1371/journal.pone.0031229 (PMC3281071; doi:10.1371/journal.pone.0031229)
Supplement: Table S2 — Top 50 up- and down-regulated known isoforms in thrombin treated HMVEC cells. Significantly differentially expressed isoforms were determined by CuffDiff, after Benjamini-Hochberg correction. The fold change is the ratio of thrombin FPKM to control FPKM. The isoforms were ranked on their fold change and the 50 with the highest or lowest fold changes are listed here. (DOCX) [file pone.0031229.s002.docx]

| Table S2: Top 50 up- and down-regulated known isoforms in thrombin treated HMVEC cells | | | | | |  |
| --- | --- | --- | --- | --- | --- | --- |
|  |  |  |  |  |  |  |
| Gene | Chr. | FPKM Control | FPKM Thrombin | Fold Change | p_value | significant (after FDR) |
| HFE | chr6 | 0.00345846 | 3.32901 | 962.57 | 0.000663859 | yes |
| DMD | chrX | 0.000131431 | 0.0902055 | 686.34 | 0 | yes |
| RNF182 | chr6 | 0.000283666 | 0.0668129 | 235.53 | 0 | yes |
| NEK6 | chr9 | 0.0509293 | 10.6539 | 209.19 | 0 | yes |
| IL34 | chr16 | 0.00576276 | 1.197 | 207.71 | 0.000178136 | yes |
| SEMA4A | chr1 | 0.000408987 | 0.0438428 | 107.20 | 0 | yes |
| RUNX1T1 | chr8 | 0.000128507 | 0.013434 | 104.54 | 0.0108442 | yes |
| FOXP2 | chr7 | 8.54E-05 | 0.00839985 | 98.37 | 2.41E-09 | yes |
| SCN4B | chr11 | 0.000131959 | 0.0120634 | 91.42 | 6.36E-06 | yes |
| PCDHGB6 | chr5 | 0.00673875 | 0.549669 | 81.57 | 0 | yes |
| SCN4B | chr11 | 0.000181875 | 0.0132081 | 72.62 | 7.06E-07 | yes |
| GAS7 | chr17 | 2.36E-05 | 0.00169093 | 71.57 | 2.42E-05 | yes |
| PODXL | chr7 | 0.0491979 | 3.51128 | 71.37 | 9.17E-05 | yes |
| RUNX1T1 | chr8 | 0.000204637 | 0.0130133 | 63.59 | 3.04E-14 | yes |
| PCDHA1 | chr5 | 0.00146372 | 0.0868276 | 59.32 | 0 | yes |
| RUNX1T1 | chr8 | 0.000244272 | 0.013015 | 53.28 | 5.74E-13 | yes |
| NRG3 | chr10 | 0.000108064 | 0.00566531 | 52.43 | 0 | yes |
| VPS29 | chr12 | 0.183524 | 8.37666 | 45.64 | 0.000249974 | yes |
| SNX10 | chr7 | 0.00110967 | 0.0455947 | 41.09 | 1.36E-11 | yes |
| GPR85 | chr7 | 0.000190574 | 0.00656369 | 34.44 | 2.10E-08 | yes |
| CD164 | chr6 | 0.120679 | 3.96755 | 32.88 | 0.00153782 | yes |
| PCDHA11 | chr5 | 0.000876634 | 0.0275928 | 31.48 | 2.22E-16 | yes |
| KLHL23 | chr2 | 0.0175034 | 0.519172 | 29.66 | 3.44E-07 | yes |
| APITD1-CORT | chr1 | 0.0489968 | 1.39368 | 28.44 | 3.62E-08 | yes |
| ATP5SL | chr19 | 0.61182 | 16.7429 | 27.37 | 0 | yes |
| ZNF200 | chr16 | 0.00222577 | 0.0598481 | 26.89 | 0 | yes |
| FGFR1 | chr8 | 0.0841256 | 2.06546 | 24.55 | 0 | yes |
| PCSK6 | chr15 | 0.00430437 | 0.10417 | 24.20 | 0.0151812 | yes |
| C20orf30 | chr20 | 0.113772 | 2.74012 | 24.08 | 0.00455133 | yes |
| PCDHA3 | chr5 | 0.0018442 | 0.0411649 | 22.32 | 9.38E-07 | yes |
| SYNJ1 | chr21 | 0.028785 | 0.601202 | 20.89 | 7.33E-15 | yes |
| SYBU | chr8 | 0.00913159 | 0.185641 | 20.33 | 0 | yes |
| SYBU | chr8 | 0.0094626 | 0.185598 | 19.61 | 0 | yes |
| AK3 | chr9 | 0.194366 | 3.74247 | 19.25 | 0 | yes |
| C3orf18 | chr3 | 0.0121824 | 0.214151 | 17.58 | 0.000272861 | yes |
| TCF7 | chr5 | 0.00403203 | 0.0700912 | 17.38 | 1.65E-12 | yes |
| LIPT1 | chr2 | 0.0313182 | 0.541101 | 17.28 | 0.00710277 | yes |
| ZNF641 | chr12 | 0.00156958 | 0.0268638 | 17.12 | 8.79E-08 | yes |
| RUNDC3B | chr7 | 0.00955054 | 0.154671 | 16.19 | 1.34E-09 | yes |
| PITX2 | chr4 | 0.00229719 | 0.0343083 | 14.93 | 0.00410912 | yes |
| DGCR2 | chr22 | 0.232044 | 3.1993 | 13.79 | 0 | yes |
| CAMKK2 | chr12 | 0.060772 | 0.80347 | 13.22 | 3.40E-09 | yes |
| RPPH1 | chr14 | 1.45004 | 18.7436 | 12.93 | 5.38E-13 | yes |
| OGG1 | chr3 | 0.0648263 | 0.834328 | 12.87 | 6.87E-06 | yes |
| PTPN7 | chr1 | 0.0194672 | 0.248467 | 12.76 | 0.011559 | yes |
| LRRC27 | chr10 | 0.0411117 | 0.52355 | 12.73 | 0.000206644 | yes |
| PML | chr15 | 1.75206 | 22.0162 | 12.57 | 0 | yes |
| SCP2 | chr1 | 0.108626 | 1.32614 | 12.21 | 1.49E-13 | yes |
| NAT1 | chr8 | 0.0283872 | 0.346455 | 12.20 | 0.000996836 | yes |
| PTPN7 | chr1 | 0.0221617 | 0.267895 | 12.09 | 0.000927379 | yes |
| PLCB4 | chr20 | 0.501027 | 0.000344402 | -1454.78 | 0 | yes |
| CLEC2D | chr12 | 0.613508 | 0.00129263 | -474.62 | 0 | yes |
| SLC8A3 | chr14 | 0.0032864 | 7.66E-06 | -428.89 | 2.22E-16 | yes |
| RIMS1 | chr6 | 0.128269 | 0.000587384 | -218.37 | 0 | yes |
| PDE11A | chr2 | 0.0040226 | 2.71E-05 | -148.30 | 1.15E-09 | yes |
| ZBTB20 | chr3 | 0.207426 | 0.00143282 | -144.77 | 0 | yes |
| MBNL3 | chrX | 0.0279352 | 0.000204262 | -136.76 | 0 | yes |
| CLCN2 | chr3 | 0.651761 | 0.00478831 | -136.11 | 5.66E-14 | yes |
| ZBTB20 | chr3 | 0.267308 | 0.00199582 | -133.93 | 0 | yes |
| BEX2 | chrX | 0.15642 | 0.00123897 | -126.25 | 9.60E-09 | yes |
| PAX8 | chr2 | 0.484077 | 0.0040161 | -120.53 | 9.93E-10 | yes |
| RNF145 | chr5 | 15.5539 | 0.130296 | -119.37 | 0 | yes |
| SYTL2 | chr11 | 0.0477466 | 0.000410013 | -116.45 | 2.22E-16 | yes |
| SATB1 | chr3 | 0.186366 | 0.00191838 | -97.15 | 0 | yes |
| KIAA0319 | chr6 | 0.00334054 | 3.45E-05 | -96.92 | 1.49E-05 | yes |
| GPR56 | chr16 | 7.59063 | 0.102171 | -74.29 | 0 | yes |
| OSBPL3 | chr7 | 5.56466 | 0.0757432 | -73.47 | 0 | yes |
| CMTM1 | chr16 | 1.72555 | 0.0236626 | -72.92 | 0.00491648 | yes |
| ETV1 | chr7 | 0.141241 | 0.0019386 | -72.86 | 0 | yes |
| ACP5 | chr19 | 0.134922 | 0.00188206 | -71.69 | 0.0157516 | yes |
| PTCH1 | chr9 | 0.00777631 | 0.000109066 | -71.30 | 1.20E-07 | yes |
| ATXN3 | chr14 | 0.178059 | 0.00270236 | -65.89 | 0.00102575 | yes |
| SYBU | chr8 | 0.0143518 | 0.000221917 | -64.67 | 3.72E-06 | yes |
| MRPL33 | chr2 | 44.8923 | 0.712643 | -62.99 | 3.18E-05 | yes |
| RNF145 | chr5 | 11.0372 | 0.18064 | -61.10 | 0 | yes |
| BLCAP | chr20 | 10.6859 | 0.18587 | -57.49 | 0 | yes |
| RORA | chr15 | 1.02717 | 0.0196484 | -52.28 | 0 | yes |
| CCDC66 | chr3 | 1.94061 | 0.0373049 | -52.02 | 4.44E-15 | yes |
| ODF2L | chr1 | 1.36817 | 0.0265551 | -51.52 | 0.000349245 | yes |
| EDA2R | chrX | 1.67322 | 0.0390767 | -42.82 | 1.33E-15 | yes |
| TMC5 | chr16 | 0.00248678 | 5.82E-05 | -42.75 | 0.00101082 | yes |
| NEK6 | chr9 | 14.7531 | 0.373231 | -39.53 | 0 | yes |
| CLEC2D | chr12 | 2.49382 | 0.0660292 | -37.77 | 0 | yes |
| ENSA | chr1 | 12.683 | 0.337963 | -37.53 | 0 | yes |
| MME | chr3 | 0.202543 | 0.00576033 | -35.16 | 0.00019841 | yes |
| FGF1 | chr5 | 0.0151257 | 0.000432192 | -35.00 | 2.12E-05 | yes |
| SUPT5H | chr19 | 11.5136 | 0.348517 | -33.04 | 0 | yes |
| LOC728066 | chr7 | 0.46925 | 0.0144953 | -32.37 | 0 | yes |
| SNX10 | chr7 | 0.247645 | 0.00773706 | -32.01 | 0.000192948 | yes |
| FSTL5 | chr4 | 0.598243 | 0.0196585 | -30.43 | 1.88E-05 | yes |
| IGSF1 | chrX | 0.00637773 | 0.000222654 | -28.64 | 0.000148338 | yes |
| ZBTB20 | chr3 | 0.0412866 | 0.00145223 | -28.43 | 2.92E-11 | yes |
| IRF5 | chr7 | 0.0320979 | 0.00114375 | -28.06 | 8.45E-08 | yes |
| MAP2 | chr2 | 0.627238 | 0.0223705 | -28.04 | 2.83E-10 | yes |
| STX16 | chr20 | 2.90258 | 0.105632 | -27.48 | 0.00106772 | yes |
| CASP8AP2 | chr6 | 1.55003 | 0.0569833 | -27.20 | 5.09E-05 | yes |
| VIT | chr2 | 0.374579 | 0.0139185 | -26.91 | 0.0113388 | yes |
| NAT1 | chr8 | 0.70225 | 0.0270812 | -25.93 | 6.71E-06 | yes |
| SSX2IP | chr1 | 0.542649 | 0.0215575 | -25.17 | 2.65E-11 | yes |
| TNIK | chr3 | 1.19428 | 0.0492985 | -24.23 | 0 | yes |
|  |  |  |  |  |  |  |
|  | | | | | | |
