# Supplementary material for: RNA-seq Reveals Novel Transcriptome of Genes and Their Isoforms in Human Pulmonary Microvascular Endothelial Cells Treated with Thrombin
Source: PLoS One. 2012 Feb 16;7(2):e31229. doi: 10.1371/journal.pone.0031229 (PMC3281071; doi:10.1371/journal.pone.0031229)
Supplement: Table S3 — Top 50 up- and down-regulated novel isoforms in thrombin treated HMVEC cells. Significantly differentially expressed novel isoforms were determined by CuffDiff, after Benjamini-Hochberg correction. The fold change is the ratio of thrombin FPKM to control FPKM. The novel isoforms were ranked on their fold change and the 50 with the highest or lowest fold changes are listed here. (DOCX) [file pone.0031229.s003.docx]

| Table S3: Top 50 up- and down-regulated novel isoforms in thrombin treated HMVEC cells | | | | | |  |
| --- | --- | --- | --- | --- | --- | --- |
|  |  |  |  |  |  |  |
| gene | locus | length | FPKM Control | FPKM Thrombin | Fold Change | p_value |
| ITFG2 | chr12:2921756-2939432 | 2257 | 0.00521437 | 2.2491 | 431.33 | 2.87E-05 |
| SEC16A | chr9:139334541-139378220 | 8173 | 0.0491136 | 7.40764 | 150.83 | 0 |
| PMS2P11 | chr7:76589124-76681310 | 668 | 0.00371097 | 0.359467 | 96.87 | 0 |
| - | chr16:72816785-73092558 | 5767 | 0.00181948 | 0.0994668 | 54.67 | 0 |
| SPTBN2 | chr11:66449988-66496487 | 8426 | 0.0542375 | 2.86062 | 52.74 | 0 |
| MCAM | chr11:119170201-119187947 | 1795 | 0.514138 | 26.8537 | 52.23 | 0 |
| FBN2 | chr5:127593698-127873129 | 4871 | 0.00400303 | 0.187473 | 46.83 | 0.0117604 |
| PHF21A | chr11:45950885-46143027 | 7299 | 0.0236083 | 1.05125 | 44.53 | 0 |
| - | chr19:56115009-56128567 | 3736 | 0.00705679 | 0.306568 | 43.44 | 0.0001756 |
| - | chr19:56115009-56128567 | 947 | 0.00746101 | 0.321088 | 43.04 | 0 |
| SLC25A14 | chrX:129473886-129507335 | 1020 | 0.00591291 | 0.252835 | 42.76 | 0.0141166 |
| ZNF498 | chr7:99214570-99268676 | 3967 | 0.0170222 | 0.720374 | 42.32 | 3.62E-09 |
| RPS3 | chr11:75109801-75116730 | 873 | 1.04909 | 44.0507 | 41.99 | 0 |
| ALG9 | chr11:111652918-111742305 | 1849 | 0.04167 | 1.7419 | 41.8 | 1.30E-05 |
| FANCD2 | chr3:10068110-10143627 | 3324 | 0.00914966 | 0.335769 | 36.7 | 0.00011549 |
| EIF2C2 | chr8:141530086-141645744 | 14475 | 0.211306 | 7.40226 | 35.03 | 0 |
| CCDC24 | chr1:44457401-44489959 | 902 | 0.00976558 | 0.328006 | 33.59 | 1.78E-15 |
| MARCH7 | chr2:160569029-160655380 | 6888 | 0.0138352 | 0.462564 | 33.43 | 0.000413096 |
| - | chr16:81087165-81129967 | 549 | 0.0136895 | 0.434755 | 31.76 | 0 |
| GNB1 | chr1:1716721-1821834 | 2970 | 0.30631 | 9.44177 | 30.82 | 0 |
| ZNF341 | chr20:32319820-32442435 | 3475 | 0.00149754 | 0.0456115 | 30.46 | 3.62E-06 |
| ZMYM2 | chr13:20532809-20665968 | 5906 | 0.00319228 | 0.0965457 | 30.24 | 1.56E-06 |
| TNFRSF9 | chr1:7975858-8000918 | 918 | 0.0627569 | 1.88953 | 30.11 | 6.95E-07 |
| PXDN | chr2:1635589-1748333 | 6712 | 0.183299 | 5.50256 | 30.02 | 0 |
| IFT140 | chr16:1543369-1728160 | 2723 | 0.0211086 | 0.624649 | 29.59 | 1.06E-06 |
| - | chr11:62609045-62609273 | 228 | 0.388571 | 10.481 | 26.97 | 0.00118485 |
| ZNF202 | chr11:123595054-123612307 | 3778 | 0.0320351 | 0.855231 | 26.7 | 7.38E-13 |
| SFPQ | chr1:35641993-35659121 | 6124 | 0.641656 | 15.6102 | 24.33 | 0 |
| LOC729082 | chr15:41576195-41598727 | 5526 | 0.0455855 | 1.0971 | 24.07 | 0.00190821 |
| CSNK1A1 | chr5:148872495-148931132 | 4652 | 0.627922 | 14.3984 | 22.93 | 0.018988 |
| ZNF692 | chr1:249144202-249153315 | 1760 | 0.0212942 | 0.48065 | 22.57 | 3.97E-06 |
| FBRSL1 | chr12:133067022-133161769 | 4324 | 0.03928 | 0.86565 | 22.04 | 7.55E-15 |
| RAB43 | chr3:128811413-128842909 | 556 | 0.211582 | 4.55393 | 21.52 | 0.000130667 |
| - | chr22:44639568-44727593 | 5774 | 0.0282042 | 0.601882 | 21.34 | 3.82E-14 |
| PHF21A | chr11:45950885-46143027 | 7397 | 0.0201042 | 0.420862 | 20.93 | 5.70E-13 |
| - | chr13:46948572-46948674 | 102 | 2779.97 | 57856.7 | 20.81 | 2.56E-06 |
| ZNF630 | chrX:47917566-47931025 | 2563 | 0.00780513 | 0.156306 | 20.03 | 0.0106035 |
| GAK | chr4:842978-926182 | 4524 | 0.0612389 | 1.2231 | 19.97 | 0 |
| ANO7 | chr2:242157281-242165941 | 1709 | 0.0151989 | 0.303374 | 19.96 | 0.00629553 |
| FKRP | chr19:47222676-47261857 | 3355 | 0.0251224 | 0.483553 | 19.25 | 2.56E-07 |
| YEATS2 | chr3:183415662-183528302 | 4325 | 0.3002 | 5.57401 | 18.57 | 0 |
| COL4A6 | chrX:107398836-107681667 | 6677 | 0.0160662 | 0.292462 | 18.2 | 6.30E-09 |
| ABCC3 | chr17:48712217-48769054 | 1718 | 0.0100778 | 0.170975 | 16.97 | 0.000237761 |
| SLC38A7 | chr16:58699090-58718689 | 1739 | 0.087685 | 1.4768 | 16.84 | 0.000170914 |
| SELE | chr1:169691788-169703334 | 3837 | 0.165732 | 2.76911 | 16.71 | 0.000960609 |
| ZNF846 | chr19:9867523-9896820 | 936 | 0.0287307 | 0.476473 | 16.58 | 0.000743379 |
| SPTBN2 | chr11:66449988-66496487 | 9997 | 0.040015 | 0.632404 | 15.8 | 0 |
| SEL1L3 | chr4:25732675-25864610 | 3453 | 0.528664 | 8.04417 | 15.22 | 4.47836E-06 |
| RTN4 | chr2:55199318-55278324 | 1481 | 10.4564 | 158.156 | 15.13 | 0 |
| SEL1L3 | chr4:25723495-25906202 | 3489 | 0.439247 | 6.42823 | 14.63 | 9.66E-07 |
| ZNF518A | chr10:97889630-97924859 | 9138 | 0.313598 | 0.000338024 | -927.74 | 0.018282 |
| MYST4 | chr10:76585061-76792325 | 7557 | 0.124027 | 0.000147896 | -838.61 | 0 |
| ZNF518A | chr10:97889630-97924859 | 9351 | 0.319225 | 0.000432489 | -738.11 | 0.0194973 |
| - | chr3:101043117-101232085 | 452 | 1.25099 | 0.003021 | -414.1 | 0 |
| ZNF518A | chr10:97889630-97924859 | 9452 | 0.216304 | 0.000826257 | -261.79 | 0.002249 |
| - | chr10:27855765-27870612 | 1031 | 0.27927 | 0.00107217 | -260.47 | 0 |
| RBMX | chrX:135951349-135962911 | 4282 | 3.57287 | 0.0152633 | -234.08 | 0 |
| E2F6 | chr2:11584508-11606270 | 1439 | 0.374663 | 0.00191853 | -195.29 | 0.0034366 |
| PTBP2 | chr1:97187347-97280539 | 3131 | 0.505582 | 0.00259479 | -194.84 | 0.0153448 |
| - | chr1:233086321-233431177 | 1397 | 0.315314 | 0.00170047 | -185.43 | 0 |
| ZNF518A | chr10:97889630-97924859 | 9899 | 0.158119 | 0.00101905 | -155.16 | 0.00184869 |
| ZDBF2 | chr2:207139642-207179094 | 10053 | 0.524093 | 0.00389446 | -134.57 | 1.27278E-07 |
| BRCC3 | chrX:154301645-154353185 | 4014 | 1.67684 | 0.0139926 | -119.84 | 2.58E-07 |
| RMI1 | chr9:86595633-86618987 | 3338 | 0.477557 | 0.00424504 | -112.5 | 0.000524425 |
| ERV3 | chr7:64450020-64467328 | 3293 | 19.8048 | 0.199357 | -99.34 | 0 |
| RNF217 | chr6:125269090-125413776 | 10535 | 1.21081 | 0.0127423 | -95.02 | 8.27747E-06 |
| UBE2Q2P1 | chr15:85053060-85114005 | 2043 | 2.00998 | 0.0215198 | -93.4 | 0.000134704 |
| LRRC66 | chr4:52859852-52884562 | 1308 | 0.283939 | 0.00318956 | -89.02 | 0 |
| - | chr1:231114822-231142905 | 2904 | 0.272874 | 0.00321634 | -84.84 | 0 |
| - | chr13:50617701-50649788 | 2012 | 0.223118 | 0.00271523 | -82.17 | 0 |
| CIRBP | chr19:1268805-1274730 | 1604 | 5.00386 | 0.0613439 | -81.57 | 9.47294E-10 |
| - | chr10:81460630-81600715 | 1035 | 0.155446 | 0.00194186 | -80.05 | 0 |
| PSG4 | chr19:43696853-43709805 | 1749 | 4.03833 | 0.0515632 | -78.32 | 4.3467E-06 |
| - | chr3:27148561-27387642 | 554 | 0.650204 | 0.00833192 | -78.04 | 0 |
| - | chr17:66970735-67058587 | 5121 | 0.509551 | 0.00702453 | -72.54 | 1.52436E-06 |
| PTTG1IP | chr21:46269383-46295722 | 4727 | 6.47078 | 0.0898634 | -72.01 | 0 |
| ICAM4 | chr19:10380332-10399182 | 5835 | 5.60927 | 0.078795 | -71.19 | 0 |
| PLXNA3 | chrX:153686622-153703438 | 8088 | 0.842041 | 0.01203 | -70 | 8.91E-13 |
| - | chr17:18414528-18491653 | 2076 | 1.06526 | 0.0154991 | -68.73 | 4.59642E-06 |
| HN1 | chr17:73131339-73150775 | 1320 | 40.7287 | 0.618528 | -65.85 | 0 |
| WDR26 | chr1:224572844-224709031 | 6064 | 2.43156 | 0.036992 | -65.73 | 0 |
| ZNF587 | chr19:58281034-58400442 | 15455 | 0.618125 | 0.00945956 | -65.34 | 0 |
| - | chr3:145787229-145968966 | 1433 | 0.884352 | 0.0140455 | -62.96 | 0 |
| HAUS3 | chr4:2073688-2243870 | 6147 | 0.271122 | 0.00434312 | -62.43 | 0.00173626 |
| DMXL1 | chr5:118406742-118584841 | 2042 | 0.822321 | 0.0142524 | -57.7 | 0.0176546 |
| CENPE | chr4:104026963-104119534 | 8084 | 2.80976 | 0.0540036 | -52.03 | 0.00734551 |
| GABBR1 | chr6:29568982-29600950 | 7320 | 2.10375 | 0.0407709 | -51.6 | 0 |
| - | chr12:44194028-44200102 | 586 | 0.513337 | 0.0104367 | -49.19 | 0 |
| DNAH5 | chr5:13690490-13769155 | 3183 | 0.128837 | 0.00263481 | -48.9 | 0 |
| - | chr3:145787229-145968966 | 2034 | 0.301187 | 0.00623089 | -48.34 | 0 |
| - | chr1:59226669-59236398 | 840 | 0.594298 | 0.0126197 | -47.09 | 0 |
| PTPRB | chr12:70909770-71058476 | 12565 | 8.02045 | 0.170458 | -47.05 | 0 |
| - | chr11:74459982-74660193 | 1881 | 0.301812 | 0.00644505 | -46.83 | 0 |
| ZNF323 | chr6:28272993-28367527 | 13626 | 0.38497 | 0.00837745 | -45.95 | 4.89748E-06 |
| - | chr3:145787229-145968966 | 3584 | 0.650945 | 0.0143438 | -45.38 | 1.17037E-07 |
| SET | chr9:131445759-131458695 | 3035 | 4.83259 | 0.108469 | -44.55 | 0.000398784 |
| - | chr1:79336558-79340119 | 2385 | 0.290277 | 0.00653386 | -44.43 | 0.00430491 |
| ZNF643 | chr1:40915743-40932629 | 5514 | 0.296526 | 0.00671472 | -44.16 | 0.0227236 |
| - | chr10:4798311-4823110 | 1118 | 0.199259 | 0.00454806 | -43.81 | 0 |
| - | chr7:91827572-91875208 | 1853 | 0.318672 | 0.00728576 | -43.74 | 0 |
|  |  |  |  |  |  |  |
|  | | | | | | |
|  |  |  |  |  |  |  |
