# Supplementary material for: RNA-seq Reveals Novel Transcriptome of Genes and Their Isoforms in Human Pulmonary Microvascular Endothelial Cells Treated with Thrombin
Source: PLoS One. 2012 Feb 16;7(2):e31229. doi: 10.1371/journal.pone.0031229 (PMC3281071; doi:10.1371/journal.pone.0031229)
Supplement: Table S4 — Differentially expressed genes and isoforms in Thrombin Signaling Pathway* * Fold change was calculated by Fragments Per Kilobase of exon per Million fragments mapped (FPKM) in Thrombin treated group dividing FPKM in control group. (DOCX) [file pone.0031229.s004.docx]

Table S4 Differentially expressed genes and isoforms in Thrombin Signaling Pathway*

| Down Regulated Genes | | | | | | |
| --- | --- | --- | --- | --- | --- | --- |
| Gene Symbol | Overall Fold Change | Members | Individual Fold Change | p_value | FPKM Control | FPKM Thrombin |
| PLC | -2.49 |  |  |  |  |  |
|  |  | PLCB1 | -2.97 | 0 | 6.75585 | 2.27611 |
|  |  | PLCB2 | -1.32 | 0.00183634 | 1.80892 | 1.36957 |
|  |  | PLCB4 | -10.04 | 6.28386E-14 | 0.682668 | 0.0679837 |
|  |  | PLCL1 | -2.53 | 5.26728E-09 | 0.602879 | 0.238017 |
| Gaq | -1.87 |  |  |  |  |  |
|  |  | GNAQ | -1.87 | 0 | 24.0907 | 12.8996 |
| Gai | -1.56 |  |  |  |  |  |
|  |  | GNAI1 | -1.86 | 0 | 9.88417 | 5.31795 |
|  |  | GNAI3 | -1.49 | 0 | 35.7592 | 24.0198 |
| PKC |  |  | -2.15 |  |  |  |
|  |  | PRKCE | -1.65 | 0 | 9.36364 | 5.67305 |
|  |  | PRKCI | -2.14 | 0 | 6.63566 | 3.09818 |
|  |  | PRKD3 | -2.61 | 0 | 16.0215 | 6.12878 |
| IP3R | -2.60 |  |  |  |  |  |
|  |  | ITPR1 | -1.39 | 0.000018734 | 1.51592 | 1.09009 |
|  |  | ITPR2 | -3.19 | 0 | 7.23283 | 2.26944 |
| CREB | -2.36 |  |  |  |  |  |
|  |  | CREB1 | -2.36 | 0 | 5.19117 | 2.2004 |
| GATA | -2.33 |  |  |  |  |  |
|  |  | GATA3 | -2.33 | 0.0228108 | 0.716773 | 0.307131 |
| TBP | -1.35 |  |  |  |  |  |
|  |  | TBP | -1.35 | 2.66E-05 | 8.48689 | 6.30476 |
| G-protein Alpha | -1.64 |  |  |  |  |  |
|  |  | GNA14 | -1.49 | 0.000122496 | 2.78076 | 1.86573 |
|  |  | GNAI1 | -1.86 | 0 | 9.88417 | 5.31795 |
|  |  | GNAI3 | -1.49 | 0 | 35.7592 | 24.0198 |
|  |  | GNAQ | -1.87 | 0 | 24.0907 | 12.8996 |
| PAR3 | -1.87 |  |  |  |  |  |
|  |  | F2RL2 | -1.87 | 1.02474E-06 | 1.51286 | 0.810286 |
| SOS1 |  |  |  |  |  |  |
|  |  | SOS1 | -2.27 | 0 | 8.94353 | 3.94679 |
| Ras | -1.69 |  |  |  |  |  |
|  |  | KRAS | -2.03 | 0 | 11.2766 | 5.5659 |
|  |  | NRAS | -1.61 | 0 | 38.0874 | 23.6491 |
| AKT | -1.77 |  |  |  |  |  |
|  |  | AKT3 | -1.77 | 0 | 15.8228 | 8.94223 |
| MLCP | -2.38 |  |  |  |  |  |
|  |  | PPP1CB | -2.10 | 0 | 39.585 | 18.8199 |
|  |  | PPP1R12A | -3.56 | 0 | 15.2274 | 4.27516 |
|  |  | PPP1R12B | -2.66 | 5.28466E-13 | 1.92123 | 0.722188 |
| FAK | -1.31 |  |  |  |  |  |
|  |  | PTK2 | -1.31 | 4.3856E-10 | 39.7935 | 30.3044 |
| p70 S6K |  |  |  |  |  |  |
|  |  | RPS6KB1 | -1.99 | 0 | 8.46312 | 4.25129 |
| ROCK | -3.68 |  |  |  |  |  |
|  |  | ROCK1 | -3.54 | 0 | 19.5921 | 5.53112 |
|  |  | ROCK2 | -3.86 | 0 | 16.0054 | 4.14759 |
| CAMK | -1.17 |  |  |  |  |  |
|  |  | CAMK1 | 1.30 | 0.000255587 | 7.90794 | 10.296 |
|  |  | CAMK2D | -1.74 | 2.22911E-12 | 11.4497 | 6.56804 |
|  |  | CAMK4 | -2.58 | 0.000619045 | 0.62714 | 0.243277 |
|  |  |  |  |  |  |  |
| Up regulated Genes | | | | | | |
| Symbol | Overall Fold Change | Members | Individual Fold Change | p_value | FPKM Control | FPKM Thrombin |
| PAR4 | 1.59 |  |  |  |  |  |
|  |  | F2RL3 | 1.59 | 9.19043E-13 | 4.99867 | 7.9253 |
| Src | 1.47 |  |  |  |  |  |
|  |  | Src | 1.47 | 0 | 14.1085 | 20.675 |
| NF-kB | 1.65 |  |  |  |  |  |
|  |  | NFKB1 | 1.66 | 0 | 19.5113 | 32.3457 |
|  |  | NFKB2 | 2.02 | 0 | 28.7563 | 58.1293 |
|  |  | RELA | 1.45 | 0 | 55.3482 | 80.28 |
|  |  |  |  |  |  |  |
| Partial up/partial down Genes | | | | | | |
| Symbol | Overall Fold Change | Members | Individual Fold Change | p_value | FPKM Control | FPKM Thrombin |
| G-protein gamma | 1.22 |  |  |  |  |  |
|  |  | GNG10 | -1.34 | 2.17216E-08 | 27.192 | 20.2206 |
|  |  | GNG11 | 1.31 | 5.66209E-05 | 770.922 | 1011.05 |
|  |  | GNG12 | -1.44 | 5.11591E-13 | 112.397 | 78.3023 |
|  |  | GNG2 | 2.43 | 6.38453E-09 | 0.465705 | 1.13132 |
| G-protein beta | 1.27 |  |  |  |  |  |
|  |  | GNB2 | 1.36 | 1.91268E-10 | 137.786 | 187.43 |
|  |  | GNB3 | -2.69 | 4.71259E-11 | 2.58703 | 0.962504 |
|  |  | GNB4 | -1.61 | 0 | 15.8275 | 9.85484 |
| Rho GEF | -1.16 |  |  |  |  |  |
|  |  | ARHGEF12 | -1.67 | 0 | 30.6424 | 18.3015 |
|  |  | ARHGEF2 | 1.33 | 5.80478E-08 | 27.6288 | 36.758 |
|  |  | ARHGEF3 | -1.48 | 0 | 20.3279 | 13.7813 |
|  |  | ARHGEF6 | -2.08 | 0 | 2.67944 | 1.28635 |
|  |  | ARHGEF9 | -1.54 | 0.00469498 | 1.56367 | 1.0159 |
| PI3K | -1.80 |  |  |  |  |  |
|  |  | ATM | -6.84 | 0 | 4.98972 | 0.729483 |
|  |  | PIK3C2A | -5.09 | 0 | 17.7894 | 3.49234 |
|  |  | PIK3C3 | -1.49 | 4.66294E-15 | 12.6504 | 8.50852 |
|  |  | PIK3CA | -3.14 | 0 | 16.8398 | 5.36228 |
|  |  | PIK3CB | -1.36 | 8.4357E-09 | 9.68516 | 7.12129 |
|  |  | PIK3CD | 1.86 | 0 | 5.6579 | 10.5507 |
|  |  | PIK3CG | -1.76 | 2.32945E-10 | 1.86962 | 1.06419 |
|  |  | PIK3R1 | -1.79 | 0 | 5.48118 | 3.06256 |
|  |  | PIK3R3 | -1.59 | 1.50915E-05 | 5.24549 | 3.30763 |
|  |  | PIK3R4 | -1.40 | 7.18425E-12 | 8.34176 | 5.97942 |
| Rho | 1.19 |  |  |  |  |  |
|  |  | RHOB | 1.31 | 9.48429E-06 | 232.232 | 304.587 |
|  |  | RHOC | 1.38 | 0 | 458.267 | 630.235 |
|  |  | RHOF | 1.65 | 0 | 8.29676 | 13.7268 |
|  |  | RHOG | 1.40 | 1.02141E-14 | 58.6003 | 82.0172 |
|  |  | RHOJ | -1.57 | 0 | 127.446 | 80.9317 |
|  |  | RHOQ | -1.52 | 0 | 16.4802 | 10.8122 |
|  |  | RHOT1 | -1.96 | 3.00071E-12 | 8.48834 | 4.33755 |
|  |  | RHOU | -1.54 | 0.00184367 | 0.900141 | 0.586092 |
|  |  | RHOV | -3.32 | 0.00564671 | 0.413912 | 0.124591 |
|  |  | RND3 | -1.95 | 0 | 58.0564 | 29.7075 |
| MLC | -1.06 |  |  |  |  |  |
|  |  | MYL12B | -1.43 | 4.87832E-11 | 872.075 | 608.472 |
|  |  |  |  |  |  |  |
|  |  | MYL9 | 1.48 | 0 | 202.636 | 299.559 |
