# Supplementary material for: RNA-seq Reveals Novel Transcriptome of Genes and Their Isoforms in Human Pulmonary Microvascular Endothelial Cells Treated with Thrombin
Source: PLoS One. 2012 Feb 16;7(2):e31229. doi: 10.1371/journal.pone.0031229 (PMC3281071; doi:10.1371/journal.pone.0031229)
Supplement: Table S5 — Top ten up- and down- regulated genes showing alternative splicing in thrombin treated HMVEC Cells. The top 10 up- and down-regulated genes that exhibit significant alternative splicing according to CuffDiff were presented. (DOCX) [file pone.0031229.s005.docx]

| Table S5: Top ten up- and down- regulated genes showing alternative splicing in thrombin treated HMVEC Cells | | | | | |
| --- | --- | --- | --- | --- | --- |
|  |  |  |  |  |  |
| Gene | Description | Chr. | Fold Change | Splicing p-value | Significant |
| *PCDHA1* | protocadherin alpha 1 | chr5 | 94.23 | 0 | yes |
| *PCDHA11* | protocadherin alpha 11 | chr5 | 58.50 | 0 | yes |
| *NR4A1* | nuclear receptor subfamily 4, group A, member 1 | chr12 | 6.11 | 2.44886E-09 | yes |
| *PCDHA3* | protocadherin alpha 3 | chr5 | 5.92 | 0 | yes |
| *PCDHA10* | protocadherin alpha 10 | chr5 | 5.26 | 0 | yes |
| *IL34* | interleukin 34 | chr16 | 4.12 | 5.86234E-07 | yes |
| *CLDN14* | claudin 14 | chr21 | 3.64 | 0.00028195 | yes |
| *SLC7A2* | solute carrier family 7 (cationic amino acid transporter, y+ system),  member 2 | chr8 | 2.93 | 0.0103523 | yes |
| *FNDC5* | fibronectin type III domain containing 5 | chr1 | 2.77 | 0.000146986 | yes |
| *CSF3* | colony stimulating factor 3 (granulocyte) | chr17 | 2.59 | 0.00143392 | yes |
| *ACRV1* | acrosomal vesicle protein 1 | chr11 | -46.53 | 0 | yes |
| *CD37* | CD37 molecule | chr19 | -16.32 | 0 | yes |
| *FMR1-AS1* | FMR1 antisense RNA 1 | chrX | -12.97 | 0 | yes |
| *IDI2-AS1* | IDI2 antisense RNA 1 | chr10 | -8.88 | 2.84015E-07 | yes |
| *PCDHA12* | protocadherin alpha 12 | chr5 | -6.46 | 0 | yes |
| *ZNF83* | zinc finger protein 83 | chr19 | -6.12 | 0.00966847 | yes |
| *PCDHA6* | protocadherin alpha 6 | chr5 | -6.10 | 0 | yes |
| *RIF1* | RAP1 interacting factor homolog (yeast) | chr2 | -6.04 | 0 | yes |
| *GUCY1A3* | guanylate cyclase 1, soluble, alpha 3 | chr4 | -5.71 | 2.80E-11 | yes |
| *GUCY1A3* | guanylate cyclase 1, soluble, alpha 3 | chr4 | -5.71 | 8.88E-15 | yes |
|  |  |  |  |  |  |
