# Supplementary material for: RNA-seq Reveals Novel Transcriptome of Genes and Their Isoforms in Human Pulmonary Microvascular Endothelial Cells Treated with Thrombin
Source: PLoS One. 2012 Feb 16;7(2):e31229. doi: 10.1371/journal.pone.0031229 (PMC3281071; doi:10.1371/journal.pone.0031229)
Supplement: Table S6 — Top 50 up- and down-regulated genes with alternative promoter usage in thrombin treated HMVEC cells. The 50 top up- and down- regulated genes after thrombin treatment with any significant alternative promoter usage were determined by CuffDiff. (DOCX) [file pone.0031229.s006.docx]

| Table S6: Top 50 up- and down-regulated genes with alternative promoter usage in thrombin treated HMVEC cells, | | | | |
| --- | --- | --- | --- | --- |
|  |  |  |  |  |
| Gene | Chromosome | Fold Change | Promoter p_value | significant |
| EGR1 | chr5 | 3.33 | 1 | no |
| TGIF2-C20ORF24 | chr20 | 3.35 |  |  |
| ICOSLG | chr21 | 3.37 | 1 | no |
| JAG1 | chr20 | 3.38 | 1 | no |
| KCNF1 | chr2 | 3.39 | 1 | no |
| CXCR7 | chr2 | 3.43 | 1 | no |
| PRR16 | chr5 | 3.46 | 1 | no |
| SNORD10 | chr17 | 3.48 | 1 | no |
| NOD2 | chr16 | 3.60 | 1 | no |
| TMEM158 | chr3 | 3.64 | 1 | no |
| SEMA7A | chr15 | 3.64 | 0.979059 | no |
| CLDN14 | chr21 | 3.64 | 0 | yes |
| TMCC2 | chr1 | 3.69 | 1 | no |
| EPPK1 | chr8 | 3.79 | 1 | no |
| SP6 | chr17 | 3.86 | 1 | no |
| CD83 | chr6 | 3.92 | 1 | no |
| CA2 | chr8 | 4.03 | 1 | no |
| CYGB | chr17 | 4.09 | 1 | no |
| IL34 | chr16 | 4.12 | 0.773995 | no |
| C2CD4A | chr15 | 4.13 | 1 | no |
| C1QTNF1 | chr17 | 4.14 | 0.405573 | no |
| NR4A3 | chr9 | 4.24 | 0.64863 | no |
| KCNN2 | chr5 | 4.25 | 0.0900325 | no |
| IL1B | chr2 | 4.26 | 1 | no |
| KIAA1644 | chr22 | 4.44 | 1 | no |
| RND1 | chr12 | 4.50 | 1 | no |
| VCAM1 | chr1 | 4.75 | 1 | no |
| NCF4 | chr22 | 4.84 | 1 | no |
| ADAMTS4 | chr1 | 4.84 | 1 | no |
| BDKRB2 | chr14 | 4.93 | 1 | no |
| TP63 | chr3 | 4.94 | 0.971568 | no |
| MRGPRX3 | chr11 | 5.00 | 1 | no |
| SELE | chr1 | 5.13 | 1 | no |
| PCDHA10 | chr5 | 5.26 | 1 | no |
| LIF | chr22 | 5.52 | 1 | no |
| CSF2 | chr5 | 5.54 | 1 | no |
| METTL7B | chr12 | 5.69 | 1 | no |
| TLR2 | chr4 | 5.72 | 1 | no |
| PCDHA3 | chr5 | 5.92 | 1 | no |
| CCL20 | chr2 | 5.95 | 1 | no |
| CX3CL1 | chr16 | 6.05 | 1 | no |
| NR4A1 | chr12 | 6.11 | 1 | no |
| HUS1B | chr6 | 6.71 | 1 | no |
| LOC441617 | chr11 | 6.80 | 1 | no |
| AS3MT | chr10 | 6.90 | 1 | no |
| RMRP | chr9 | 7.15 | 1 | no |
| TRAF1 | chr9 | 7.96 | 0.0010364 | yes |
| RPPH1 | chr14 | 12.93 | 1 | no |
| PCDHA11 | chr5 | 58.50 | 1 | no |
| PCDHA1 | chr5 | 94.23 | 1 | no |
| GHRLOS | chr3 | -75.30 | 1 | no |
| IGSF10 | chr3 | -49.97 | 0 | yes |
| ACRV1 | chr11 | -46.53 | 0 | yes |
| LOC728066 | chr7 | -32.37 | 1 | no |
| LOC100132215 | chr2 | -22.21 | 1 | no |
| CCDC144C | chr17 | -21.53 | 1 | no |
| C2orf66 | chr2 | -19.62 | 1 | no |
| MLANA | chr9 | -18.94 | 1 | no |
| TEX15 | chr8 | -18.15 | 1 | no |
| CD37 | chr19 | -16.32 | 1 | no |
| DNAH8 | chr6 | -15.87 | 1 | no |
| GALNT3 | chr2 | -15.33 | 1 | no |
| KLRAP1 | chr12 | -15.20 | 1 | no |
| CCDC39 | chr3 | -14.74 | 1 | no |
| LOC338588 | chr10 | -14.56 | 1 | no |
| C10orf62 | chr10 | -14.54 | 1 | no |
| LOC221442 | chr6 | -14.49 | 1 | no |
| WDR52 | chr3 | -13.76 | 1 | no |
| CCDC144A | chr17 | -13.58 | 1 | no |
| SLC24A5 | chr15 | -13.53 | 1 | no |
| TSIX | chrX | -13.38 | 1 | no |
| FMR1-AS1 | chrX | -12.97 | 1 | no |
| LRRC39 | chr1 | -12.77 | 1 | no |
| GAS2 | chr11 | -12.14 | 0.699403 | no |
| SESN3 | chr11 | -11.85 | 1 | no |
| KCNJ13 | chr2 | -11.84 | 1 | no |
| CCDC152 | chr5 | -11.69 | 1 | no |
| GGT8P | chr2 | -11.58 | 1 | no |
| ZNF117 | chr7 | -11.49 | 1 | no |
| HSD17B3 | chr9 | -11.18 | 1 | no |
| CCDC144B | chr17 | -11.18 | 1 | no |
| IMPG1 | chr6 | -11.03 | 1 | no |
| LOC220594 | chr17 | -10.74 | 1 | no |
| LOC162632 | chr17 | -10.73 | 1 | no |
| CCDC146 | chr7 | -10.58 | 1 | no |
| LOC100130872,SPON2 | chr4 | -10.50 | 1 | no |
| AMY2B | chr1 | -10.45 | 1 | no |
| CG030 | chr13 | -10.17 | 1 | no |
| FAM106A | chr17 | -10.10 | 1 | no |
| PLCB4 | chr20 | -10.04 | 0 | yes |
| GOLGA8IP | chr15 | -9.86 | 1 | no |
| VIP | chr6 | -9.85 | 1 | no |
| SLC25A27 | chr6 | -9.77 | 1 | no |
| C8orf77 | chr8 | -9.68 | 1 | no |
| ABCA9 | chr17 | -9.62 | 1 | no |
| LOC643596 | chr9 | -9.54 | 1 | no |
| LRGUK | chr7 | -9.54 | 1 | no |
| CENPE | chr4 | -9.52 | 1 | no |
| NOXA1 | chr9 | -9.52 | 1 | no |
| RBM44 | chr2 | -9.48 | 1 | no |
