# Supplementary material for: RNA-seq Reveals Novel Transcriptome of Genes and Their Isoforms in Human Pulmonary Microvascular Endothelial Cells Treated with Thrombin
Source: PLoS One. 2012 Feb 16;7(2):e31229. doi: 10.1371/journal.pone.0031229 (PMC3281071; doi:10.1371/journal.pone.0031229)
Supplement: Table S7 — RNA Seq Data from 6 h thrombin-treated HMVEC vs Microarray Data from 6 h thrombin-treated HUVEC * * The data from this study is compared to the study by Uzonyi et al. (23) in which gene expression profile was assayed by Affymetrix Human Genome U133A Array. (DOCX) [file pone.0031229.s007.docx]

Table S7 RNA Seq Data from 6 h thrombin-treated HMVEC vs Microarray Data from 6 h thrombin-treated HUVEC *

| Up-regulated in both datasets (39) | | | |
| --- | --- | --- | --- |
| Gene Symbol | Representative Public ID | RNA Seq Fold Change | Microarray Fold Change |
| CX3CL1 | U84487 | 6.05 | 2.03 |
| VCAM1 | NM_001078 | 4.75 | 4.16 |
| KCNN2 | NM_021614 | 4.25 | 2.83 |
| NR4A3 | X89894 | 4.24 | 2.34 |
| JAG1 | U77914 | 3.38 | 1.25 |
| EGR1 | NM_001964 | 3.33 | 1.97 |
| ICAM1 | AI608725 | 3.29 | 3.14 |
| ICAM1 | NM_000201 | 3.29 | 3.64 |
| ETV7 | AF147782 | 3.09 | 1.51 |
| BMP2 | AA583044 | 3.06 | 1.34 |
| BMP2 | NM_001200 | 3.06 | 1.40 |
| DUSP5 | U16996 | 2.96 | 1.54 |
| IL8 | NM_000584 | 2.78 | 6.82 |
| SYNM | AK026420 | 2.66 | 1.61 |
| HBEGF | M60278 | 2.65 | 1.50 |
| NUAK2 | NM_030952 | 2.58 | 1.65 |
| NDRG1 | NM_006096 | 2.54 | 1.45 |
| MYO1E | NM_004998 | 2.47 | 1.30 |
| SPRY4 | W48843 | 2.45 | 1.82 |
| LITAF | NM_004862 | 2.43 | 1.25 |
| ISG20 | U88964 | 2.42 | 1.33 |
| CNIH3 | AF070524 | 2.36 | 1.55 |
| PPM1H | AB032983 | 2.35 | 1.43 |
| SOD2 | X15132 | 2.35 | 1.74 |
| SOD2 | BF575213 | 2.35 | 1.80 |
| SOD2 | W46388 | 2.35 | 2.38 |
| RELB | NM_006509 | 2.33 | 1.66 |
| TNIP1 | NM_006058 | 2.28 | 1.52 |
| IL6 | NM_000600 | 2.27 | 1.34 |
| RAB11FIP1 | NM_025151 | 2.22 | 1.34 |
| TFPI2 | L27624 | 2.21 | 1.63 |
| TFPI2 | AL574096 | 2.21 | 1.84 |
| PLAUR | AY029180 | 2.15 | 1.51 |
| PLAUR | U08839 | 2.15 | 1.63 |
| PDGFB | AU150748 | 2.06 | 1.75 |
| PDGFB | NM_002608 | 2.06 | 2.11 |
| NFKB2 | NM_002502 | 2.02 | 1.41 |
| GBP1 | BC002666 | 2.01 | 1.63 |
| RASSF2 | NM_014737 | 2.00 | 1.20 |
|  |  |  |  |
| Down-regulated in both datasets (396) | | | |
| Gene Symbol | Representative Public ID | RNA Seq Fold Change | Microarray Fold Change |
| GAS2 | NM_005256 | -12.14 | -1.74 |
| PLCB4 | AL535113 | -10.04 | -1.81 |
| VPS13A | AB023203 | -9.00 | -1.23 |
| AKAP9 | AK000270 | -8.51 | -1.51 |
| ASPM | NM_018123 | -8.26 | -1.34 |
| SAMD9 | NM_017654 | -8.15 | -1.39 |
| ANKRD12 | AW572909 | -7.88 | -1.27 |
| ZNF518A | NM_014803 | -7.78 | -1.41 |
| ZNF292 | BF223237 | -7.63 | -2.01 |
| ZNF292 | AA972711 | -7.63 | -1.48 |
| CHD9 | AI742305 | -7.54 | -1.48 |
| EEA1 | NM_003566 | -7.35 | -1.21 |
| ATRX | U09820 | -7.30 | -1.38 |
| NKTR | AI688640 | -7.15 | -1.34 |
| CCDC88A | NM_018084 | -6.78 | -1.36 |
| PRKACB | NM_002731 | -6.23 | -1.24 |
| HLTF | AI760760 | -6.17 | -1.26 |
| ARHGAP28 | NM_030672 | -6.14 | -1.58 |
| ZNF83 | M27877 | -6.12 | -1.28 |
| RIF1 | AK000323 | -6.04 | -1.30 |
| TRIP11 | AF007217 | -6.03 | -1.21 |
| OCLM | NM_022375 | -5.74 | -1.23 |
| HMMR | U29343 | -5.71 | -1.21 |
| BAZ2B | NM_013450 | -5.61 | -1.35 |
| TPR | AK023111 | -5.60 | -1.30 |
| RB1CC1 | NM_014781 | -5.58 | -1.26 |
| RB1CC1 | BG402105 | -5.58 | -1.25 |
| ZNF638 | AI357871 | -5.57 | -1.59 |
| ZNF638 | AF273049 | -5.57 | -1.21 |
| KIAA1109 | AL137384 | -5.49 | -1.63 |
| KIAA1109 | AB029032 | -5.49 | -1.42 |
| ZNF91 | NM_003430 | -5.44 | -1.39 |
| SFRS18 | AW157501 | -5.43 | -1.77 |
| SFRS18 | AW081113 | -5.43 | -1.32 |
| SFRS18 | AA902326 | -5.43 | -1.22 |
| SEMA6A | AB002438 | -5.42 | -2.14 |
| SEMA6A | NM_020796 | -5.42 | -1.64 |
| CFHR1 | X56210 | -5.41 | -1.20 |
| PDK4 | NM_002612 | -5.39 | -2.02 |
| DMXL2 | AB020663 | -5.38 | -1.23 |
| REV3L | NM_002912 | -5.17 | -1.43 |
| CEP350 | NM_014810 | -5.16 | -1.33 |
| PHIP | BG545769 | -5.16 | -1.63 |
| PHIP | BF224151 | -5.16 | -1.52 |
| PCM1 | NM_006197 | -5.15 | -1.26 |
| SMC4 | AL136877 | -5.14 | -1.20 |
| ZFYVE16 | NM_014733 | -5.13 | -1.32 |
| SENP7 | NM_020654 | -5.12 | -2.57 |
| KIF14 | NM_014875 | -5.10 | -1.24 |
| HERC2P2 | AB002391 | -5.07 | -1.24 |
| TTC3 | D83077 | -5.02 | -1.22 |
| DYNC2H1 | NM_024606 | -5.01 | -1.54 |
| PGAP1 | AV705244 | -5.01 | -2.14 |
| ZFP112 | AC084239 | -4.91 | -1.29 |
| CENPC1 | NM_001812 | -4.80 | -1.40 |
| PHF3 | BF430956 | -4.79 | -1.33 |
| PHF3 | AI949220 | -4.79 | -1.32 |
| PHF3 | NM_015153 | -4.79 | -1.30 |
| SRSF2IP | NM_004719 | -4.75 | -1.26 |
| PPIG | AW340788 | -4.64 | -1.49 |
| HMGN5 | NM_030763 | -4.55 | -1.24 |
| YTHDC2 | NM_022828 | -4.51 | -1.32 |
| SMCHD1 | AB014550 | -4.49 | -1.27 |
| PIBF1 | NM_006346 | -4.45 | -1.31 |
| EVI5 | NM_005665 | -4.43 | -1.45 |
| CMAH | NM_003570 | -4.41 | -1.23 |
| STAG2 | BC001765 | -4.39 | -1.33 |
| POLI | NM_007195 | -4.37 | -1.25 |
| NEK1 | AV700007 | -4.32 | -1.20 |
| ESF1 | NM_016649 | -4.20 | -1.36 |
| SR140 | AU152088 | -4.18 | -1.56 |
| SR140 | AI184562 | -4.18 | -1.22 |
| CCPG1 | AK022459 | -4.17 | -1.33 |
| RUNX1T1 | NM_004349 | -4.09 | -2.00 |
| RUNX1T1 | X79990 | -4.09 | -1.35 |
| PTBP2 | NM_021190 | -4.08 | -1.40 |
| N4BP2L2 | AI809961 | -4.06 | -1.68 |
| N4BP2L2 | AW084068 | -4.06 | -1.20 |
| NFAT5 | NM_006599 | -4.03 | -1.26 |
| ZNF224 | NM_005774 | -4.01 | -1.71 |
| ZNF451 | AB011148 | -3.99 | -1.29 |
| CKAP2 | NM_018204 | -3.96 | -1.28 |
| FAM178A | AL133215 | -3.93 | -1.37 |
| TOP2A | AU159942 | -3.91 | -2.80 |
| TOP2A | AL561834 | -3.91 | -1.53 |
| MBNL2 | BE328496 | -3.91 | -1.28 |
| USP8 | NM_005154 | -3.90 | -1.49 |
| CCDC76 | NM_019083 | -3.90 | -1.55 |
| SLK | NM_014720 | -3.89 | -1.30 |
| SMC3 | AI373676 | -3.88 | -1.46 |
| ZMYM2 | AL136621 | -3.87 | -1.34 |
| DKFZP586I1420 | AL050378 | -3.84 | -1.28 |
| PDS5B | NM_015928 | -3.78 | -1.37 |
| KIAA0776 | AL132776 | -3.78 | -1.32 |
| ARID4B | NM_016374 | -3.76 | -1.37 |
| LARP7 | AA160181 | -3.75 | -1.39 |
| DNAJB14 | NM_024920 | -3.67 | -1.38 |
| MTUS1 | BE552421 | -3.66 | -1.40 |
| NOL8 | NM_017948 | -3.66 | -1.20 |
| SREK1 | AI810380 | -3.65 | -1.46 |
| ZNF12 | NM_016265 | -3.64 | -1.68 |
| HERC2P9 | AB002391 | -3.63 | -1.24 |
| DLGAP5 | NM_014750 | -3.61 | -1.45 |
| ECT2 | NM_018098 | -3.54 | -1.76 |
| SEPP1 | NM_005410 | -3.50 | -1.47 |
| ACAP2 | D26069 | -3.50 | -1.41 |
| MANEA | NM_024641 | -3.50 | -1.25 |
| KIAA0528 | AB011100 | -3.49 | -1.52 |
| LOC100132247 | BG256504 | -3.45 | -1.34 |
| LOC613037 | BG256504 | -3.44 | -1.34 |
| DENND4A | BE268538 | -3.44 | -2.70 |
| L3MBTL1 | U89358 | -3.44 | -1.56 |
| NRIP1 | AI824012 | -3.43 | -1.31 |
| NPIPL3 | BG256504 | -3.42 | -1.34 |
| NFIB | AI186739 | -3.41 | -2.17 |
| NFIB | BC001283 | -3.41 | -1.73 |
| NFIB | AI186739 | -3.41 | -1.67 |
| NFIB | U70862 | -3.41 | -1.58 |
| NFIB | BG478428 | -3.41 | -1.41 |
| NFIB | AI700518 | -3.41 | -1.34 |
| RECQL | BC001052 | -3.41 | -1.37 |
| BPTF | NM_004459 | -3.40 | -1.32 |
| BPTF | AB032251 | -3.40 | -1.26 |
| CFH | X56210 | -3.39 | -1.20 |
| RPS6KA5 | NM_004755 | -3.39 | -1.41 |
| ZDHHC17 | AI621223 | -3.38 | -1.43 |
| RMI1 | NM_024945 | -3.36 | -1.67 |
| IL1RAPL1 | NM_014271 | -3.35 | -1.26 |
| ANKRD28 | AI081194 | -3.35 | -1.30 |
| ZFX | R51161 | -3.34 | -1.71 |
| SECISBP2L | D87445 | -3.33 | -1.52 |
| CEP57 | NM_014679 | -3.33 | -1.83 |
| CEP57 | AA918224 | -3.33 | -1.73 |
| CEP57 | AL525206 | -3.33 | -1.73 |
| CEP57 | AI123527 | -3.33 | -1.60 |
| CEP57 | BC001233 | -3.33 | -1.33 |
| SLC7A11 | AA488687 | -3.31 | -1.64 |
| SMG1 | U32581 | -3.31 | -1.50 |
| MDM1 | AW664850 | -3.30 | -1.27 |
| UTRN | NM_007124 | -3.29 | -1.76 |
| UTRN | NM_007124 | -3.29 | -1.52 |
| LRRC40 | NM_017768 | -3.27 | -1.20 |
| RSF1 | NM_016578 | -3.27 | -1.67 |
| ALMS1 | AW003635 | -3.25 | -1.35 |
| CCNT2 | AV681875 | -3.24 | -1.33 |
| CCNT2 | BE674119 | -3.24 | -1.24 |
| KIAA0586 | AB011158 | -3.23 | -1.38 |
| GABRR2 | NM_002043 | -3.22 | -1.30 |
| YES1 | NM_005433 | -3.22 | -1.39 |
| SLC25A36 | AI694452 | -3.21 | -1.31 |
| SLC25A36 | AI927944 | -3.21 | -1.24 |
| KRR1 | AI950314 | -3.21 | -1.23 |
| GUCY1B3 | W93728 | -3.20 | -1.80 |
| GUCY1B3 | AF020340 | -3.20 | -1.60 |
| MMRN1 | NM_007351 | -3.19 | -1.22 |
| ITPR2 | AA834576 | -3.19 | -2.38 |
| ITPR2 | NM_002223 | -3.19 | -1.96 |
| VPS13B | AI052003 | -3.16 | -1.61 |
| NCAPG | NM_022346 | -3.16 | -2.69 |
| DENND4C | NM_017925 | -3.15 | -1.25 |
| FAT4 | NM_024582 | -3.14 | -1.46 |
| DEK | NM_003472 | -3.14 | -1.27 |
| IFT88 | NM_006531 | -3.12 | -1.38 |
| IFI44 | NM_006417 | -3.10 | -1.55 |
| TPD52 | AA524023 | -3.09 | -1.54 |
| TPD52 | BG389015 | -3.09 | -1.33 |
| PCF11 | AB020631 | -3.09 | -1.36 |
| WSB1 | BF111821 | -3.08 | -1.58 |
| WSB1 | N24643 | -3.08 | -1.32 |
| CCDC91 | NM_018318 | -3.08 | -2.27 |
| KAT2B | AV727449 | -3.07 | -1.42 |
| DNAJB4 | BG252490 | -3.06 | -1.53 |
| DNAJB4 | NM_007034 | -3.06 | -1.32 |
| FAM13B | NM_016603 | -3.06 | -1.23 |
| ATP8A1 | AI769688 | -3.04 | -1.95 |
| ATP8A1 | AB013452 | -3.04 | -1.36 |
| CYP1B1 | NM_000104 | -3.04 | -2.35 |
| CYP1B1 | AU154504 | -3.04 | -1.60 |
| CYP1B1 | AU144855 | -3.04 | -1.47 |
| ZNF432 | NM_014650 | -3.02 | -1.20 |
| NBN | AK001017 | -3.00 | -1.40 |
| BTAF1 | AJ001017 | -3.00 | -1.20 |
| TIAF1 | NM_004740 | -2.99 | -1.33 |
| KCTD12 | AI718937 | -2.98 | -2.04 |
| KCTD12 | AA551075 | -2.98 | -1.85 |
| RFC1 | L14922 | -2.97 | -1.56 |
| THUMPD1 | AL134904 | -2.96 | -1.33 |
| TIA1 | H96549 | -2.92 | -1.42 |
| TIA1 | AL567227 | -2.92 | -1.40 |
| TIA1 | NM_022037 | -2.92 | -1.32 |
| TIA1 | AL046419 | -2.92 | -1.27 |
| ZFAND1 | NM_024699 | -2.92 | -1.46 |
| FAM13A | AK027138 | -2.91 | -1.44 |
| KIF3A | NM_007054 | -2.90 | -1.71 |
| TNFSF10 | AW474434 | -2.89 | -1.29 |
| MAN1A2 | AL157902 | -2.87 | -1.21 |
| KRIT1 | AL049325 | -2.87 | -1.72 |
| LARP4 | AI743740 | -2.85 | -1.32 |
| PDE4D | AF012074 | -2.83 | -1.82 |
| PDE4D | R40917 | -2.83 | -1.39 |
| TRIM66 | AW271713 | -2.83 | -1.27 |
| ZNF75D | AA209420 | -2.82 | -1.36 |
| ZNF75D | W90796 | -2.82 | -1.21 |
| FAM111A | NM_022074 | -2.81 | -1.36 |
| BCHE | NM_000055 | -2.79 | -1.45 |
| ATXN3 | AI888099 | -2.78 | -1.62 |
| NUDT4P1 | AF191654 | -2.77 | -1.41 |
| NUDT4P1 | NM_019094 | -2.77 | -1.20 |
| ZCCHC6 | NM_024617 | -2.77 | -1.26 |
| CEP170 | AA126789 | -2.76 | -1.23 |
| GIPC2 | NM_017655 | -2.75 | -1.49 |
| LIMCH1 | AK027231 | -2.75 | -1.86 |
| LIMCH1 | AB029025 | -2.75 | -1.74 |
| LIMCH1 | AK026815 | -2.75 | -1.70 |
| SRBD1 | NM_018079 | -2.75 | -1.26 |
| MGA | BE502432 | -2.75 | -1.22 |
| ZNF701 | NM_018260 | -2.75 | -1.57 |
| TRIL | AV724192 | -2.75 | -1.24 |
| RAB3GAP2 | AF255648 | -2.74 | -1.21 |
| RPGRIP1L | BF515597 | -2.73 | -1.25 |
| SSB | NM_003142 | -2.73 | -1.43 |
| MMP16 | NM_022564 | -2.72 | -1.20 |
| CD302 | NM_014880 | -2.72 | -1.82 |
| TTC35 | NM_014673 | -2.71 | -1.31 |
| AASS | AK023446 | -2.71 | -1.31 |
| PDS5A | AW991219 | -2.70 | -1.21 |
| TAX1BP1 | AI935415 | -2.67 | -1.59 |
| TWISTNB | AA400421 | -2.67 | -1.72 |
| ELK4 | AK024944 | -2.66 | -1.23 |
| TRIM52 | AA205660 | -2.65 | -1.25 |
| HERC4 | NM_015601 | -2.64 | -1.85 |
| INTS8 | NM_017864 | -2.63 | -1.23 |
| DYNC2LI1 | NM_016008 | -2.63 | -1.29 |
| SH3YL1 | NM_015677 | -2.59 | -1.44 |
| AHR | NM_001621 | -2.58 | -1.93 |
| REST | AV682285 | -2.57 | -1.44 |
| RFX7 | NM_022841 | -2.57 | -1.21 |
| NUDT4 | AW511135 | -2.57 | -1.62 |
| NUDT4 | AF191654 | -2.57 | -1.41 |
| NUDT4 | NM_019094 | -2.57 | -1.20 |
| SCRN3 | NM_024583 | -2.56 | -1.38 |
| PSIP1 | NM_004682 | -2.55 | -2.15 |
| IRAK3 | AL049435 | -2.55 | -1.75 |
| SNAPC1 | NM_003082 | -2.55 | -1.25 |
| KIF2A | BE872563 | -2.54 | -1.26 |
| TSPAN12 | NM_012338 | -2.54 | -1.52 |
| ARGLU1 | NM_018011 | -2.54 | -1.33 |
| SUZ12 | AI924660 | -2.54 | -1.33 |
| BRCC3 | NM_024332 | -2.53 | -2.02 |
| AP1AR | NM_018569 | -2.51 | -1.38 |
| ZNF510 | NM_014930 | -2.51 | -1.53 |
| MAP3K2 | AF239798 | -2.51 | -1.25 |
| TNPO1 | AI653355 | -2.50 | -1.29 |
| ZBTB6 | NM_006626 | -2.50 | -1.27 |
| EHBP1 | AB020710 | -2.50 | -1.44 |
| MTF2 | AL523380 | -2.48 | -1.26 |
| HIF1A | NM_001530 | -2.48 | -1.25 |
| TLK1 | Z25421 | -2.48 | -1.84 |
| NR1D2 | N32859 | -2.47 | -1.59 |
| ZNF323 | AW086021 | -2.47 | -1.36 |
| NUCB2 | NM_005013 | -2.47 | -1.22 |
| SLC30A9 | NM_006345 | -2.47 | -1.26 |
| RNMT | AB020966 | -2.47 | -1.44 |
| C1GALT1 | NM_020156 | -2.46 | -1.39 |
| DCUN1D4 | D87466 | -2.45 | -1.45 |
| IPW | AW770748 | -2.45 | -1.38 |
| IPW | AI672541 | -2.45 | -1.29 |
| PREPL | AB007896 | -2.45 | -1.36 |
| TGFBR3 | NM_003243 | -2.45 | -1.68 |
| PEX1 | AC000064 | -2.44 | -1.23 |
| SATB1 | NM_002971 | -2.44 | -1.82 |
| ARL4A | NM_005738 | -2.43 | -3.74 |
| OSBPL9 | NM_024586 | -2.43 | -1.20 |
| SPAST | AB029006 | -2.42 | -1.65 |
| TRIM33 | AA205593 | -2.42 | -1.72 |
| RAD21 | BG289967 | -2.42 | -1.21 |
| KIN | NM_012311 | -2.41 | -1.31 |
| AGGF1 | NM_018046 | -2.41 | -1.27 |
| LIN7C | AF090900 | -2.40 | -1.39 |
| HIBCH | NM_014362 | -2.40 | -1.51 |
| HIBCH | AW000964 | -2.40 | -1.25 |
| C7orf58 | NM_024913 | -2.39 | -1.25 |
| TUBD1 | BC000258 | -2.38 | -1.46 |
| AMIGO2 | AC004010 | -2.38 | -1.31 |
| SMAD5 | AF010601 | -2.36 | -1.93 |
| FBXO5 | NM_012177 | -2.36 | -1.46 |
| CUL4B | AI650819 | -2.36 | -1.49 |
| CREB1 | M34356 | -2.36 | -1.37 |
| FLRT2 | NM_013231 | -2.36 | -1.78 |
| FLRT2 | AF169676 | -2.36 | -1.42 |
| FAM164A | NM_016010 | -2.36 | -1.27 |
| RBM39 | NM_004902 | -2.35 | -1.25 |
| PBK | NM_018492 | -2.35 | -1.25 |
| TRIB2 | BC002637 | -2.35 | -1.30 |
| KLHL24 | AW006750 | -2.35 | -1.80 |
| TOPORS | NM_005802 | -2.34 | -2.29 |
| BAZ1A | AA102574 | -2.34 | -1.61 |
| TAF2 | AK001618 | -2.34 | -1.26 |
| GATA3 | BC003070 | -2.33 | -1.50 |
| FAM45A | BE565675 | -2.33 | -1.24 |
| SNX2 | NM_003100 | -2.33 | -1.77 |
| SEC62 | U93239 | -2.33 | -1.29 |
| PLSCR4 | NM_020353 | -2.33 | -1.91 |
| DLG1 | AL121981 | -2.30 | -1.38 |
| PELI2 | NM_021255 | -2.30 | -1.32 |
| DIAPH2 | L22650 | -2.29 | -1.37 |
| PBX1 | AL049381 | -2.29 | -1.70 |
| RABGGTB | U49245 | -2.29 | -1.34 |
| APPBP2 | AA046411 | -2.28 | -1.54 |
| APPBP2 | NM_006380 | -2.28 | -1.30 |
| HMGN3 | AF274949 | -2.28 | -1.35 |
| BRWD1 | AJ002572 | -2.28 | -1.21 |
| SCML1 | NM_006746 | -2.28 | -1.27 |
| ZBTB20 | NM_015642 | -2.28 | -1.24 |
| PCMTD2 | AB028973 | -2.28 | -1.35 |
| TFPI | BF109662 | -2.27 | -1.84 |
| TFPI | AF021834 | -2.27 | -1.61 |
| TFPI | J03225 | -2.27 | -1.45 |
| TFPI | BF511231 | -2.27 | -1.32 |
| ZMYM4 | AI650586 | -2.27 | -1.23 |
| SOS1 | L13857 | -2.27 | -1.34 |
| ZNF331 | NM_018555 | -2.26 | -1.29 |
| DBT | M27093 | -2.26 | -1.60 |
| VAV3 | NM_006113 | -2.26 | -1.49 |
| AZI2 | NM_022461 | -2.26 | -1.29 |
| SLC30A1 | AI972416 | -2.25 | -1.39 |
| MPHOSPH8 | BC003542 | -2.25 | -1.24 |
| FGF2 | NM_002006 | -2.25 | -1.95 |
| GPATCH2 | NM_018040 | -2.25 | -1.28 |
| PHF14 | NM_014660 | -2.23 | -1.23 |
| E2F8 | NM_024680 | -2.22 | -1.52 |
| MID1 | BE967532 | -2.22 | -1.65 |
| C12orf11 | AF274950 | -2.21 | -1.30 |
| ITSN2 | U61167 | -2.21 | -2.54 |
| CDC27 | N21397 | -2.20 | -1.24 |
| METTL7A | NM_014033 | -2.20 | -2.55 |
| VCAN | BF218922 | -2.19 | -1.37 |
| VCAN | NM_004385 | -2.19 | -1.34 |
| PPAT | U00238 | -2.19 | -1.30 |
| ST3GAL5 | NM_003896 | -2.19 | -1.49 |
| HMGCS1 | BG035985 | -2.19 | -1.30 |
| UBR5 | AF006010 | -2.18 | -1.37 |
| LEPR | U50748 | -2.18 | -1.20 |
| NFYB | AU151875 | -2.18 | -1.54 |
| GIMAP4 | NM_018326 | -2.17 | -1.40 |
| USP24 | BF444943 | -2.17 | -1.33 |
| FXR1 | AI990766 | -2.16 | -1.30 |
| FXR1 | BG025078 | -2.16 | -1.21 |
| PIGA | NM_002641 | -2.16 | -1.83 |
| IFI16 | NM_005531 | -2.16 | -1.43 |
| IFI16 | BG256677 | -2.16 | -1.34 |
| IFI16 | AF208043 | -2.16 | -1.28 |
| KCTD3 | NM_016121 | -2.16 | -1.52 |
| ASF1A | NM_014034 | -2.16 | -1.31 |
| CPD | D85390 | -2.16 | -1.32 |
| MIA3 | D87742 | -2.16 | -1.39 |
| LMO7 | NM_005358 | -2.14 | -2.35 |
| SDPR | NM_004657 | -2.14 | -1.60 |
| SYNJ1 | NM_003895 | -2.13 | -1.26 |
| EIF5B | AB018284 | -2.13 | -1.40 |
| FBXO3 | NM_012175 | -2.12 | -1.20 |
| PDCD4 | NM_014456 | -2.11 | -1.93 |
| PDCD4 | N92498 | -2.11 | -1.92 |
| PDCD4 | AI185160 | -2.11 | -1.76 |
| PDCD4 | NM_014456 | -2.11 | -1.58 |
| CLCN3 | AA902971 | -2.11 | -1.50 |
| PPP1CB | W67887 | -2.10 | -1.64 |
| MUDENG | NM_018229 | -2.10 | -1.21 |
| ECHDC1 | NM_018479 | -2.10 | -1.47 |
| ZMYM5 | NM_014242 | -2.09 | -1.22 |
| ZMYM5 | AI522311 | -2.09 | -1.21 |
| C14orf138 | NM_024558 | -2.09 | -1.48 |
| IDE | N22903 | -2.08 | -1.28 |
| FRY | NM_023037 | -2.07 | -1.90 |
| FRY | W58342 | -2.07 | -1.28 |
| ALG13 | NM_024810 | -2.06 | -1.22 |
| C14orf135 | NM_022495 | -2.06 | -1.34 |
| CRBN | NM_016302 | -2.06 | -1.42 |
| CD46 | AL570661 | -2.06 | -1.33 |
| BNIP2 | BC002461 | -2.05 | -1.25 |
| EMCN | NM_016242 | -2.04 | -1.21 |
| TUG1 | N37081 | -2.03 | -1.21 |
| FAM45B | BE565675 | -2.03 | -1.24 |
| RNF125 | NM_017831 | -2.03 | -1.24 |
| KRAS | BF673699 | -2.03 | -1.25 |
| CDKN3 | AF213033 | -2.02 | -1.25 |
| SLMO2 | NM_016045 | -2.02 | -1.37 |
| FBXW7 | NM_018315 | -2.02 | -1.20 |
| MALT1 | AF070528 | -2.02 | -2.61 |
| FRMD4B | AU145019 | -2.02 | -1.25 |
| CLIC2 | AI768628 | -2.02 | -1.95 |
| GUF1 | NM_021927 | -2.02 | -1.68 |
| INPP5F | NM_014937 | -2.01 | -1.57 |
| NPIP | AC002045 | -2.01 | -1.47 |
| ATP6V1C1 | AW024925 | -2.01 | -1.26 |
| HERC5 | NM_016323 | -2.00 | -1.93 |
|  |  |  |  |
| Different between the datasets (205) | | | |
| Gene Symbol | Representative Public ID | RNA Seq Fold Change | Microarray Fold Change |
| ST20 | AF249277 | 2.98 | -1.40 |
| UNC5B | AL049370 | 2.84 | -1.44 |
| UNC5B | AA127885 | 2.84 | -1.34 |
| ABCG1 | NM_004915 | 2.63 | -1.24 |
| TNFAIP3 | NM_006290 | 2.53 | -1.34 |
| PLAUR | X74039 | 2.15 | -1.39 |
| WDR52 | NM_018338 | -13.76 | 1.22 |
| BRCA2 | X95152 | -9.46 | 1.67 |
| ZNF107 | NM_016220 | -7.41 | 1.31 |
| ATM | U82828 | -6.84 | 1.42 |
| TTC37 | BE566023 | -6.40 | 1.22 |
| SPON1 | AB018305 | -5.76 | 1.33 |
| GUCY1A3 | AI719730 | -5.71 | 1.62 |
| TPR | BF110993 | -5.60 | 1.44 |
| SMC5 | AB011166 | -5.20 | 2.33 |
| KIAA1033 | AK001657 | -5.07 | 1.24 |
| LOC100272228 | U66046 | -5.07 | 1.85 |
| SACS | AI932370 | -4.92 | 1.33 |
| DST | BC004912 | -4.89 | 1.28 |
| DEPDC1 | NM_017779 | -4.83 | 1.29 |
| THOC2 | BE543527 | -4.77 | 1.33 |
| SRSF2IP | AW084759 | -4.75 | 1.29 |
| MEG3 | AI950273 | -4.70 | 1.79 |
| PPIG | AI638762 | -4.64 | 1.22 |
| CCNE2 | NM_004702 | -4.56 | 1.51 |
| AKAP11 | AK002166 | -4.46 | 1.30 |
| CXADR | NM_001338 | -4.33 | 1.69 |
| NEAT1 | AU134977 | -4.30 | 1.34 |
| TMF1 | BF593908 | -4.19 | 1.36 |
| CUL5 | NM_003478 | -4.00 | 1.79 |
| TTK | NM_003318 | -3.95 | 1.31 |
| RASSF9 | AF056209 | -3.93 | 1.41 |
| ROCK2 | AL049383 | -3.86 | 1.24 |
| KIF5B | NM_004521 | -3.82 | 1.30 |
| NPAT | D83243 | -3.81 | 1.25 |
| GABPA | D13318 | -3.80 | 1.37 |
| SLC5A3 | AK024896 | -3.80 | 1.26 |
| SLC5A3 | BF982927 | -3.80 | 1.47 |
| SLC5A3 | AI867198 | -3.80 | 1.65 |
| RORA | U04897 | -3.72 | 1.50 |
| WDHD1 | NM_007086 | -3.69 | 1.38 |
| PKN2 | AF118089 | -3.63 | 1.51 |
| MYO9A | NM_006901 | -3.59 | 1.28 |
| MAP4K3 | NM_003618 | -3.59 | 1.32 |
| PPP1R12A | BE737620 | -3.56 | 1.47 |
| LGR4 | NM_018490 | -3.45 | 1.27 |
| ZNF267 | AU150728 | -3.44 | 1.24 |
| L3MBTL1 | Z98752 | -3.44 | 1.22 |
| CSAD | NM_015989 | -3.42 | 1.83 |
| ZNF136 | NM_003437 | -3.41 | 1.36 |
| TRIM23 | AF230399 | -3.39 | 1.33 |
| ZFX | NM_003411 | -3.34 | 1.74 |
| MOSPD2 | AW469184 | -3.30 | 1.20 |
| LRRC40 | AL390149 | -3.27 | 1.27 |
| COPS2 | AA496247 | -3.25 | 1.61 |
| GCFC1 | NM_016631 | -3.25 | 1.48 |
| ALMS1 | AB002326 | -3.25 | 1.56 |
| KIAA0586 | NM_014749 | -3.23 | 1.26 |
| KIAA1598 | AU157109 | -3.21 | 1.30 |
| CDC42BPA | NM_003607 | -3.16 | 1.20 |
| CDC42BPA | AK027000 | -3.16 | 1.60 |
| WRN | NM_000553 | -3.14 | 1.33 |
| ARHGAP5 | AW044631 | -3.12 | 1.31 |
| ZNF816 | BG290532 | -3.12 | 1.85 |
| WSB1 | AA521269 | -3.08 | 1.30 |
| GIN1 | NM_017676 | -3.07 | 1.41 |
| FAM63B | AU121431 | -3.03 | 1.30 |
| IFT81 | NM_014055 | -3.01 | 1.44 |
| SF3B1 | AW003030 | -3.01 | 1.36 |
| SSX2IP | AK001710 | -2.99 | 1.27 |
| SSX2IP | AW136988 | -2.99 | 1.30 |
| PLCB1 | AL049593 | -2.97 | 1.24 |
| RFC1 | NM_002913 | -2.97 | 1.31 |
| POSTN | D13665 | -2.96 | 1.60 |
| SERINC4 | NM_025165 | -2.94 | 1.73 |
| OPA1 | AA209332 | -2.94 | 1.20 |
| ENPP4 | NM_014936 | -2.93 | 1.62 |
| DMD | NM_004010 | -2.92 | 1.22 |
| STAG1 | AI126490 | -2.91 | 1.29 |
| FGD6 | NM_018351 | -2.90 | 1.36 |
| CENPQ | NM_018132 | -2.88 | 1.22 |
| DIS3 | NM_014953 | -2.87 | 1.50 |
| LARP4 | AL050205 | -2.85 | 1.33 |
| PYROXD1 | NM_024854 | -2.84 | 1.24 |
| CAPRIN2 | NM_023925 | -2.84 | 1.58 |
| POLR3G | BF062139 | -2.83 | 2.00 |
| TAF1D | BC001972 | -2.82 | 1.26 |
| ATXN3 | NM_004993 | -2.78 | 1.77 |
| RAB3GAP2 | BF240652 | -2.74 | 1.52 |
| HCFC2 | NM_013320 | -2.73 | 1.20 |
| THOC1 | NM_005131 | -2.72 | 1.21 |
| MMP16 | AB009303 | -2.72 | 1.58 |
| ZNF468 | BE541042 | -2.71 | 1.31 |
| ZNF286A | NM_020652 | -2.71 | 1.20 |
| SIKE1 | NM_025073 | -2.70 | 1.28 |
| ZNF767 | NM_024910 | -2.70 | 1.30 |
| QSER1 | NM_024774 | -2.69 | 1.60 |
| PHC3 | AK023029 | -2.67 | 1.49 |
| C12orf48 | NM_017915 | -2.66 | 1.32 |
| HSP90AA1 | AI962933 | -2.66 | 1.27 |
| SCYL2 | NM_017988 | -2.64 | 1.20 |
| PRPF40A | Z78308 | -2.63 | 1.20 |
| NCAM2 | NM_004540 | -2.62 | 1.78 |
| ABCE1 | AI002002 | -2.60 | 1.21 |
| ABCE1 | NM_002940 | -2.60 | 1.28 |
| GP1BA | NM_000173 | -2.56 | 1.38 |
| ELOVL6 | NM_024090 | -2.56 | 1.21 |
| SEMA3C | AI962897 | -2.56 | 1.29 |
| PSIP1 | AF098482 | -2.55 | 1.30 |
| SCAPER | AW139369 | -2.55 | 1.58 |
| BCAT1 | AI652662 | -2.51 | 1.35 |
| CLOCK | NM_004898 | -2.50 | 1.35 |
| TNPO1 | AW161626 | -2.50 | 1.24 |
| MTF2 | AI566096 | -2.48 | 1.22 |
| MTF2 | AF072814 | -2.48 | 1.24 |
| MTF2 | BG033764 | -2.48 | 1.49 |
| BBS10 | NM_024685 | -2.47 | 1.42 |
| PAN2 | NM_014871 | -2.47 | 1.23 |
| KLHL4 | BF215673 | -2.47 | 1.40 |
| DCLRE1C | AK022922 | -2.44 | 1.27 |
| SPAST | NM_014946 | -2.42 | 1.34 |
| TRIM33 | AI967961 | -2.42 | 1.39 |
| TRIM33 | AF220137 | -2.42 | 1.45 |
| TRDMT1 | AJ223333 | -2.42 | 1.37 |
| FILIP1L | NM_014890 | -2.41 | 2.17 |
| GVINP1 | NM_025006 | -2.40 | 1.66 |
| CTNNAL1 | NM_003798 | -2.40 | 1.35 |
| ZNF24 | BC003566 | -2.40 | 1.20 |
| ZNF24 | NM_006965 | -2.40 | 1.24 |
| ZNF24 | AU144066 | -2.40 | 1.25 |
| TDRD3 | NM_030794 | -2.38 | 1.24 |
| KIAA0232 | D86985 | -2.38 | 1.22 |
| ZNF192 | NM_006298 | -2.37 | 1.86 |
| EPRS | AI142677 | -2.37 | 1.36 |
| MYRIP | AL050090 | -2.37 | 1.35 |
| ERI2 | N64622 | -2.36 | 1.24 |
| PHACTR2 | NM_014721 | -2.34 | 1.22 |
| BAZ1A | NM_013448 | -2.34 | 1.27 |
| SYNJ2BP | NM_018373 | -2.33 | 1.32 |
| ADAM10 | AU135154 | -2.33 | 1.36 |
| TRIM45 | NM_025188 | -2.33 | 1.40 |
| ANXA1 | NM_000700 | -2.30 | 2.09 |
| NNAT | NM_005386 | -2.30 | 1.48 |
| ALCAM | AA156721 | -2.29 | 1.72 |
| DIAPH2 | NM_007309 | -2.29 | 1.33 |
| SREK1IP1 | AW408767 | -2.29 | 1.50 |
| RABGGTB | U49245 | -2.29 | 1.28 |
| REL | NM_002908 | -2.28 | 1.22 |
| MGEA5 | AU144791 | -2.28 | 1.35 |
| AGPS | NM_003659 | -2.28 | 1.29 |
| SNAI2 | AI572079 | -2.27 | 1.24 |
| NOC3L | NM_022451 | -2.27 | 1.27 |
| MBD5 | NM_018328 | -2.24 | 1.22 |
| HSPH1 | BG403660 | -2.23 | 1.20 |
| NEK3 | Z25434 | -2.23 | 1.38 |
| C14orf45 | NM_025057 | -2.22 | 1.26 |
| SYT1 | AV731490 | -2.21 | 2.27 |
| RRP15 | NM_016052 | -2.20 | 1.38 |
| EPS8 | NM_004447 | -2.20 | 1.65 |
| FNDC3A | NM_014923 | -2.20 | 1.21 |
| METTL7A | BC004492 | -2.20 | 1.24 |
| TOX | AI961231 | -2.19 | 1.31 |
| SLC35A3 | BC005136 | -2.19 | 1.83 |
| GLMN | NM_007070 | -2.18 | 1.81 |
| RBM25 | BE466128 | -2.18 | 1.21 |
| UCHL5 | NM_016017 | -2.18 | 1.50 |
| WBP4 | NM_007187 | -2.17 | 1.29 |
| CTAGE1 | NM_022663 | -2.17 | 1.25 |
| SMARCA1 | M88163 | -2.16 | 1.22 |
| TLR1 | AL050262 | -2.16 | 1.23 |
| RNF19A | NM_015435 | -2.14 | 1.24 |
| SOS2 | L20686 | -2.13 | 1.22 |
| EIF5B | BG261322 | -2.13 | 1.39 |
| ZNF197 | NM_006991 | -2.12 | 1.37 |
| SLC26A2 | AI025519 | -2.11 | 1.23 |
| RFC4 | NM_002916 | -2.11 | 1.30 |
| MITF | NM_000248 | -2.10 | 1.35 |
| TNKS2 | NM_025235 | -2.10 | 1.26 |
| MDFIC | AF054589 | -2.10 | 1.20 |
| ERCC6 | NM_000124 | -2.10 | 1.30 |
| IGF2BP3 | AU160004 | -2.09 | 1.47 |
| RC3H2 | NM_018835 | -2.08 | 1.32 |
| MAK16 | AF251062 | -2.08 | 1.39 |
| ARHGEF6 | D25304 | -2.08 | 1.20 |
| ACVR2A | NM_001616 | -2.08 | 1.36 |
| HBS1L | AK024258 | -2.07 | 1.23 |
| TROVE2 | AL538601 | -2.07 | 1.50 |
| PIGK | AF022913 | -2.07 | 1.21 |
| RDX | AL137751 | -2.06 | 1.21 |
| RDX | AI057093 | -2.06 | 1.30 |
| RDX | NM_002906 | -2.06 | 1.48 |
| CAPN7 | BE349584 | -2.05 | 1.75 |
| ASPH | AF289489 | -2.05 | 1.49 |
| CCNJ | NM_019084 | -2.05 | 1.25 |
| PTPN11 | AF119855 | -2.05 | 1.26 |
| PTPN11 | L07527 | -2.05 | 1.27 |
| UAP1 | S73498 | -2.04 | 1.33 |
| LPP | BF221852 | -2.03 | 1.46 |
| STX17 | NM_017919 | -2.02 | 1.39 |
| APAF1 | AF248734 | -2.02 | 1.22 |
| FAS | X83493 | -2.02 | 1.41 |
| ZNF143 | AW162015 | -2.02 | 1.57 |
| MALT1 | NM_006785 | -2.02 | 1.21 |
| MEF2C | NM_002397 | -2.01 | 1.30 |
| MAP2 | U89330 | -2.00 | 1.70 |
