# Supplementary material for: RNA-seq Reveals Novel Transcriptome of Genes and Their Isoforms in Human Pulmonary Microvascular Endothelial Cells Treated with Thrombin
Source: PLoS One. 2012 Feb 16;7(2):e31229. doi: 10.1371/journal.pone.0031229 (PMC3281071; doi:10.1371/journal.pone.0031229)
Supplement: Table S8 — RNA Seq Data from 6 h thrombin-treated HMVEC vs Microarray Data from 5 h TNFα-treated HMVEC*. * The data from this study is compared to the study by Viemann et al.(24) in which gene expression profile was assayed by Affymetrix Human Genome U133A Array. ** ND, not detected either in control group or TNF alpha treated group. (DOCX) [file pone.0031229.s008.docx]

Table S8. RNA Seq Data from 6 h thrombin-treated HMVEC vs Microarray Data from 5 h TNFα-treated HMVEC *

| Up-regulated in both datasets (49) | | | |
| --- | --- | --- | --- |
| Gene Symbol | Representative Public ID | RNA Seq Fold Change | Microarray Fold Change |
| VCAM1 | NM_001078 | 4.75 | 11.00 |
| IL1B | M15330 | 4.25 | 3.99 |
| CD83 | NM_004233 | 3.92 | 2.17 |
| TMEM158 | BF062629 | 3.64 | 1.88 |
| CXCR7 | AI817041 | 3.43 | 1.70 |
| JAG1 | U61276 | 3.38 | 1.64 |
| JAG1 | U77914 | 3.38 | 1.66 |
| JAG1 | U73936 | 3.38 | 1.79 |
| ICAM1 | AA284705 | 3.29 | 7.70 |
| ICAM1 | AI608725 | 3.29 | 9.78 |
| ICAM1 | NM_000201 | 3.29 | 11.53 |
| F3 | NM_001993 | 3.16 | 1.33 |
| TNFRSF9 | NM_001561 | 2.88 | 2.28 |
| TNFAIP2 | NM_006291 | 2.83 | 7.58 |
| ABTB2 | AL050374 | 2.82 | 1.47 |
| IL8 | NM_000584 | 2.78 | 6.55 |
| IL8 | AF043337 | 2.78 | 11.10 |
| HBEGF | M60278 | 2.65 | 1.33 |
| HBEGF | NM_001945 | 2.65 | 1.48 |
| ABCG1 | U34919 | 2.63 | 1.54 |
| ABCG1 | NM_004915 | 2.63 | 2.07 |
| TNFAIP3 | AI738896 | 2.53 | 7.20 |
| TNFAIP3 | NM_006290 | 2.53 | 8.03 |
| IRF1 | NM_002198 | 2.46 | 2.18 |
| LITAF | AB034747 | 2.43 | 1.41 |
| NKX3-1 | AF247704 | 2.41 | 2.63 |
| FST | NM_006350 | 2.36 | 2.49 |
| FST | NM_013409 | 2.36 | 2.68 |
| SOD2 | X15132 | 2.35 | 6.06 |
| SOD2 | W46388 | 2.35 | 7.74 |
| RELB | NM_006509 | 2.33 | 3.32 |
| MT1G | NM_005950 | 2.31 | 1.41 |
| CDCP1 | NM_022842 | 2.30 | 1.54 |
| TNIP1 | NM_006058 | 2.28 | 5.02 |
| IL6 | NM_000600 | 2.27 | 1.52 |
| JUNB | NM_002229 | 2.24 | 2.14 |
| GPR176 | NM_007223 | 2.22 | 1.51 |
| TFPI2 | L27624 | 2.21 | 1.78 |
| TFPI2 | AL574096 | 2.21 | 1.90 |
| ARHGEF40 | R42449 | 2.17 | 1.68 |
| PLAUR | X74039 | 2.15 | 1.55 |
| PLAUR | U08839 | 2.15 | 1.86 |
| PLAUR | AY029180 | 2.15 | 2.22 |
| PDGFB | AU150748 | 2.06 | 1.34 |
| RGS20 | AF074979 | 2.05 | 1.62 |
| CSF1 | M37435 | 2.03 | 2.58 |
| NFKB2 | NM_002502 | 2.02 | 5.88 |
| GBP1 | NM_002053 | 2.01 | 2.09 |
| GBP1 | BC002666 | 2.01 | 2.57 |
| TNFAIP2 | AI862445 | 2.83 | ND |
| EFNB1 | NM_004429 | 2.01 | ND |
| STAT5A | NM_003152 | 2.12 | ND |
| PDGFB | NM_002608 | 2.06 | ND |
| ISG20 | NM_002201 | 2.42 | ND |
| IL1B | NM_000576 | 4.25 | ND |
| LIF | NM_002309 | 5.52 | ND |
| CCL20 | NM_004591 | 5.95 | ND |
| TRAF1 | NM_005658 | 7.96 | ND |
| SELE | NM_000450 | 5.13 | ND |
| SLC7A2 | NM_003046 | 2.93 | ND |
| CXCL3 | NM_002090 | 2.17 | ND |
| NFKB2 | BC002844 | 2.02 | ND |
| SOD2 | AL050388 | 2.35 | ND |
| ISG20 | U88964 | 2.42 | ND |
|  |  |  |  |
| Down-regulated in both datasets (264) | | | |
| ZNF266 | AA868898 | -2.00 | -1.34 |
| ATP6V1C1 | BF034973 | -2.01 | -1.43 |
| FAM8A1 | NM_016255 | -2.01 | -1.75 |
| GUF1 | NM_021927 | -2.02 | -1.37 |
| MALT1 | NM_006785 | -2.02 | -1.30 |
| ZNF652 | NM_014897 | -2.03 | -1.75 |
| LASS6 | AI658534 | -2.03 | -1.34 |
| MAP3K1 | AA361361 | -2.03 | -1.34 |
| DPYD | NM_000110 | -2.04 | -1.63 |
| C6orf211 | NM_024573 | -2.05 | -1.38 |
| FRY | W58342 | -2.07 | -1.60 |
| IDE | AA918442 | -2.08 | -1.33 |
| INPP4B | NM_003866 | -2.08 | -1.45 |
| GPD2 | U36310 | -2.08 | -1.34 |
| ZMYM5 | NM_014242 | -2.09 | -1.94 |
| ZMYM5 | AI522311 | -2.09 | -1.44 |
| MDFIC | AF054589 | -2.10 | -1.42 |
| MDFIC | BE910600 | -2.10 | -1.30 |
| ASH1L | NM_018489 | -2.10 | -1.35 |
| CLCN3 | AI760629 | -2.11 | -1.36 |
| CLCN3 | NM_001829 | -2.11 | -1.40 |
| PDCD4 | NM_014456 | -2.11 | -1.43 |
| PDCD4 | NM_014456 | -2.11 | -1.40 |
| PDCD4 | N92498 | -2.11 | -1.73 |
| PDCD4 | AI185160 | -2.11 | -1.33 |
| FBXO3 | NM_012175 | -2.12 | -1.53 |
| SOS2 | AI628605 | -2.13 | -1.32 |
| SOS2 | AI276593 | -2.13 | -1.36 |
| SYNJ1 | NM_003895 | -2.13 | -1.48 |
| SYNJ1 | AB020717 | -2.13 | -1.57 |
| SEL1L | AF052059 | -2.13 | -1.32 |
| TTBK2 | AW294686 | -2.13 | -1.97 |
| SDPR | NM_004657 | -2.14 | -2.95 |
| PRKCI | L18964 | -2.14 | -1.42 |
| MIA3 | AK025122 | -2.16 | -1.46 |
| MIA3 | D87742 | -2.16 | -1.47 |
| ASF1A | NM_014034 | -2.16 | -1.58 |
| ASF1A | AB028628 | -2.16 | -1.66 |
| FXR1 | AI990766 | -2.16 | -1.34 |
| NFYB | AI804118 | -2.18 | -1.49 |
| NFYB | AU151875 | -2.18 | -2.05 |
| NFYB | NM_006166 | -2.18 | -2.80 |
| LEPR | U52913 | -2.18 | -1.45 |
| LEPR | U52914 | -2.18 | -1.42 |
| LEPR | U66495 | -2.18 | -1.39 |
| GLMN | NM_007070 | -2.18 | -1.30 |
| ST3GAL5 | NM_003896 | -2.19 | -1.45 |
| TOX | AI961231 | -2.19 | -2.20 |
| VCAN | D32039 | -2.19 | -1.57 |
| RUFY3 | AF112221 | -2.19 | -1.30 |
| METTL7A | NM_014033 | -2.20 | -1.60 |
| METTL7A | AF113007 | -2.20 | -1.32 |
| PPWD1 | AK025679 | -2.20 | -1.41 |
| CDC27 | N21397 | -2.20 | -1.55 |
| EPS8 | NM_004447 | -2.20 | -1.44 |
| XRCC4 | NM_022406 | -2.23 | -1.65 |
| DBT | J03208 | -2.26 | -1.46 |
| SOS1 | L13857 | -2.27 | -1.41 |
| SOS1 | AA700167 | -2.27 | -1.32 |
| ZMYM4 | AA521508 | -2.27 | -1.43 |
| PCMTD2 | AB028973 | -2.28 | -1.71 |
| ZBTB20 | NM_015642 | -2.28 | -1.36 |
| C5orf44 | NM_024941 | -2.28 | -1.39 |
| ABCD3 | NM_002858 | -2.28 | -1.34 |
| APPBP2 | AV681579 | -2.28 | -1.31 |
| APPBP2 | AA046411 | -2.28 | -1.47 |
| APPBP2 | NM_006380 | -2.28 | -1.33 |
| DLG1 | AL121981 | -2.30 | -1.30 |
| TLR3 | NM_003265 | -2.31 | -1.50 |
| ADAM10 | NM_001110 | -2.33 | -1.32 |
| SNX2 | AF043453 | -2.33 | -1.48 |
| SNX2 | NM_003100 | -2.33 | -1.42 |
| ZSCAN12 | NM_014724 | -2.33 | -1.71 |
| ORC3 | AF125507 | -2.34 | -1.52 |
| PHACTR2 | AA551142 | -2.34 | -1.39 |
| PHACTR2 | NM_014721 | -2.34 | -1.44 |
| CBR4 | AL049442 | -2.34 | -1.34 |
| UBXN2B | AI245523 | -2.34 | -1.52 |
| FLRT2 | NM_013231 | -2.36 | -2.05 |
| EPRS | AI142677 | -2.37 | -1.49 |
| CIR1 | U03644 | -2.37 | -1.31 |
| ZNF192 | NM_006298 | -2.37 | -1.46 |
| TUBD1 | BC000258 | -2.38 | -1.55 |
| ZNF187 | AV705032 | -2.39 | -1.35 |
| SLC35A1 | NM_006416 | -2.39 | -1.69 |
| ZNF24 | NM_006965 | -2.40 | -1.30 |
| AGGF1 | NM_018046 | -2.41 | -1.47 |
| TRIM33 | AF220137 | -2.42 | -1.37 |
| SPAST | NM_014946 | -2.42 | -1.35 |
| SPAST | AB029006 | -2.42 | -1.63 |
| VAMP4 | BC005974 | -2.43 | -1.58 |
| OSBPL9 | NM_024586 | -2.43 | -1.33 |
| PEX1 | NM_000466 | -2.44 | -1.48 |
| PEX1 | AC000064 | -2.44 | -1.50 |
| PREPL | AU154782 | -2.45 | -1.30 |
| KLHL4 | BF215673 | -2.47 | -1.57 |
| UBXN4 | AU149908 | -2.47 | -1.46 |
| UBXN4 | AI927512 | -2.47 | -1.37 |
| UBXN4 | N29889 | -2.47 | -1.32 |
| PAN2 | NM_014871 | -2.47 | -1.38 |
| ZNF85 | NM_003429 | -2.47 | -1.52 |
| ATP7A | NM_000052 | -2.48 | -1.32 |
| IREB2 | AI204981 | -2.49 | -1.33 |
| EHBP1 | AB020710 | -2.50 | -1.50 |
| ZBTB6 | NM_006626 | -2.50 | -1.43 |
| MAP4K5 | Z25426 | -2.52 | -1.60 |
| BRCC3 | S72931 | -2.53 | -1.34 |
| BMPR2 | U25110 | -2.53 | -1.48 |
| PLCL1 | NM_006226 | -2.53 | -1.38 |
| MPDZ | NM_003829 | -2.54 | -1.40 |
| MPDZ | AA917899 | -2.54 | -1.33 |
| CEP192 | NM_018069 | -2.54 | -1.52 |
| KIF2A | BE872563 | -2.54 | -1.34 |
| SCAPER | AF119814 | -2.55 | -1.66 |
| MSH2 | U04045 | -2.56 | -1.39 |
| ELOVL6 | BC001305 | -2.56 | -1.31 |
| RFX7 | NM_022841 | -2.57 | -1.43 |
| SH3YL1 | NM_015677 | -2.59 | -1.44 |
| NCAM2 | NM_004540 | -2.62 | -1.47 |
| HERC4 | NM_015601 | -2.64 | -1.55 |
| NEK2 | Z25425 | -2.64 | -1.31 |
| MYNN | NM_018657 | -2.69 | -1.31 |
| NDC80 | NM_006101 | -2.70 | -1.43 |
| SIKE1 | NM_025073 | -2.70 | -1.41 |
| AASS | AF229180 | -2.71 | -1.35 |
| ERCC5 | NM_000123 | -2.73 | -2.61 |
| RPGRIP1L | BF515597 | -2.73 | -1.35 |
| PMS1 | BG434893 | -2.74 | -1.71 |
| RABEP1 | NM_004703 | -2.76 | -1.50 |
| KIF11 | NM_004523 | -2.78 | -1.41 |
| RBL1 | AL365505 | -2.79 | -1.30 |
| TRIM66 | AW271713 | -2.83 | -1.42 |
| PDE4D | R40917 | -2.83 | -1.31 |
| KRIT1 | AL049325 | -2.87 | -1.35 |
| KRIT1 | U90269 | -2.87 | -1.32 |
| MAN1A2 | H97940 | -2.87 | -1.30 |
| MAN1A2 | BE543064 | -2.87 | -1.37 |
| KPNA5 | NM_002269 | -2.89 | -1.37 |
| TWF1 | AW665024 | -2.90 | -1.38 |
| APPL1 | NM_012096 | -2.90 | -1.38 |
| TIA1 | H96549 | -2.92 | -1.55 |
| DNA2 | D42046 | -2.94 | -1.32 |
| WDR67 | AI017564 | -2.96 | -1.67 |
| CROT | NM_021151 | -2.96 | -1.61 |
| RAB11FIP2 | NM_014904 | -2.97 | -1.38 |
| KCTD12 | AA551075 | -2.98 | -1.45 |
| KCTD12 | AI718937 | -2.98 | -1.53 |
| SSX2IP | R52678 | -2.99 | -1.57 |
| RAD51AP1 | BE966146 | -3.01 | -1.42 |
| TMEM106B | NM_018374 | -3.03 | -1.55 |
| CYP1B1 | AU154504 | -3.04 | -1.46 |
| CYP1B1 | AU144855 | -3.04 | -1.35 |
| DNAJB4 | NM_007034 | -3.06 | -1.32 |
| CCDC91 | NM_018318 | -3.08 | -1.30 |
| WSB1 | AA521269 | -3.08 | -1.42 |
| PHTF2 | AC004990 | -3.10 | -1.38 |
| SRSF11 | NM_004768 | -3.10 | -1.38 |
| NEBL | AL157398 | -3.11 | -1.40 |
| WRN | NM_000553 | -3.14 | -1.55 |
| CAMSAP1L1 | AL110158 | -3.14 | -1.30 |
| NCAPG | NM_022346 | -3.16 | -1.53 |
| VPS13B | AI052003 | -3.16 | -1.43 |
| CDC42BPA | NM_014826 | -3.16 | -1.34 |
| OXR1 | NM_018002 | -3.17 | -1.62 |
| KRR1 | NM_007043 | -3.21 | -1.32 |
| GABRR2 | NM_002043 | -3.22 | -1.62 |
| ATAD2 | NM_014109 | -3.23 | -1.34 |
| ALMS1 | AW003635 | -3.25 | -1.45 |
| MDM1 | AW664850 | -3.30 | -1.30 |
| MRE11A | NM_005590 | -3.31 | -1.44 |
| CEP57 | AI123527 | -3.33 | -1.53 |
| CEP57 | AA918224 | -3.33 | -1.31 |
| CEP57 | AL525206 | -3.33 | -1.32 |
| CEP57 | NM_014679 | -3.33 | -1.36 |
| RYR3 | NM_001036 | -3.35 | -1.42 |
| TRIM23 | AF230399 | -3.39 | -1.37 |
| BPTF | AB032251 | -3.40 | -1.58 |
| RPAP3 | NM_024604 | -3.44 | -1.41 |
| LGR4 | NM_018490 | -3.45 | -1.58 |
| ZCCHC11 | AI049962 | -3.46 | -1.46 |
| RANBP6 | AI123233 | -3.48 | -1.41 |
| KIAA0528 | AB011100 | -3.49 | -1.77 |
| PLK4 | AL043646 | -3.54 | -1.64 |
| PLK4 | NM_014264 | -3.54 | -1.32 |
| DBF4 | NM_006716 | -3.58 | -1.34 |
| POT1 | BC002923 | -3.61 | -1.41 |
| POT1 | NM_015450 | -3.61 | -1.39 |
| DLGAP5 | NM_014750 | -3.61 | -1.35 |
| MTUS1 | AI695017 | -3.66 | -2.12 |
| MTUS1 | AL096842 | -3.66 | -2.03 |
| CLK1 | AI251890 | -3.68 | -1.37 |
| WDHD1 | AK001538 | -3.69 | -1.67 |
| KIAA0776 | AL132776 | -3.78 | -1.43 |
| KIAA0776 | AW298092 | -3.78 | -1.45 |
| CP110 | NM_014711 | -3.82 | -1.31 |
| ZMYM2 | AL136621 | -3.87 | -1.63 |
| SMC3 | BF795297 | -3.88 | -1.41 |
| SMC3 | AI373676 | -3.88 | -1.39 |
| TOP2A | AU159942 | -3.91 | -1.53 |
| TOP2A | AL561834 | -3.91 | -1.50 |
| FAM178A | AI655902 | -3.93 | -1.53 |
| FAM178A | AL133215 | -3.93 | -1.48 |
| CKAP2 | NM_018204 | -3.96 | -1.33 |
| ZNF451 | AB011148 | -3.99 | -1.47 |
| IBTK | AF235049 | -4.02 | -1.31 |
| RUNX1T1 | NM_004349 | -4.09 | -1.38 |
| EXOC5 | NM_006544 | -4.11 | -1.52 |
| ZNF675 | BF308250 | -4.12 | -1.74 |
| ZFP37 | NM_003408 | -4.13 | -1.51 |
| ZNF148 | L04282 | -4.13 | -1.31 |
| ZNF430 | NM_025189 | -4.14 | -1.37 |
| GOLGA6L9 | AI632181 | -4.18 | -1.36 |
| HAUS6 | NM_017645 | -4.19 | -1.40 |
| CCDC46 | AI979276 | -4.20 | -1.38 |
| GUSBP3 | X83300 | -4.20 | -1.59 |
| AGL | NM_000645 | -4.21 | -1.30 |
| NEK1 | AV700007 | -4.32 | -1.53 |
| CXADR | NM_001338 | -4.33 | -1.66 |
| USP34 | AL050376 | -4.44 | -1.53 |
| SDCCAG1 | NM_004713 | -4.45 | -1.41 |
| C3orf63 | N38985 | -4.49 | -1.52 |
| SMCHD1 | AA868754 | -4.49 | -1.31 |
| YTHDC2 | AL049305 | -4.51 | -1.32 |
| SNX13 | AA908770 | -4.60 | -1.39 |
| PPIG | U40763 | -4.64 | -1.60 |
| SRSF2IP | NM_004719 | -4.75 | -1.57 |
| SRSF2IP | AW084759 | -4.75 | -1.54 |
| SRSF2IP | AI984932 | -4.75 | -1.53 |
| PHF3 | AI949220 | -4.79 | -1.49 |
| PHF3 | NM_015153 | -4.79 | -1.40 |
| CENPC1 | NM_001812 | -4.80 | -1.49 |
| GOLGA6L5 | AI632181 | -4.82 | -1.36 |
| APC | AI375486 | -4.86 | -1.53 |
| APC | M74088 | -4.86 | -1.42 |
| ZNF43 | NM_003423 | -4.92 | -1.48 |
| TTC3 | AI652848 | -5.02 | -1.34 |
| ZFYVE16 | NM_014733 | -5.13 | -1.30 |
| SMC4 | NM_005496 | -5.14 | -1.39 |
| PCM1 | NM_006197 | -5.15 | -1.34 |
| PHIP | BF224151 | -5.16 | -1.67 |
| PHIP | BG545769 | -5.16 | -1.41 |
| ZNF33B | BG429214 | -5.20 | -1.39 |
| MPHOSPH9 | NM_022782 | -5.34 | -1.54 |
| MPHOSPH9 | X98258 | -5.34 | -1.42 |
| SFRS18 | AW081113 | -5.43 | -1.35 |
| KIAA1109 | AB029032 | -5.49 | -1.35 |
| RB1CC1 | BG402105 | -5.58 | -1.70 |
| BAZ2B | NM_013450 | -5.61 | -1.59 |
| HMMR | NM_012485 | -5.71 | -1.35 |
| HMMR | U29343 | -5.71 | -1.60 |
| OCLM | NM_022375 | -5.74 | -1.31 |
| HLTF | AI760760 | -6.17 | -1.58 |
| PRKACB | NM_002731 | -6.23 | -1.96 |
| ATM | U82828 | -6.84 | -1.85 |
| ATRX | AI650257 | -7.30 | -1.43 |
| ATRX | U09820 | -7.30 | -1.45 |
| ATRX | U72937 | -7.30 | -1.44 |
| KIAA1009 | NM_014895 | -8.35 | -1.42 |
| AKAP9 | AB019691 | -8.51 | -1.82 |
| VPS13A | AI186145 | -9.00 | -1.32 |
| BRCA2 | NM_000059 | -9.46 | -1.49 |
| BRCA2 | X95152 | -9.46 | -1.35 |
| LOC220594 | AW194543 | -10.74 | -1.30 |
| ZNF117 | NM_015852 | -11.49 | -1.54 |
| UEVLD | NM_018314 | -2.01 | ND^**^ |
| ZNF143 | AW162015 | -2.02 | ND |
| C13orf34 | NM_024808 | -2.02 | ND |
| APAF1 | AF248734 | -2.02 | ND |
| ZMAT3 | NM_022470 | -2.02 | ND |
| FAM45B | BE565675 | -2.03 | ND |
| TUG1 | AK000749 | -2.03 | ND |
| ADAMTSL4 | NM_025008 | -2.03 | ND |
| SEC61A2 | NM_018144 | -2.04 | ND |
| TMEM135 | AK000684 | -2.05 | ND |
| GPSM2 | AW195581 | -2.05 | ND |
| CCNJ | NM_019084 | -2.05 | ND |
| USP33 | AK023664 | -2.05 | ND |
| TAF9B | AF077053 | -2.05 | ND |
| TAF9B | AF220509 | -2.05 | ND |
| C14orf135 | NM_022495 | -2.06 | ND |
| ALG13 | NM_018466 | -2.06 | ND |
| METTL4 | NM_022840 | -2.07 | ND |
| USP48 | NM_018391 | -2.08 | ND |
| USP48 | NM_018391 | -2.08 | ND |
| RC3H2 | NM_018835 | -2.08 | ND |
| RC3H2 | NM_018835 | -2.08 | ND |
| ECHDC1 | NM_018479 | -2.10 | ND |
| NAA16 | NM_024561 | -2.11 | ND |
| TTC33 | NM_012382 | -2.13 | ND |
| RAB8B | NM_016530 | -2.14 | ND |
| CTAGE1 | NM_022663 | -2.17 | ND |
| CEP63 | AK023738 | -2.17 | ND |
| C3orf64 | AK023140 | -2.17 | ND |
| UCHL5 | NM_015984 | -2.18 | ND |
| UCHL5 | NM_016017 | -2.18 | ND |
| HMGCS1 | BG035985 | -2.19 | ND |
| ZNF692 | NM_017865 | -2.19 | ND |
| TOX | NM_014729 | -2.19 | ND |
| VCAN | BF218922 | -2.19 | ND |
| RRP15 | NM_016052 | -2.20 | ND |
| EXOC1 | AK023461 | -2.21 | ND |
| ZNF180 | NM_013256 | -2.21 | ND |
| BNC2 | NM_017637 | -2.21 | ND |
| KPNA3 | AL120704 | -2.21 | ND |
| KPNA3 | AF034756 | -2.21 | ND |
| C12orf11 | AF274950 | -2.21 | ND |
| PPP4R2 | NM_019853 | -2.21 | ND |
| E2F8 | NM_024680 | -2.22 | ND |
| C14orf45 | NM_025057 | -2.22 | ND |
| MBD5 | NM_018328 | -2.24 | ND |
| GPATCH2 | NM_018040 | -2.25 | ND |
| MPHOSPH8 | BC003542 | -2.25 | ND |
| TWSG1 | NM_020648 | -2.25 | ND |
| TMEM80 | AI739035 | -2.25 | ND |
| PTER | NM_030664 | -2.26 | ND |
| XPA | NM_000380 | -2.26 | ND |
| ZNF331 | NM_018555 | -2.26 | ND |
| BRWD1 | NM_018963 | -2.28 | ND |
| SLC38A2 | NM_018976 | -2.28 | ND |
| EFHC1 | NM_018100 | -2.28 | ND |
| MYCBP2 | AA488899 | -2.29 | ND |
| PER3 | NM_016831 | -2.29 | ND |
| TRIM45 | NM_025188 | -2.33 | ND |
| PAPOLA | AI984479 | -2.33 | ND |
| FAM45A | BE565675 | -2.33 | ND |
| SYNJ2BP | NM_018373 | -2.33 | ND |
| ATG2B | NM_018036 | -2.34 | ND |
| LRRC19 | NM_022901 | -2.34 | ND |
| ACADSB | NM_001609 | -2.34 | ND |
| KLHL24 | AW006750 | -2.35 | ND |
| PBK | NM_018492 | -2.35 | ND |
| SGK3 | NM_013257 | -2.35 | ND |
| FLRT2 | AF169676 | -2.36 | ND |
| TBL1XR1 | NM_030921 | -2.36 | ND |
| AMIGO2 | AC004010 | -2.38 | ND |
| PBRM1 | NM_018165 | -2.38 | ND |
| PBRM1 | NM_018313 | -2.38 | ND |
| TUBD1 | NM_016261 | -2.38 | ND |
| ZNF529 | AL109722 | -2.38 | ND |
| MAP7D3 | NM_024765 | -2.39 | ND |
| MAP7D3 | NM_024597 | -2.39 | ND |
| SMEK2 | BG540048 | -2.39 | ND |
| C7orf58 | NM_024913 | -2.39 | ND |
| LIN7C | NM_018362 | -2.40 | ND |
| LIN7C | AF090900 | -2.40 | ND |
| RSRC1 | NM_016625 | -2.40 | ND |
| DSCC1 | NM_024094 | -2.44 | ND |
| DCLRE1C | NM_022487 | -2.44 | ND |
| DCLRE1C | AK022922 | -2.44 | ND |
| MCM10 | NM_018518 | -2.44 | ND |
| IPW | AW770748 | -2.45 | ND |
| FASTKD1 | NM_024622 | -2.46 | ND |
| C1GALT1 | NM_020156 | -2.46 | ND |
| BBS7 | NM_018190 | -2.46 | ND |
| THUMPD2 | NM_025264 | -2.46 | ND |
| BBS10 | NM_024685 | -2.47 | ND |
| NHLRC2 | NM_017687 | -2.48 | ND |
| TNPO1 | AI307759 | -2.50 | ND |
| GNPTAB | NM_024312 | -2.50 | ND |
| MAP3K2 | AF239798 | -2.51 | ND |
| AP1AR | NM_018569 | -2.51 | ND |
| PALB2 | NM_024675 | -2.51 | ND |
| ZNF322B | NM_024639 | -2.52 | ND |
| BRCC3 | NM_024332 | -2.53 | ND |
| ERCC6L | NM_017669 | -2.53 | ND |
| ZNF606 | NM_025027 | -2.55 | ND |
| SCRN3 | NM_024583 | -2.56 | ND |
| ZNF177 | NM_003451 | -2.57 | ND |
| ZNF184 | AI811577 | -2.58 | ND |
| CCNL1 | NM_020307 | -2.61 | ND |
| SCYL2 | NM_017988 | -2.64 | ND |
| TLR6 | NM_006068 | -2.64 | ND |
| DNAJC10 | BG168666 | -2.64 | ND |
| DNAJC10 | BG168666 | -2.64 | ND |
| IFT74 | NM_025103 | -2.64 | ND |
| IFT74 | AI610355 | -2.64 | ND |
| TRIM52 | AA205660 | -2.65 | ND |
| C12orf48 | NM_017915 | -2.66 | ND |
| HIPK3 | AW291829 | -2.68 | ND |
| QSER1 | NM_024774 | -2.69 | ND |
| PILRB | NM_013440 | -2.69 | ND |
| ZNF767 | NM_024910 | -2.70 | ND |
| SIKE1 | BC005934 | -2.70 | ND |
| ZNF286A | NM_020652 | -2.71 | ND |
| SBNO1 | AK024128 | -2.71 | ND |
| FAM118A | NM_017911 | -2.72 | ND |
| RBM26 | NM_018605 | -2.72 | ND |
| MYO15B | AI825877 | -2.73 | ND |
| HCFC2 | NM_013320 | -2.73 | ND |
| ZNF701 | NM_018260 | -2.75 | ND |
| SRBD1 | NM_018079 | -2.75 | ND |
| LIMCH1 | AK027231 | -2.75 | ND |
| GIPC2 | NM_017655 | -2.75 | ND |
| ZCCHC6 | NM_024617 | -2.77 | ND |
| ZMYM6 | NM_007167 | -2.77 | ND |
| MYO5C | NM_018728 | -2.78 | ND |
| ZNF350 | NM_021632 | -2.80 | ND |
| CSNK1G3 | NM_004384 | -2.81 | ND |
| ZNF23 | AL567808 | -2.81 | ND |
| ZNF440 | AK021474 | -2.81 | ND |
| TAF1D | BC001972 | -2.82 | ND |
| PALMD | NM_017734 | -2.84 | ND |
| NCRNA00115 | NM_024796 | -2.84 | ND |
| PYROXD1 | NM_024854 | -2.84 | ND |
| KRIT1 | NM_004912 | -2.87 | ND |
| CENPQ | NM_018132 | -2.88 | ND |
| FGD6 | NM_018351 | -2.90 | ND |
| DCAF17 | NM_025000 | -2.91 | ND |
| CCDC99 | AF269167 | -2.91 | ND |
| ZBTB38 | NM_024724 | -2.92 | ND |
| WDR19 | NM_025132 | -2.93 | ND |
| SETD2 | NM_014159 | -2.93 | ND |
| SERINC4 | NM_025165 | -2.94 | ND |
| SERINC4 | NM_025165 | -2.94 | ND |
| DENND1B | NM_019049 | -2.95 | ND |
| YOD1 | AF090896 | -2.96 | ND |
| USP47 | BE966019 | -2.97 | ND |
| SSX2IP | AU152583 | -2.99 | ND |
| RAD54B | NM_012415 | -2.99 | ND |
| RAD54B | NM_006550 | -2.99 | ND |
| IFT81 | NM_014055 | -3.01 | ND |
| ZNF432 | NM_014650 | -3.02 | ND |
| ZMYM1 | NM_024772 | -3.02 | ND |
| PHF20L1 | AK022280 | -3.03 | ND |
| EDEM3 | NM_017992 | -3.03 | ND |
| EDEM3 | NM_025191 | -3.03 | ND |
| PLEKHA5 | NM_019012 | -3.05 | ND |
| GIN1 | NM_017676 | -3.07 | ND |
| CEP97 | NM_024548 | -3.11 | ND |
| FNIP1 | NM_016340 | -3.11 | ND |
| FAT4 | NM_024582 | -3.14 | ND |
| TLR4 | NM_003266 | -3.15 | ND |
| KIAA1598 | AU157109 | -3.21 | ND |
| KIAA0586 | NM_014749 | -3.23 | ND |
| GCFC1 | NM_013329 | -3.25 | ND |
| HELLS | NM_018063 | -3.26 | ND |
| MDM1 | NM_020128 | -3.30 | ND |
| USP25 | NM_013396 | -3.31 | ND |
| IL1RAPL1 | NM_014271 | -3.35 | ND |
| FER | NM_005246 | -3.36 | ND |
| RMI1 | NM_024945 | -3.36 | ND |
| RPS6KA5 | AF074393 | -3.39 | ND |
| GRAMD1C | NM_017577 | -3.39 | ND |
| ZNF14 | NM_021030 | -3.41 | ND |
| CSAD | NM_015989 | -3.42 | ND |
| ZNF267 | AU150728 | -3.44 | ND |
| CCDC121 | NM_024584 | -3.48 | ND |
| MANEA | NM_024641 | -3.50 | ND |
| ECT2 | NM_018098 | -3.54 | ND |
| MAP2K6 | NM_002758 | -3.55 | ND |
| ZNF614 | NM_025040 | -3.57 | ND |
| CENPJ | NM_018451 | -3.59 | ND |
| MYO9A | NM_006901 | -3.59 | ND |
| PPP1R9A | NM_017650 | -3.60 | ND |
| NAA15 | NM_025085 | -3.60 | ND |
| FAM35A | NM_019054 | -3.61 | ND |
| ZNF12 | NM_016265 | -3.64 | ND |
| MME | AI433463 | -3.64 | ND |
| MTUS1 | BE552421 | -3.66 | ND |
| DNAJB14 | NM_024920 | -3.67 | ND |
| CEP70 | NM_024491 | -3.75 | ND |
| CSPP1 | NM_024790 | -3.76 | ND |
| CSPP1 | BE044503 | -3.76 | ND |
| ARID4B | NM_016374 | -3.76 | ND |
| ZNF484 | BE567977 | -3.77 | ND |
| DDX60 | NM_017631 | -3.79 | ND |
| CCDC76 | NM_019083 | -3.90 | ND |
| RNF219 | NM_024546 | -3.93 | ND |
| UBA6 | NM_018496 | -3.98 | ND |
| CCDC15 | NM_025004 | -4.00 | ND |
| ZNF224 | NM_005774 | -4.01 | ND |
| ZDHHC11 | AF267859 | -4.01 | ND |
| MLH3 | AB039667 | -4.05 | ND |
| N4BP2L2 | AI809961 | -4.06 | ND |
| RUNX1T1 | X79990 | -4.09 | ND |
| CCPG1 | NM_004748 | -4.17 | ND |
| CCPG1 | AB033080 | -4.17 | ND |
| CCPG1 | AK022459 | -4.17 | ND |
| BRIP1 | AF360549 | -4.18 | ND |
| RAPGEF6 | NM_016340 | -4.24 | ND |
| LUC7L3 | NM_016424 | -4.29 | ND |
| NEK1 | AI936517 | -4.32 | ND |
| ETAA1 | NM_019002 | -4.32 | ND |
| POLI | NM_007195 | -4.37 | ND |
| CCDC41 | NM_016122 | -4.52 | ND |
| ZNF654 | NM_018293 | -4.55 | ND |
| HMGN5 | NM_030763 | -4.55 | ND |
| HMGN5 | BC005342 | -4.55 | ND |
| ALS2CR8 | NM_024744 | -4.69 | ND |
| MEG3 | AI133721 | -4.70 | ND |
| THOC2 | BG403671 | -4.77 | ND |
| PPFIBP2 | AK001131 | -4.81 | ND |
| DEPDC1 | NM_017779 | -4.83 | ND |
| APC | S67788 | -4.86 | ND |
| DST | BG253119 | -4.89 | ND |
| ZFP112 | AC084239 | -4.91 | ND |
| ZNF43 | AK022905 | -4.92 | ND |
| ZNF254 | NM_004876 | -5.01 | ND |
| PGAP1 | NM_024989 | -5.01 | ND |
| DYNC2H1 | NM_024606 | -5.01 | ND |
| TMEM144 | NM_018342 | -5.06 | ND |
| SENP7 | NM_020654 | -5.12 | ND |
| PRPF39 | NM_018333 | -5.17 | ND |
| FER1L4 | AF218012 | -5.30 | ND |
| MPHOSPH9 | AI990326 | -5.34 | ND |
| SEMA6A | NM_020796 | -5.42 | ND |
| RALGPS2 | NM_018037 | -5.59 | ND |
| KIF15 | NM_020242 | -5.67 | ND |
| C10orf118 | NM_018017 | -5.75 | ND |
| AHI1 | NM_017651 | -5.77 | ND |
| AHI1 | AL136797 | -5.77 | ND |
| ANKRD36B | NM_025190 | -5.80 | ND |
| KIF18A | NM_031217 | -5.89 | ND |
| JMJD1C | AI694023 | -6.03 | ND |
| ZNF83 | M27877 | -6.12 | ND |
| ARHGAP28 | NM_030672 | -6.14 | ND |
| ARHGAP28 | NM_030672 | -6.14 | ND |
| POLQ | NM_006596 | -6.23 | ND |
| ANKRD36 | AB046861 | -6.75 | ND |
| CCDC88A | NM_017571 | -6.78 | ND |
| CCDC88A | NM_018084 | -6.78 | ND |
| ATAD5 | NM_024857 | -7.14 | ND |
| PPL | NM_002705 | -7.21 | ND |
| EEA1 | AI916242 | -7.35 | ND |
| ANKRD26 | NM_014915 | -7.42 | ND |
| MNS1 | NM_018365 | -7.70 | ND |
| SAMD9 | NM_017654 | -8.15 | ND |
| ASPM | NM_018123 | -8.26 | ND |
| CEP290 | AF317887 | -8.59 | ND |
| CASP8AP2 | AB037736 | -9.09 | ND |
| WDR52 | NM_018338 | -13.76 | ND |
|  |  |  |  |
| Difference between the datasets (45) | | | |
| EGR1 | NM_001964 | 3.33 | -2.00 |
| UNC5B | AL049370 | 2.84 | -1.31 |
| SYNM | AK026420 | 2.66 | -1.62 |
| PPM1H | AB032983 | 2.35 | -1.99 |
| CEBPD | NM_005195 | 2.06 | -1.38 |
| RASSF2 | NM_014737 | 2.00 | -1.36 |
| ANKRD12 | AB020681 | -7.88 | 1.40 |
| IL6ST | BE856546 | -5.13 | 1.36 |
| MBNL2 | BE328496 | -3.91 | 1.32 |
| JAK2 | AF001362 | -3.81 | 1.37 |
| ZFX | R51161 | -3.34 | 1.32 |
| ITPR2 | AA834576 | -3.19 | 1.39 |
| ITPR2 | NM_002223 | -3.19 | 1.93 |
| DICER1 | AK001827 | -3.11 | 1.41 |
| IFI44 | NM_006417 | -3.10 | 2.88 |
| NBN | AF049895 | -3.00 | 1.32 |
| TNFSF10 | U57059 | -2.89 | 1.69 |
| TNFSF10 | AW474434 | -2.89 | 1.71 |
| TNFSF10 | NM_003810 | -2.89 | 2.06 |
| CAPRIN2 | NM_023925 | -2.84 | 1.49 |
| BIRC2 | NM_001166 | -2.72 | 2.10 |
| SEMA3C | NM_006379 | -2.56 | 1.48 |
| GNPTAB | AK001821 | -2.50 | 1.36 |
| TGFBR3 | NM_003243 | -2.45 | 1.36 |
| ITGA1 | X68742 | -2.38 | 1.47 |
| PLSCR4 | NM_020353 | -2.33 | 1.50 |
| ALCAM | AA156721 | -2.29 | 1.49 |
| ALCAM | BF242905 | -2.29 | 1.68 |
| SREK1IP1 | AW408767 | -2.29 | 1.33 |
| ITSN2 | U61167 | -2.21 | 1.40 |
| MIOS | AL136892 | -2.21 | 1.38 |
| SLC35A3 | BC005136 | -2.19 | 1.52 |
| LRP6 | AF074264 | -2.18 | 1.30 |
| USP24 | AB028980 | -2.17 | 1.39 |
| USP24 | BF444943 | -2.17 | 1.53 |
| DDX17 | NM_030881 | -2.14 | 1.38 |
| ACSL3 | NM_004457 | -2.10 | 1.38 |
| ACSL3 | D89053 | -2.10 | 1.43 |
| ACSL3 | AL525798 | -2.10 | 1.52 |
| PTPN11 | NM_002834 | -2.05 | 1.65 |
| RGS4 | BC000737 | -2.05 | 1.30 |
| TMEM135 | AF080569 | -2.05 | 1.34 |
| AIM1 | U83115 | -2.01 | 1.59 |
| STK17B | AA203487 | -2.00 | 1.39 |
| STK17B | NM_004226 | -2.00 | 1.39 |
| SLC25A30 | AL359557 | -2.04 | ND |
| DDX17 | AW188131 | -2.14 | ND |
| LEPR | NM_002303 | -2.18 | ND |
| MLLT3 | NM_004529 | -2.21 | ND |
| KLHL24 | NM_017644 | -2.35 | ND |
| ADAM28 | NM_021777 | -2.38 | ND |
| CLOCK | AW238724 | -2.50 | ND |
| ZBTB1 | NM_014950 | -2.51 | ND |
| IQGAP2 | NM_006633 | -2.57 | ND |
| ELK4 | NM_021795 | -2.66 | ND |
| PHC3 | AK023029 | -2.67 | ND |
| ZNF141 | NM_003441 | -2.71 | ND |
| SEMA3D | AA343027 | -2.74 | ND |
| FAM13A | AK027138 | -2.91 | ND |
| DMD | M92650 | -2.92 | ND |
| ZNF234 | AW264320 | -2.98 | ND |
| RASA2 | NM_006506 | -3.02 | ND |
| FAM63B | AL049226 | -3.03 | ND |
| ZNF507 | NM_014910 | -3.03 | ND |
| CDC42BPA | AA127643 | -3.16 | ND |
| MDM4 | NM_002393 | -3.20 | ND |
| GUCY1B3 | W93728 | -3.20 | ND |
| CDRT1 | U43383 | -3.25 | ND |
| XAF1 | NM_017523 | -3.30 | ND |
| CFH | X04697 | -3.39 | ND |
| ZCCHC11 | R25849 | -3.46 | ND |
| FOXN2 | NM_002158 | -3.54 | ND |
| LARP7 | AK000089 | -3.75 | ND |
| ZNF451 | AU144775 | -3.99 | ND |
| ZNF224 | BC002889 | -4.01 | ND |
| MLH3 | AC006530 | -4.05 | ND |
| ZNF137P | NM_003438 | -4.12 | ND |
| CCPG1 | AU144243 | -4.17 | ND |
| DNM3 | AL136712 | -4.87 | ND |
| DST | NM_001723 | -4.89 | ND |
| C8orf84 | BE968773 | -5.89 | ND |
| KIAA1107 | AB029030 | -6.02 | ND |
| ZNF493 | BC006408 | -6.84 | ND |
| ATRX | BC002521 | -7.30 | ND |
| DGKD | NM_003648 | 2.08 | ND |
| TMEM231 | NM_024533 | 2.02 | ND |
| C3orf52 | NM_024616 | 2.13 | ND |
| RAB11FIP1 | NM_025151 | 2.22 | ND |
| PRR16 | NM_016644 | 3.46 | ND |
| CCRN4L | NM_012118 | 2.34 | ND |
| NUAK2 | NM_030952 | 2.58 | ND |
| SOD2 | BF575213 | 2.35 | ND |
| SPRY4 | W48843 | 2.45 | ND |
| ETV7 | AF147782 | 3.09 | ND |
| RND1 | U69563 | 4.49 | ND |
